# On-Line Screening, Isolation and Identification of Antioxidant Compounds of *Helianthemum ruficomum*

**DOI:** 10.3390/molecules22020239

**Published:** 2017-02-08

**Authors:** Yasmine Chemam, Samir Benayache, Eric Marchioni, Minjie Zhao, Paul Mosset, Fadila Benayache

**Affiliations:** 1 Unité de Recherche Valorisation des Ressources Naturelles, Molécules Bioactives et Analyses Physicochimiques et Biologiques, Université des Frères Mentouri, Constantine 1, Route d’Aïn El Bey, 25000 Constantine, Algeria; ch.jasmin@hotmail.fr (Y.C.); sbenayache@yahoo.com (S.B.); 2 Chimie Analytique des Molécules Bioactives, Institut Pluridisciplinaire Hubert Curien (UMR 7178 CNRS/UDS), 74 route du Rhin, 67400 Illkirch, France; Eric.Marchioni@unistra.fr (E.M.); minjzhao@unistra.fr (M.Z.); 3 Institut des Sciences Chimiques de Rennes, CNRS UMR 6226, Université de Rennes 1, 263 Avenue du Général Leclerc, CS 74205, 35042 Rennes CEDEX, France; paul.mosset.1@univ-rennes1.fr

**Keywords:** *Helianthemum ruficomum*, Cistaceae, antioxidant activity, HPLC-ABTS^•+^, TEAC, ORAC

## Abstract

Many *Helianthemum* species (Cistaceae) are recognized for their various medicinal virtues. *Helianthemum ruficomum* is an endemic species to the septentrional Sahara on which no report is available so far. The purpose of this work was to investigate the chemical composition and the radical scavenging capacity of this species and its isolated components. Collected from Mougheul (south-west of Algeria), the aerial parts were macerated with 80% EtOH/H_2_O, after evaporation, the remaining extract was diluted with H_2_O and extracted with petroleum ether, chloroform, ethyl acetate and *n*-butanol. EtOAc and *n*-BuOH extracts were evaluated for their free radical scavenging capacity by on-line HPLC-ABTS^•+^ assay. The obtained data which were confirmed by TEAC and ORAC assays, allowed guiding the fractionation of these extracts by CC, TLC and reverse phase HPLC. Among the components, 14 were isolated and identified by spectroscopic analyses: protocatechuic acid (**1**), *trans*-tiliroside (**2**), *cis*-tiliroside (**3**), astragalin (**4**), picein (**7**), vanillic acid 4-*O*-β-d-glucopyranoside (**8**), lavandoside (**9**), 4-hydroxybenzoic acid 4-*O*-β-d-glucopyranoside (**10**), nicotiflorin (**11**), rutin (**12**), vicenin-*2* (**13**), narcissin (**14**) and stigmasterol (**5**) and β-sitosterol (**6**) as a mixture (71% and 29%, respectively). Compounds **5**, **7**, **8**, **9**, **10** and **14** were new for the genus *Helianthemum*. The antioxidant power of all the isolated compounds was also evaluated by HPLC-ABTS^•+^, TEAC and ORAC assays. The results clearly indicated high antioxidant potential of the extracts and tested compounds of this species especially, compounds **1**, **4**, **8**, **9**, **10** and **12**.

## 1. Introduction

Currently, there is an increasing interest in the research and prospection of new sources of natural antioxidants as safe additives in food industry or ingredients of functional foods, neutraceuticals and pharmaceuticals. Free radicals are major contributors in aging and play a key role in degenerative diseases [1,2,3]. Sahara species can develop metabolite responses against drought stress and ROS produced by extensive UV exposition [4,5,6]. According to this finding and the results of our previous studies on Saharan species which showed the presence of high content of bioactive compounds and positive antioxidant and antiproliferative properties [7,8,9,10], we investigate in this study, extracts of an endemic species of the septentrional Sahara, *Helianthemum ruficomum* from the Cistaceae family by on-line HPLC-ABTS^•+^ method in order to evaluate their antioxidant capacities.

Cistaceae consists of 8 genera and about 180 species [11]. *Helianthemum* genus (Cistaceae) contains approximately 110 species [12], some of them are important medicinal plants used in several countries for different purposes [13,14,15,16,17]. This genus is reported to possess anti-inflammatory, antimicrobial, antiprotozoal and antioxidant properties [18,19,20,21,22]. Even if this genus was not studied much from the phytochemical viewpoint, some species have been previously examined for bioactive components like flavonoids, phenolic acids, lignans and essential oils [23,24,25,26,27,28].

To the best of our knowledge, the species *Helianthemum ruficomum* (Viv.) Spreng (synonyms: *Cistus ruficomus* Viv., *Helianthemum arnaizii* Sennen, *H. desiderii* Sennen, *H. hirtum* subsp. *ruficomum* (Viv.) Maire, *H. eremophilum* Pomel, *H. hirtum* var. *deserti* Coss., *H. desertorum* Willk. [29,30,31] has not been previously studied. In this work, ethyl acetate and *n*-butanol soluble parts of the aqueous-EtOH extract of the aerial parts were investigated using liquid chromatography with post-column reaction allowing a direct on-line detection of radical scavenging power of molecular species. A special focus was done on the evaluation of free radical scavenging capacities (ABTS^•+^, TEAC, ORAC assays and on-line HPLC-ABTS^•+^) of extracts and isolated compounds. The structures of the isolated compounds were elucidated using ESI-HRMS, molecular absorption spectroscopy, extensive application of one- and two-dimensional NMR spectroscopy and comparison with literature data.

## 2. Results and Discussion

### 2.1. Isolation and Structure Elucidation of Compounds

**Figure 1:**
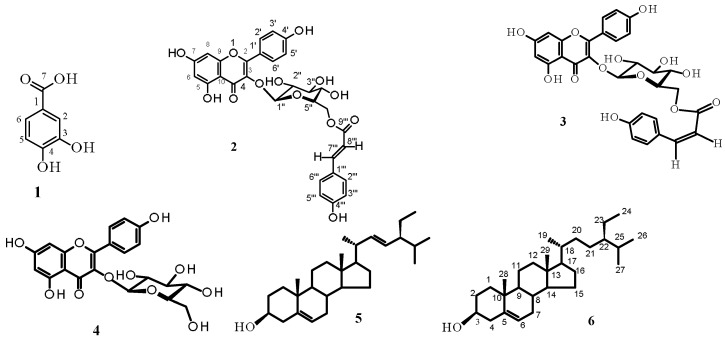

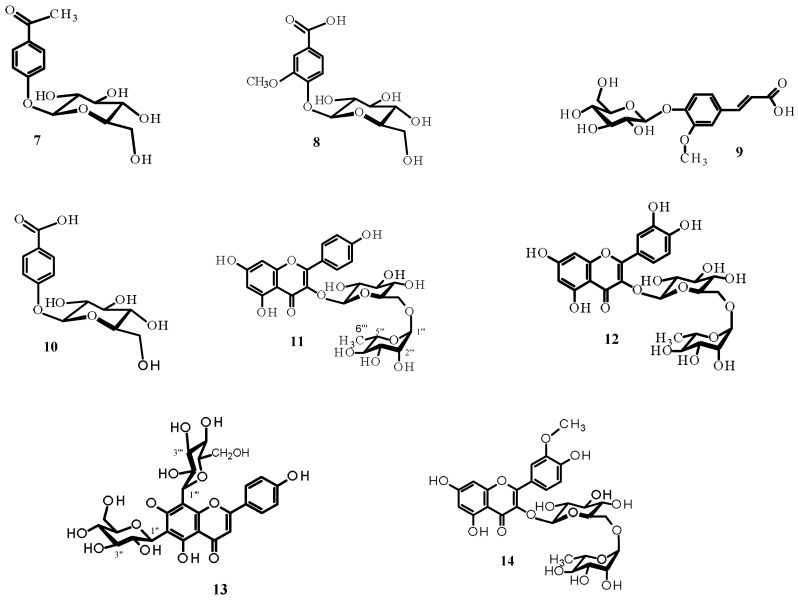
Structures of compounds (**1**–**14**) isolated from *Helianthemum ruficomum*.

Chromatographic procedures (CC and TLC of silica gel 60 and semi-preparative reverse phase HPLC) led to the isolation from the aerial parts of *Helianthemum ruficomum*, of five phenolic acids: protocatechuic acid (**1**) [32], picein (**7**) [33], vanillic acid 4-*O*-β-d-glucopyranoside (**8**) [34], lavandoside (*trans*-Ferulic acid 4-*O*-β-d-glucopyranoside) **9** [35,36], 4-hydroxybenzoic acid 4-*O*-β-d-glucopyranoside (**10**) [37]; seven flavonoid glycosides: *trans*-tiliroside (**2**) [38,39], slightly contamined by its stereoisomer *cis*-tiliroside, *cis*-tiliroside (**3**) [38,39] contamined by *trans*-tiliroside, astragalin (**4**) [40], nicotiflorin (**11**) [41], rutin (**12**) [41], vicenin-*2* (**13**) [42], narcissin (**14**) [41], and a mixture (71%–29%) of stigmasterol (**5**) and β-sitosterol (**6**), respectively [43], (Figure 1). The compounds were identified by spectral analysis, mainly ESI-HRMS, UV and NMR experiments (^1^H, ^13^C, DEPT, DOSY, COSY, NOESY, HSQC and HMBC) and comparison of their spectroscopic data with those reported in the literature. To the best of our knowledge, compounds **5**, **7**, **8**, **9**, **10** and **14** were new for the genus *Helianthemum*. In addition, all the isolated compounds were described for the first time from this species. The spectra of all the compounds are available in the Supplementary Materials.

*Protocatechuic acid* (**1**): ESI-HRMS(+): *m/z* 155.03358 [M + H]^+^, accurate mass 154.0263; formula: C_7_H_6_O_4_; ^1^H-NMR (400 MHz, CD_3_OD) δ_H_ (ppm) 7.35 (1H, d, *J* = 1.8 Hz, H-*2*), 7.30 (1H, dd, *J* = 8.0, 1.8 Hz, H-6), 6.72 (1H, d, *J* = 8.0 Hz, H-5); ^13^C-NMR (100 MHz, CD_3_OD) δ_C_ (ppm) 168.87 (C-7), 149.76 (C-4), 144.72 (C-3), 123.09 (C-1), 122.56 (C-6), 116.91 (C-2), 114.95 (C-5).

*trans*-*Tiliroside* (**2**): Yellow powder, ESI-HRMS(+): *m/z* 617.12741 [M + Na]^+^, 595.14507 [M + H]^+^, formula: C_30_H_26_O_13_; ^1^H-NMR (400 MHz, DMSO-*d*_6_) δ_H_ (ppm) aglycone: 7.98 (2H, d, *J* = 8.8 Hz, H-2′, H-6′), 6.84 (2H, d, *J* = 8.8 Hz, H-3′, H-5′), 6.26 (1H, d, *J* = 1.6 Hz, H-8), 6.05 (1H, d, *J* = 1.6 Hz, H-6), sugar moiety: 5.41 (1H, d, *J* = 6.8 Hz, H-1″), 4.28 (1H, dd, *J* = 12.0, 2.8 Hz, H-6″a), 4.03 (1H, dd, *J* =12.0, 5.2 Hz, H-6″b), 3.40 (1H, m, H-5″), 3.30 (1H, m, H-3″), 3.25 (1H, m, H-2″), 3.19 (1H, m, H-4″), *p*-coumaroyl moiety: 7.38 (2H, d, *J* = 8.8 Hz, H-2′′′, H-6′′′), 7.36 (1H, d, *J* = 16.0 Hz, H-7′′′), 6.79 (2H, d, *J* = 8.8 Hz, H-3′′′, H-5′′′), 6.13 (1H, d, *J* = 16.0 Hz, H-8′′′); ^13^C-NMR (100 MHz, DMSO-*d*_6_) δ_C_ (ppm) aglycone: 177.31 (C-4), 166.69 (C-7), 161.54 (C-5), 160.38 (C-4′), 157.07 (C-9), 157,06 (C-2), 133.40 (C-3), 131.18 (C-2′, C-6′), 121.28 (C-1′), 115,53 (C-3′, C-5′), 103.30 (C-10), 99.99 (C-6), 94.55 (C-8), sugar moiety: 101.79 (C-1″), 76.71 (C-3″), 74.66 (C-5″), 74.60 (C-2″), 70.39 (C-4″), 63.46 (C-6″), *p*-coumaroyl moiety: 166.69 (C-9′′′), 156.36 (C-4′′′), 145.13 (C-7′′′), 130.64 (C-2′′′, C-6′′′), 125.30 (C-1′′′), 116.31 (C-3′′′, C-5′′′), 114.04 (C-8′′′). This compound was slightly contamined by its stereoisomer *cis*-tiliroside.

*cis*-*Tiliroside* (**3**): This compound was obtained as a mixture with *trans*-tiliroside (**2**) (42% *trans*-tiliroside- 58% *cis*-tiliroside). Yellow powder, ESI-HRMS(+): *m/z* 595.14494 [M + H]^+^, formula: C_30_H_26_O_13_; ^1^H-NMR (500 MHz, CD_3_OD) δ_H_ (ppm) aglycone : 7.99 (2H, d, *J* = 8.9 Hz, H-2′, H-6′), 6.80 (2H, d, *J* = 8.9 Hz, H-3′, H-5′), 6.31(1H, d, *J* = 2.0 Hz, H-8), 6.18 (1H, d, *J* = 2.0 Hz, H-6), sugar moiety: 5.18 (1H, d, *J* = 7.5 Hz, H-1″), 4.28 (1H, dd, *J* = 12.0, 2.8 Hz, H-6″a), 4.18 (1H, dd, *J* = 12.0, 5.2 Hz, H-6″b), 3.45 (1H, m, H-5″), 3.43 (1H, m, H-3″), 3.40 (1H, m, H-2″), 3.28 (1H, m, H-4″), *p*-coumaroyl moiety: 7.51(2H, d, *J* = 8.8 Hz, H-2′′′, H-6′′′), 7.31(2H, d, *J* = 8.0 Hz, H-3′′′, H-5′′′), 6.80 (1H, d, *J* = 12.3 Hz, H-7′′′), 5.51(1H, d, *J* = 12.3 Hz, H-8′′′); ^13^C-NMR (125 MHz, CD_3_OD) δ_C_ (ppm) aglycone: 177.98 (C-4), 163.61 (C-7), 161.54 (C-5), 159.84 (C-4′), 157.24 (C-2),157.18 (C-9), 133.78 (C-3), 130.87 (C-2′, C-6′), 121.34 (C-1′), 115.42 (C-3′, C-5′), 102.71 (C-10), 97.40 (C-6), 94.91 (C-8), sugar moiety: 101.94 (C-1″), 76.61 (C-3″), 74.27 (C-5″), 74.15 (C-2″), 70.30 (C-4″), 62.65 (C-6″), *p*-*c*oumaroyl moiety: 166.35 (C-9′′′), 159.68 (C-4′′′), 132.37 (C-2′′′, C-6′′′), 129.80 (C-3′′′, C-5′′′), 126.15 (C-1′′′), 114.65 (C-7′′′), 114.34 (C-8′′′).

*Astragalin* (**4**): ESI-HRMS(+): *m/z* 449.10709 [M + H]^+^, formula: C_21_H_20_O_11_; ^1^H-NMR (400 MHz, CD_3_OD) δ_H_ (ppm) aglycone: 7.94 (2H, d, *J* = 8.8 Hz, H-2′, H-6′), 6.79 (2H, d, *J* = 8.8 Hz, H-3′, H-5′), 6.28 (1H, d, *J* = 2.0 Hz, H-8), 6.09 (1H, d, *J* = 2.0 Hz, H-6), sugar moiety: 5.13 (1H, d, *J* = 7.3 Hz, H-1″), 4.61 (1H, dd, *J* = 12.0, 2.4 Hz, H-6″a), 4.46 (1H, dd, *J* = 12.0, 5.2 Hz, H-6″b), 3.35 (1H, m, H-2″), 3.34 (1H, m, H-3″), 3.24 (1H, t, *J* = 8.0 Hz, H-4″), 3.12 (1H, m, H-5″); ^13^C-NMR (100 MHz, CD_3_OD) δ_C_ (ppm) aglycone: 178.06 (C-4), 164.65 (C-7), 161.52 (C-5), 160.27 (C-4′), 157.73 (C-2), 157.04 (C-9), 134.15 (C-3), 131.05 (C-2′, C-6′), 121.33 (C-1′), 114.91 (C-3′, C-5′), 104.36 (C-10), 98.74 (C-6), 93.64 (C-8), sugar moiety: 102.79 (C-1″), 77.06 (C-5″), 76.64 (C-3″), 74.34 (C-2″), 69.98 (C-4″), 61.21 (C-6″).

*Stigmasterol* (**5**): This compound was obtained as a mixture with the compound **6**; HRMS: *m/z* 412.3713 [M]^+^, formula: C_29_H_48_O; ^1^H-NMR (500 MHz, CDCl_3_) δ (ppm): 5.28 (1H, brd, *J* = 5.1 Hz, H-6), 5.11 (1H, dd, *J* =14.9, 8.5 Hz, H-21), 4.97 (1H, dd, *J* = 14.9, 8.2 Hz, H-20), 3.45 (1H, m, H-3), 1.03 (3H, s, H_3_-29), 0.94 (3H, d, *J* = 6.2 Hz H_3_-19), 0.88 (3H, t, *J* = 7.1 Hz, H_3_-24), 0.86 (3H, d, *J* = 6.6 Hz, H_3_-26), 0.82 (3H, d, *J* = 6.6 Hz, H_3_-27), 0.73 (3H, s, H-28); ^13^C-NMR (125 MHz, CDCl_3_) δ_C_ (ppm) 141.12 (C, C-5), 137.95 (CH, C-20), 129.93 (CH, C-21), 121.73 (CH, C-6), 72.22 (CH, C-3), 56.73 (CH, C-14), 56.18 (CH, C-17), 50.25 (CH, C-9), 46.13 (CH, C-22), 42.40 (CH_2_, C-4), 42.38 (C, C-13), 40.60 (CH, C-18), 39.92 (CH_2_, C-12), 37.63 (CH_2_, C-1), 36.60 (C, C-10), 32.11 (CH_2_, C-2), 31.83 (CH_2_ and CH, C-7 and C-8), 29.64 (CH, C-25), 29.31 (CH_2_, C-16), 25.45 (CH_2_, C-23), 24.44 (CH_2_, C-15), 21.72 (CH_3_, C-19), 21.50 (CH_2_, C-11), 20.23 (CH_3_, C-26), 19.82 (CH_3_, C-27), 18.93 (CH_3_, C-28), 12.25 (CH_3_, C-29), 12.14 (CH_3_, C-24).

β-*Sitosterol* (**6**): White crystals; HRMS(+): *m/z* 414.3864 [M]^+^, formula: C_29_H_50_O; ^1^H-NMR (500 MHz, CDCl_3_) δ_H_ (ppm) 5.34 (1H, brd, *J* = 6.4 Hz, H-6), 3.54 (1H, m, H-3), 1.02 (3H, s, H_3_-29), 0.94 (3H, d, *J* = 6.5 Hz, H_3_-19), 0.85 (3H, d, *J* = 7.2 Hz, H_3_-24), 0.84 (3H, t, *J* = 6.4 Hz, H_3_-26), 0.81 (3H, d, *J* = 6.4 Hz, H_3_-27), 0.68 (3H, s, H_3_-28); ^13^C-NMR (125 MHz, CDCl3) δ_C_ (ppm) 140.75 (C, C-5), 121.63 (CH, C-6), 71.80 (CH, C-3), 56.78 (CH, C-14), 56.12 (CH, C-17), 50.19 (CH, C-9), 45.89 (CH, C-22), 42.34 (C, C-13), 42.34 (CH_2_, C-4), 39.83 (CH_2_, C-12), 37.28 (CH_2_, C-1), 36.52 (C, C-10), 36.14 (CH, C-18), 33.99 (CH_2_, C-20), 31.92 (CH_2_ and CH, C-7 and C-8), 31.65 (CH_2_, C-2), 29.24 (CH, C-25), 28.24 (CH_2_, C-16), 26.20 (CH_2_, C-21), 24.30 (CH_2_, C-15), 23.13 (CH_2_, C-23), 21.12 (CH_2_, C-11), 19.79 (CH_3_, C-28), 19.38 (CH_3_, C-27), 19.07 (CH_3_, C-26), 18.78 (CH_3_, C-19), 11.98 (CH_3_, C-24), 11.86 (CH_3_, C-29). This compound was obtained as a mixture with stigmasterol (**5**) (29 and 71%, respectively).

*Picein* (**7**): ESI-HRMS(+): *m/z* 321.09386 [M + Na]^+^, 619.19864 [2M + Na]^+^, accurate mass 298.10461; formula: C_14_H_18_O_7_, *m/z* 137.05910 [M + H − 162]^+^ indicating the presence of a hexose moiety linked to the rest of the molecule by an *O*-glycosidic bond; ^1^H-NMR (400 MHz, DMSO-*d*_6_) δ_H_ (ppm) aglycone: 7.91 (2H, d, *J* = 8.6 Hz, H-2, H-6), 7.11 (2H, d, *J* = 8.6 Hz, H-3, H-5), 2.51 (3H, s, H_3_-7), sugar moiety: 4.99 (1H, d, *J* = 7.2 Hz, H-1′), 3.69 (1H, brd, *J* = 12.1 Hz, H-6′a), 3.48 (1H, dd, *J* = 12.1, 5.9 Hz, H-6′b), 3.42 (1H, m, H-5′), 3.35 (1H, m, H-3′), 3.30 (1H, dd, *J* = 9.2, 7.2 Hz, H-2′), 3.19 (1H, t, *J* = 9.2 Hz, H-4′); ^13^C-NMR (100 MHz, DMSO-*d*_6_) δ_C_ (ppm) aglycone: 198.99 (C-7), 162.25 (C-4), 132.07 (C-1), 131.73 (C-2, C-6), 117.18 (C-3, C-5), 27.64 (C-8), sugar moiety: 100.79 (C-1′), 77.93 (C-5′), 77.26 (C-3′), 74.16 (C-2′), 70.72 (C-4′), 61.74 (C-6′).

*Vanillic acid 4-O-*β*-d-glucopyranoside* (**8**): ESI-HRMS(+): *m/z* 353.08341 [M + Na]^+^, formula: C_14_H_18_O_9_, *m/z* 169.04893 [M + H − 162]^+^ indicating the presence of a hexose moiety linked to the rest of the molecule by an *O*-glycosidic bond; ^1^H-NMR (400 MHz, CD_3_OD) δ_H_ (ppm) aglycone: 7.56 (1H, dd, *J* = 8.4, 2.0 Hz, H-6), 7.51 (1H, d, *J* = 2.0 Hz, H-2), 7.12 (1H, d, *J* = 8.4 Hz, H-5), 3.83 (3H, s, OCH_3_), sugar moiety: 5.04 (1H, d, *J* = 7.2 Hz, H-1′), 3.85 (1H, brd, *J* = 12.0 Hz, H-6′a), 3.67 (1H, dd, *J* = 12.0, 5.6 Hz, H-6′b), 3.55 (1H, t, *J* = 8 Hz, H-2′), 3.53 (1H, m, H-3′), 3.48 (1H, m, H-5′), 3.42 (1H, m, H-4′); ^13^C-NMR (100 MHz, CD_3_OD) δ_C_ (ppm) aglycone: 167.65 (C-7), 148.67 (C-4), 146.96 (C-3), 123.03 (C-1), 122.35(C-6′), 113.32 (C-5), 111.59 (C-2), 54.21 (OCH_3_), sugar moiety: 98.72 (C-1′), 75.01 (C-5′), 74.49 (C-3′), 71.64 (C-2′), 68.17 (C-4′), 59.34 (C-6′).

*Lavandoside* (*trans-ferulic acid 4-O-*β*-d-glucopyranoside*, **9**): ESI-HRMS(+): *m/z* 379.09896 [M + Na]^+^, 735.20725 [*2*M + Na]*^+^* according to the molecular formula C_16_H_20_O_9_, *m/z* 195.06445 [M + H − 162]^+^ indicating the presence of a hexose moiety linked to the rest of the molecule by an *O*-glycosidic bond; ^1^H-NMR (400 MHz, CD_3_OD) δ_H_ (ppm) aglycone: 7.62 (1H, d, *J* = 16.4 Hz, H-7), 7.25 (1H, brs, H-2), 7.19 (1H, d, *J* = 8.0 Hz, H-5), 7.18 (1H, brd, H-6), 6.41 (1H, d, *J* = 16.4 Hz, H-8), 3.91 (3H, s, OCH_3_), sugar moiety: 4.99 (1H, d, *J* = 7.3 Hz, H-1′), 3.89 (1H, dd, *J* = 10.2, 3.9 Hz, H-6′a), 3.71 (1H, dd, *J* = 10.2, 5.8 Hz, H-6′b), 3.54 (1H, m, H-2′), 3.52 (1H, t, *J* = 8.0 Hz, H-3′), 3.45 (1H, m, H-5′), 3.43 (1H, m, H-4′); ^13^C-NMR (100 MHz, CD_3_OD) δ_C_(ppm) aglycone: 169.39 (C-9), 149.58 (C-3), 148.51 (C-4), 144.45 (C-7), 129.31 (C-1), 121.99 (C-6), 116.84 (C-8), 116.01 (C-5), 111.03 (C-2), 55.40 (OCH_3_), sugar moiety: 100.82 (C-1′), 76.87 (C-5′), 76.44 (C-3′), 73.42 (C-2′), 69.89 (C-4″), 61.07 (C-6′).

*4-Hydroxybenzoic acid 4-O-*β*-d-glucopyranoside* (**10**): ESI-HRMS(−): *m/z* 299.0772 [M − H]^−^, formula: C_13_H_16_O_8_; ^1^H-NMR (500 MHz, CD_3_OD) δ_H_ (ppm) aglycone: 7.98 (2H, d, *J* = 9.0 Hz, H-2, H-6), 7.14 (2H, d, *J* = 9.0 Hz, H-3, H-5), sugar moiety: 5.03 (1H, d, *J* = 7.3 Hz, H-1′), 3.92 (1H, dd, *J* = 12.5, 2.5 Hz, H-6′a), 3.73 (1H, dd, *J* = 12.5, 5.5 Hz, H-6′b), 3.52 (1H, m, H-5′), 3.51 (1H, m, H-3′), 3.50 (1H, m, H-2′), 3.44 (1H, m, H-4′); ^13^C-NMR (125 MHz, CD_3_OD) δ_C_ (ppm) aglycone: 169.89 (C-7), 160.86 (C-4), 131.10 (C-2, C-6), 126.89 (C-1), 115.57 (C-3, C-5), sugar moiety: 100.34 (C-1′), 76.84 (C-5″), 76.55 (C-3″), 73.46 (C-2″), 69.91 (C-4′), 61.06 (C-6′).

*Nicotiflorin* (**11**): ESI-HRMS(+): *m/z* 595.16524 [M + H]^+^, 617.14788 [M + Na]^+^, formula: C_27_H_30_O_15_; ^1^H-NMR (400 MHz, DMSO-*d*_6_) δ_H_ (ppm): aglycone: 7.94 (2H, d, *J* = 8.8 Hz, H-2′, H-6′), 6.88 (d, 2H, *J* = 8.8 Hz, H-3′, H-5′), 6.38 (1H, d, *J* = 2.0 Hz, H-8), 6.16 (1H, d, *J* = 2.0 Hz, H-6), *O*-glucopyranosyl moiety: 5.34 (1H, d, *J* = 7.6 Hz, H-1″), 3.67 (1H, dd, *J* = 10.2, 3.6 Hz, H-6″a), 3.42 (1H, m, H-3″), 3.31(1H, dd, *J* = 10.2, 3.6 Hz, H-6″b), 3.25 (1H, m, H-5″), 3.04 (1H, m, H-4″), 3.23 (1H, m, H-2″), *O*-rhamnopyranosyl moiety: 4.40 (1H, d, *J* = 1.2 Hz, H-1′′′), 3.42 (1H, m, H-2′′′), 3.24 (1H, m, H-3′′′), 3.21 (1H, m, H-5′′′), 3.06 (1H, m, H-4′′′), 0.92 (3H, d, *J* = 6.4 Hz, H_3_-6′′′); ^13^C-NMR (100 MHz, DMSO-*d*_6_) δ_C_ (ppm) aglycone: 177.67 (C-4), 164.22 (C-7), 161.14 (C-5), 159.92 (C-4′), 157.11 (C-2), 156.90 (C-9), 133.55 (C-3), 131.37 (C-2′, C-6′), 121.58 (C-1′), 115,54 (C-3′, C-5′), 104.45 (C-10), 99.08 (C-6), 94.31 (C-8), *O*-glucopyranosyl moiety: 101.70 (C-1″), 76.03 (C-5″), 72.05 (C-4″), 70.53 (C-2″), 70.34 (C-3″), 67.43 (C-6″), *O*-rhamnopyranosyl moiety: 101.19 (C-1′′′), 70.81 (C-3′′′), 70.53 (C-4′′′), 70.34 (C-2′′′), 68.64 (C-5′′′), 17.83 (C-6′′′).

*Rutin* (**12**): ESI-HRMS(+): *m/z* 611.15894 [M + H]^+^, 633.1404 [M + Na]^+^, formula: C_27_H_30_O_16_; ^1^H-NMR (400 MHz, CD_3_OD) δ_H_ (ppm) aglycone: 7.68 (1H, d, *J* = 2.4 Hz, H-2′), 7.62 (1H, dd, *J* = 8.4, 2.4 Hz, H-6′), 6.88 (1H, d, *J* = 8.4 Hz, H-5′), 6.36 (1H, d, *J* = 2.0 Hz, H-8), 6.18 (1H, d, *J* = 2.0 Hz, H-6), *O*-glucopyranosyl moiety: 5.11 (1H, d, *J* = 7.2 Hz, H-1″), 3.82 (1H, dd, *J* = 9.6, 3.6 Hz, H-6″a), 3.50 (1H, t, *J* = 8.0 Hz, H-2″), 3.43 (1H, m, H-5″), 3.39 (1H, m, H-6″b), 3.35 (1H, m, H-3″), 3.29 (1H, m, H-4″), *O*-rhamnopyranosyl moiety: 4.55 (1H, d, *J* = 1.2 Hz, H-1′′′), 3.68 (1H, m, H-2′′′), 3.57 (1H, dd, *J* = 6.1, 3.5 Hz, H-3′′′), 3.45 (1H, dd, *J* = 11.0, 2.1 Hz, H-5′′′), 3.33 (1H, m, H-4′′′), 1.15 (3H, d, *J* = 6.4 Hz, H_3_-6′′′); ^13^C-NMR (100 MHz, CD_3_OD) δ_C_ (ppm) aglycone: 177.93 (C-4), 164.50 (C-7), 161.42 (C-5), 157.92 (C-9), 157.01 (C-2), 148.36 (C-4′), 144,36 (C-3′), 134.32 (C-3), 122.29 (C-1′), 121.77 (C-6′), 116.44 (C-2′), 114.74 (C-5′), 104.25 (C-10), 98.64 (C-6), 93.60 (C-8), *O*-glucopyranosyl moiety: 103.48 (C-1″), 76.81 (C-5″), 75.77 (C-3″), 74.38 (C-2″), 70.03 (C-4″), 67.23 (C-6″), *O*-rhamnopyranosyl moiety: 101.03 (C-1′′′), 70.92 (C-3′′′), 70.71 (C-2′′′), 70.03 (C-4′′′), 68.34 (C-5′′′), 16.55 (C-6′′′).

*Vicenin-2* (**13**): HRESI-MS(+): *m/z* 595.16515 [M + H]^+^, formula: C_27_H_30_O_15_; ^1^H-NMR (400 MHz, DMSO-*d*_6_) δ_H_ (ppm) aglycone: 7.99 (2H, d, *J* = 8.4 Hz, H-2′, H6′), 6.91 (2H, d, *J* = 8.4 Hz, H-3′, H-5′), 6.76 (1H, s, H-3), 6-*C*-glucopyranosyl moiety: 4.80 (1H, d, *J* = 9.7 Hz, H-1″), 3.75 (1H, dd, *J* = 10.9, 3.4 Hz, H-6″a), 3.50 (1H, dd, *J* = 10.9, 6.2 Hz, H-6″b), 3.48 (1H, m, H-2″), 3.37 (1H, t, *J* = 8.6 Hz, H-4″), 3.30 (1H, t, *J* = 8.6 Hz, H-3″), 3.23 (1H, m, H-5″), 8-*C*-glucopyranosyl moiety: 4.75 (1H, d, *J* = 9.7 Hz, H-1′′′), 3.87 (1H, m, H-2′′′), 3.63 (2H, m, H-6′′′), 3.36 (1H, t, *J* = 8.6 Hz, H-4′′′), 3.33 (1H, m, H-5′′′), 3.28 (1H, m, H-3′′′); ^13^C-NMR (100 MHz, DMSO-*d*_6_) δ_C_ (ppm) aglycone: 182.78 (C-4), 161.60 (C-2), 160.67 (C-4′), 159.53 (C-7), 159.07 (C-5), 155.49 (C-9), 129.51 (C-2′, C-6′), 125.99 (C-1′), 116.25 (C-3′,C-5′), 103.61 (C-10), 103.16 (C-6), 103.08 (C-3), 101.27 (C-8), 6-*C*-glucopyranosyl moiety: 82.39 (C-5″), 79.32 (C-3″), 74.56 (C-1″), 72.43 (C-2″), 69.53 (C-4″), 61.92 (C-6″), 8-*C*-glucopyranosyl moiety: 81.36 (C-5′′′), 79.32 (C-3′′′), 73.82 (C-1′′′), 70.98 (C-2′′′), 69.53 (C-4′′′), 61.78 (C-6′′′).

*Narcissin* (**14**): This compound was slightly contamined by compound **9**. ESI-HRMS(+): *m/z* 625.17636 [M + H]^+^, formula: C_28_H_32_O_16_; ^1^H-NMR (400 MHz, CD_3_OD) δ_H_ (ppm) aglycone: 7.95 (1H, d, *J* = 1.6 Hz, H-2′), 7.63 (1H, dd, *J* = 8.0, 1.6 Hz, H-6′), 6.92 (1H, d, *J* = 8.0 Hz, H-5′), 6.40 (1H, d, *J* = 1.5 Hz, H-8), 6.21 (1H, d, *J* = 1.5 Hz, H-6), 3.96 (3H, s, OCH_3_-3′), *O*-glucopyranosyl moiety: 5.24 (1H, d, *J* = 7.2 Hz, H-1″), 3.81 (1H, dd, *J* = 10.4, 3.4 Hz, H-6″a), 3.47 (1H, m, H-2″), 3.45 (1H, m, H-5″), 3.41 (1H, dd, *J* = 10.4, 6.5, H-6″b), 3.39 (1H, m, H-3″), 3.28 (1H, t, *J* = 9.3 Hz, H-4″), *O*-rhamnopyranosyl: 4.55 (1H, d, *J* = 1.2 Hz, H-1′′′), 3.63 (m, 1H, H-2′′′), 3.48 (1H, m, H-3′′′), 3.27 (1H, t, *J* = 9.3 Hz, H-4′′′), 3.0 (1H, m, H-5′′′), 1.12 (3H, d, *J* = 6.2 Hz, H_3_-6′′′); ^13^C-NMR (100 MHz, CD_3_OD) δ_H_ (ppm) aglycone: 177.91 (C-4), 164.57 (C-7), 161.55 (C-5), 157.46 (C-2), 157.05 (C-9), 149.43 (C-4′), 146.60 (C-3′), 134.12 (C-3), 122.62 (C-6′), 121.60 (C-1′), 114.72 (C-5′), 113.22 (C-2′), 104.32 (C-10), 98.59 (C-6), 93.56 (C-8), 55.42 (OCH_3_-3′), *O*-glucopyranosyl moiety: 103.11 (C-1″), 76.80 (C-5″), 75.97 (C-3″), 74.54 (C-4″), 74.38 (C-2″), 67.16 (C-6″), *O*-rhamnopyranosyl: 101.11 (C-1′′′), 72.38 (C-4′′′), 70.69 (C-3′′′), 70.24 (C-2′′′), 68.39 (C-5′′′), 16.50 (C-6′′′).

### 2.2. Identification of Chromatographic Peaks and Antioxidant Activity of Plant Extracts and Pure Compounds

**Figure 2:**
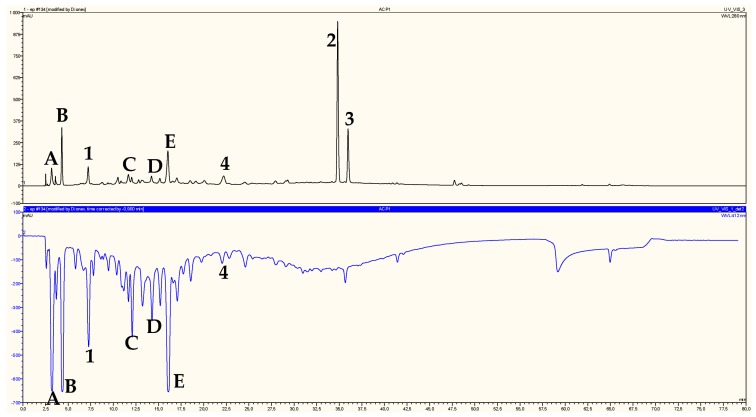
Chromatographic determination of antioxidant compounds (upper chromatogram) and their corresponding antioxidant activity (lower chromatogram) in ethyl acetate extract of *H. ruficomum*. Protocatechuic acid (**1**), *trans*-tiliroside (**2**), *cis-*tiliroside (**3**), astragalin (**4**), unidentified compounds (**A**, **B**, **C**, **D** and **E**).

**Figure 3:**
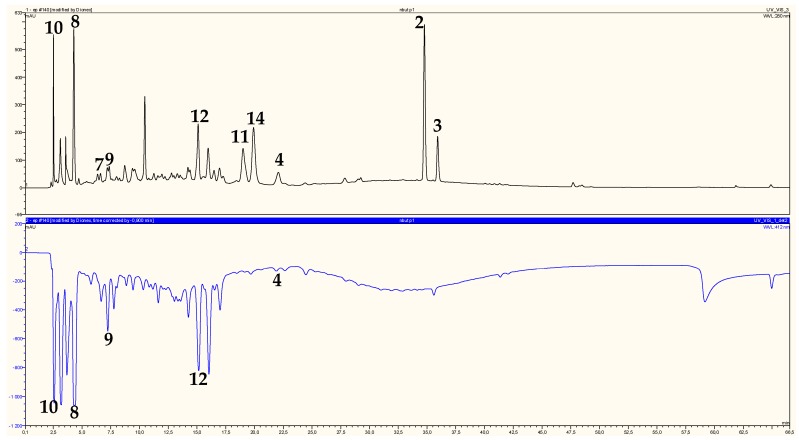
Chromatographic determination of antioxidant compounds (upper chromatogram) and their corresponding antioxidant activity (lower chromatogram) in *n*-butanol extract of *H. ruficomum.* Picein (**7**), vanillic acid 4-*O*-β-d-glucopyranoside (**8**), lavandoside (**9**), 4-hydroxybenzoic acid 4-*O*-β-d-glucopyranoside (**10**), nicotiflorin (**11**), rutin (**12**), narcissin (**14**).

On-line HPLC-ABTS^•+^ profiles of ethyl acetate and *n*-butanol extracts of *H. ruficomum* are reported in Figure 2 and Figure 3, respectively. These profiles showed a wealth of the two extracts in phenolic compounds detected by their absorbancy at 280 nm. The identification of chromatographic peaks was carried out after separation and purification by chromatographic techniques and re-injection of the pure isolated compounds (HPLC-ABTS^•+^) under the same conditions as the extracts. Regarding antioxidant activity, compounds protocatechuic acid (**1**), astragalin (**4**), vanillic acid 4-*O*-β-d-glucopyranoside (**8**), lavandoside (**9**), 4-hydroxybenzoic acid 4-*O*-β-d-glucopyranoside (**10**) and rutin (**12**) showed relatively high radical scavenging capacity (Table 1 and Table 2), in particular vanillic acid 4-*O*-β-d-glucopyranoside (**8**) and 4-hydroxybenzoic acid 4-*O*-β-d-glucopyranoside (**10**) were the most active (833 and 823 mAU respectively) (Table 2). The antioxidant activity of the *n*-BuOH extract is largely due to the presence of the three phenolic acids **8**, **9**, **10** and the flavonoid glycoside rutin (**12**) which represent 88.15% of the total activity (Table 2). The phenolic compound **1** and the flavonoid glucoside **4** represent only 10.34% of the total antioxidant activity of EtOAC extract (Table 1) as the compounds **A**, **B**, **C**, **D** and **E** which also showed high antioxidant activity (Figure 2) could not be isolated in pure state and then were not identified. These five components represented 62.21% of the ethyl acetate extract antioxidant activity (Table 1). *trans*-Tiliroside (**2**), *cis*-tiliroside (**3**), nicotiflorin (**11**) and narcissin (**14**) which showed a relatively high molecular absorbance on the upper chromatogram (Figure 3), exhibited little or no radical scavenging capacities. These results were confirmed off-line by ABTS, ORAC and TEAC assays. The measure of the free radical scavenger capacity of the two studied extracts of *Helianthemum ruficomum* by off-line TEAC test, confirmed these results and showed that EtOAc and *n*-BuOH extracts exhibited comparable activities (TEAC 432 and 431 µMol_TE_/mg, respectively, Table 3). This may be in relation with the strongest amount of phenolic and flavonoid compounds in these extracts. The off-line ABTS assay of the isolated compounds showed also that protocatechuic acid (**1**), vanillic acid 4-*O*-β-d-glucopyranoside (**8**), 4-hydroxybenzoic acid 4-*O*-β-d-glucopyranoside (**10**) and rutin (**12**) were the most potent antioxidant with on-line ABTS assay values: 341, 833, 823 and 594 mAU respectively (Table 1 and Table 2), off-line ABTS values: 202, 75, 254 and 644 mAU respectively (Table 3), ORAC essay values: 690, 651, 435 and 613 µMol_TE_/mg respectively (Table 3) and TEAC assay values: 469, 106, 408 and 556 µMol_TE_/mg (Table 3). The higher activity of rutin (**12**) in comparison to the other flavonoid glycosides present in these extracts may be due to the presence of the ortho di-OH system on the ring B of this molecule [44]. In addition, the higher activity of protocatechuic acid (**1**) in comparison with vanillic acid 4-*O*-β-d-glucopyranoside (**8**) and 4-hydroxybenzoic acid 4-*O*-β-d-glucopyranoside (**10**) may be due the lack of glycosylation, which has been found to diminish the radical scavenging activity [45]. This was also observed for ferulic acid glucoside (**9**), which showed relatively low antioxidant activity in comparison with ferulic acid [46,47].

The presence in this species, of phenolic acids and flavonoids was in good agreement with major studies reported on *Helianthemum* species [14,24,26,27]. The strong accumulation by this species, of *trans*-tiliroside which has been demonstrated to exert multiple biological effects [48,49] and the high antioxidant potential of its extracts and tested compounds, emphasized the possible relevance of this plant for Algerian traditional medicine. Moreover, it is important to note that no report has been published so far on eventual ethnomedical uses of this species. This may be due to a low distribution of this species.

## 3. Materials and Methods

### 3.1. General Procedure

Ultraviolet spectra were recorded using a Shimadzu model UV-1700 spectrophotometer. NMR spectra were obtained by Bruker model Avance 400 and AMX-500 spectrometers (Bruker BioSpin, Rheinstetten, Germany) with standard pulse sequences, operating at 400 and 500 MHz for ^1^H and 100 and 125 MHz for ^13^C, respectively. MeOH-*d*_4_, DMSO-*d*_6_, or CDCl_3_ were used as solvents with TMS as internal standard. High resolution mass spectra (ESI-HRMS) were performed on a Agilent 6520 Accurate Mass Q-TOF (Agilent Corporation, Santa Clara, CA, USA) and a µ-QToF spectrometer (Bruker Daltonics, Wissembourg, France). Column chromatography (CC) was carried out with Si gel Fluka (cat. 60737, 40–63 μm), and column fractions were monitored by TLC Si gel 60 F_254_, 0.2 mm, Macherey Nagel (cat. 818–333) by detection with a spraying reagent (CH_3_CO_2_H/H_2_O/H_2_SO_4_; 80:16:4) followed by heating at 100 °C. Preparative TLC was carried out on Si gel 60 PF_254+366_ (20 cm × 20 cm, 1 mm thickness, Analtech cat. 02014).

### 3.2. Plant Material

The plant material was collected from the area of Mougheul in the south-west of Algeria, latitude: N 32°1′23.6928″ (+32°1′23.6828″), longitude W 2°13′3.0648″ (−2°13′3.0648″) and authenticated by M. Mohamed Benabdelhakem, director of the nature preservation agency, Bechar on the basis of Quezel and Santa [23]. A voucher specimen (HCC0512-MOG-ALG-60) has been deposited at the Herbarium of the VARENBIOMOL research unit, Université des Frères Mentouri Constantine 1.

### 3.3. Extraction and Isolation

Air-dried aerial parts (1448 g) of *Helianthemum ruficomum* (Cistaceae) were macerated at room temperature with EtOH/H_2_O (80:20, *v/v*) for 72 h, three times. After filtration, the filtrates were concentrated in vacuum (up to 35 °C) and dissolved in distilled H_2_O (900 mL) under magnetic stirring and then put at the refrigerator for one night. After filtration, the resulting solution was extracted successively with petroleum ether, CHCl_3_, EtOAc and *n*-BuOH. The organic phases were filtered using common filter paper and concentrated in vacuum up to 35 °C to obtain the following dry extracts: petroleum ether (0.135 g), CHCl_3_ (0.9 g), EtOAc (5.142 g), *n*-BuOH (22.938 g). A part of the EtOAc extract (4 g) was dissolved in 5 mL of MeOH and subjected to column chromatography on silica gel (60–200 mesh, 160 g) eluted with CHCl_3_/MeOH step gradients to yield 41 fractions (F_1_–F_41_) obtained by combining the eluates on the basis of TLC analysis. Fraction F_12_ (57.8 mg) (CHCl_3_/MeOH, 96:4), was submitted to preparative plates of silica gel (CHCl_3_/MeOH, 12:1) to give Protocatechuic acid (**1**) (1 mg). Fraction F_16_ (113.7 mg) (CHCl_3_/MeOH, 94:6), was submitted to preparative plates of silica gel (CHCl_3_/MeOH, 7:1) to give *cis*-tiliroside (1.7 mg). Fraction F_17_ (930.6 mg) (CHCl_3_/MeOH, 93:7), was subjected to preparative plates of silica gel (CHCl_3_/MeOH, 2:0.5) and purified on analytical plates of silica gel GF_254_ (AcOEt/MeOH/H_2_O, 6:1:1) to give *trans*-tiliroside (80 mg) and astragalin (3 mg).

A part of the *n*-buthanol extract (16 g) was dissolved in 15 mL of MeOH and subjected to a column chromatography of silica gel (high-purity grade pore size 60 Å, 70-230 mesh, 63–200 µm, 478 g) eluted with CH_2_Cl_2_/MeOH step gradients to yield 33 fractions (F_1_–F_33_) obtained by combining the eluates on the basic of TLC and analytical HPLC analysis. Fraction F_4_ showed the formation of a white precipitate which was filtered (29.2 mg) and washed with methanol to give stigmasterol and β-sitosterol (5.3 mg) as a mixture (71 and 29%, respectively). From the fractions: F_9_ (288.2 mg), F_11_ (1281.7 mg), F_13_ (2837.3 mg), F_14_ (2054 mg), F_24_ (211 mg) and F_32_ (77 mg), aliquots were dissolved in methanol and submitted to a semi preparative HPLC separation using thermo column hypersil gold C_18_ (5 µm, 250 mm × 10 mm), with a mobile phase delivered at 5 mL/min consisting of mixture of Milli-Q water containing 0.1% formic acid (solvent A) and acetonitrile containing 0.1% formic acid (solvent B). The gradient was as follow: 0 min, 0% B; 30 min, 25% B; maintained during 7 min, to obtain picein (**7**) (2.1 mg) from F_9_, vanillic acid 4-*O*-β-d-glucopyranoside (**8**) (8 mg) and lavandoside (**9**) (1.2 mg) from F_11_, 4-hydroxybenzoic acid 4-*O*-β-d-glucopyranoside (**10**) (1.6 mg) and nicotiflorin (**11**) (2 mg) from F_13_, Rutin (**12**) (70 mg) from F_14_, vicenin-2 (**13**) (1 mg) from F_24_ and narcissin (**14**) (1.2 mg) from F_32_.

### 3.4. Solvents and Chemicals

Solvents: chloroform, ethyl acetate, formic acid and methanol reagent grade were purchased from VWR (Fontenay-sous-bois, France); acetonitrile HPLC grade was purchased from fisher scientific; Milli-Q water (18.2 MΩ) was generated by Millipore synergy system (Molsheim, France).

Chemicals: 2,2′-azino-bis(3-ethylbenzthiazoline-6-sulfonic acid) diammonium salt (ABTS^•+^), (C_18_H_24_N_6_O_6_S_4_) was purchased from Biochemica Applichem (Darmstadt, Germany); 2,2′-azobis(2-methylpropionamidine) dihydrochloride (AAPH), (C_8_H_18_N_6_ 2HCl); (+)-6-hydroxy-2,5,7,8-tetramethylchromane-2-carboxylic acid (trolox), (C_14_H_18_O_4_); fluorescein (C_20_H_12_O_5_), were purchased from Sigma-Aldrich (Steinheim, Germany); sodium chloride was purchased from VWR; potassium chloride and potassium persulfate were purchased from Prolabo (Paris, France); potassium dihydrogen phosphate, disodium hydrogen phosphate and disodium dihydrogen phosphate were purchased from Merck (Darmstadt, Germany).

### 3.5. On-Line HPLC-ABTS^•+^ Assay

The ABTS^•+^ assay was based on the procedure described by Re (1999) [50] and Siddhuraju (2006) [51]. ABTS (7 mM) was dissolved in 20 mL of Milli-Q water, to which potassium persulfate (2.5 mM) was added, generating the radical cation ABTS^•+^ overnight. The solution was left overnight at 4 °C protected from light exposure. This solution was used within 4 days, dilutes in the Phosphate Buffer Saline (PBS) solution (pH 7.4) in order to reach an absorbance of 1.2 at 412 nm. Phosphate Buffer Saline (PBS) was prepared by dissolving in Milli-Q water, 80 g NaCl, 14.4 g Na_2_HPO_4_, 2.4 g KH_2_PO_4_ and 2 g KCl; the volume was completed with Milli-Q water to 1L. The pH was adjusted to 7.4 using NaOH 0.1 mol/L. The radical cation ABTS^•+^ solution (10 mL) was diluted in PBS (50 mL) and completed to 500 mL with Milli-Q water before use. The diode array detector (DAD) was connected to a mixing tee followed by a reaction coil (Peek, 20 m × 0.25 mm) loaded in a temperature controlled oven. Post-column reaction is operated by delivering (0.5 mL/min) the ABTS^•+^ reagent with an Ultimate 3000 variable wavelength detector through the mixing tee. After the reaction coil, the flow pass through a second molecular absorption photometric detector set at 412 nm to detect the reduced form of ABTS^•+^ radical and thus a reduced absorbancy. The result is presented as a double chromatogram, the upper part representing the phenolic compounds, detected by their absorbancy at 280 nm, while the lower part representing the free radical scavenging activity of these phenolic compounds. A negative peak indicates that a compound having radical scavenging activity elutes out of the chromatographic column and react with the ABTS^•+^ radical cation. The area of the chromatographic negative peak gives an indication on the radical-scavenging activity of the considered compound. The column used in the separation of EtOAc and *n*-BuOH extracts, was a Kromasil C_18_ with a 5 µm particle size, 4.60 mm × 250 mm (column temperature: 25 °C). The mobile phase delivered at 1 mL/min, was composed of 0.1% formic acid in H_2_O milli-Q (solvent A) and acetonitrile containing 1% formic acid (solvent B). Gradient was as follow: 0 min, 10% B; 10 min, 20% B; 20 min, 20% B; 50 min, 50% B; 55 min, 50% B; 56 min, 80% B; 66 min, 80% B; 67 min, 10% B, maintained during 13 min. Each phenolic compound was injected into the LC-ABTS^•+^ and quantified by reference to its appropriate authentic standard by absorption at 280 nm, whereas the antioxidant potential was calculated as the concentration of trolox required to produce an equivalent negative peak area by absorption at 412 nm and expressed as trolox equivalent antioxidant capacity (TEAC) or µMol_TE_/mg.

### 3.6. Oxygen Radical Absorbance Capacity (ORAC)

The ORAC assay, developed and validated by Ou et al. (2001) [52], was performed as described by Davalos (2004) [53] with minor modification Volden (2008) [54]. The assay measures the oxidative degradation of fluorescein by peroxyl radicals initiated by 2′,2-azobis(2-methylpropionamidine) dihydrochloride (AAPH) at 37 °C. Free radical scavenging molecules protect fluorescein from the oxidative degradation and until exhaustion, slow reduction of the fluorescence signal by inducing latency. The area under the curve of the kinetics of fluorescence is directly proportional to the amount and effectiveness of the free radical scavengers present in a sample. The results are therefore expressed as trolox equivalent (µMol_TE_/mg) of dry extract. The products are dissolved in a mixture of water/methanol (70/30) at 1 mg/mL (1000 ppm), and then have to be diluted with water (between 25 and 500 ppm) before being placed in triplicate in 96 wells micro-plate up to 10 µL/well. A trolox standard range between 25 and 500 µMol/L was also filed in triplicate. An aqueous solution of 150 µL fluorescein (8.5 × 10^−6^ mol/L) was added per well. An automatic dispenser then permits the initiation of the reaction by the addition of AAPH (30 µL, 153 µMol/L) to each well from the initiation of the generation of radicals by the addition of AAPH, the intensity of fluorescence emitted is measured every 5 min for 2 h with a wavelength of excitation between 400 and 600 nm.

## 4. Conclusions

The present study allowed the isolation, structural elucidation and antioxidant evaluation of phenolics and flavonoids from *Helianthemum ruficomum*, an endemic Saharan species on which no report is available so far. In this work, 14 compounds were isolated and identified from the ethyl acetate and *n*-butanol soluble parts of the aqueous EtOH extract, five phenolics: protocatechuic acid (**1**), picein (**7**), vanillic acid 4-*O*-β-d-glucopyranoside (**8**), lavandoside (**9**), 4-hydroxybenzoic acid 4-*O*-β-d-glucopyranoside (**10**); seven flavonoid glycosides: *trans*-tiliroside (**2**), slightly contamined by its stereoisomer *cis*-tiliroside, *cis*-tiliroside (**3**) contamined by *trans*-tiliroside, astragalin (**4**), nicotiflorin (**11**), rutin (**12**), vicenin-*2* (**13**), narcissin (**14**); and a mixture (71–29%) of stigmasterol (**5**) and β-sitosterol (**6**) respectively. All the compounds were identified by spectral analysis, mainly ESI-HRMS, UV and NMR experiments (^1^H, ^13^C, DEPT, DOSY, COSY, NOESY, HSQC and HMBC) and comparison of their spectroscopic data with those reported in the literature. Compounds **5**, **7**, **8**, **9**, **10** and **14** were new for the genus *Helianthemum*. The investigated extracts and isolated compounds were evaluated for their free radical scavenging capacity by on-line HPLC-ABTS^•+^ screening. The antioxidant properties were confirmed by ORAC and TEAC assays. The results clearly indicated high antioxidant potential of the extracts and tested compounds of this species and agreed with literature data that free radical scavenging activity depends on the molecular structure, the number and position of the hydroxyl groups of tested compounds. Moreover, given the large amounts isolated and purified in this work, of *trans*-tiliroside and rutin which besides its numerous recognized biological activities, is used as oral complement; it becomes important to note that this *Helianthemum* species might be developed industrially for its rich content of these bioactive components. For this reason, this plant could be a good candidate for culture as a crop.

## Figures and Tables

**Table 1:** Free radical scavenging activities of the phenolic compounds in the EtOAc extract of *H. ruficomum* based on ABTS^•+^ and their participation (%) in total antioxidant capacities with ABTS assay.

Peaks	Compounds	ABTS (µg_TE_/mL)	Radical-Scavenging Activity (mAU)	Scavenging Activity Percent (%)
**A**	Not identified	131.83	546	17.40
**B**	Not identified	137.47	569	16.86
**1**	Protocatechuic acid	81.56	341	8.37
**C**	Not identified	63.96	269	5.48
**D**	Not identified	42.57	181	3.97
**E**	Not identified	118.05	490	18.50
**4**	Astragalin	11.48	54	1.97

**Table 2:** Free radical scavenging activities of the phenolic compounds in *n*-butanol extract of *H. ruficomum*, based on ABTS^•+^ and their participation (%) in total antioxidant capacities with ABTS assay.

Peaks	Compounds	ABTS (µg_TE_/mL)	Radical-Scavenging Activity (mAU)	Scavenging Activity Percent (%)
**8**	Vanillic acid 4-*O*-β-d-glucopyranoside	201.91	833	35.27
**9**	Lavandoside	81.66	341	9.37
**10**	4-hydroxybenzoic acid 4-*O*-β-d-glucopyranoside	199.50	823	20.31
**12**	Rutin	143.59	594	23.20

**Table 3:** Free radical scavenging activities of the pure compounds and extracts of *H. ruficomum* based on ABTS, Oxygen Radical Absorbance Capacity (ORAC) and trolox equivalent antioxidant capacity (TEAC) assays.

Peaks	Pure Compounds or Extracts	ABTS (µg_TE_/mL)	Radical-Scavenging Activity (mAU)	ORAC (µMol_TE_/mg)	TEAC (µMol_TE_/mg)
	EtOAc extract	-	-	18.43 ± 12.58	432.06 ± 15.84
	*n*-BuOH extract	-	-	5.64 ± 0.21	430.66 ± 80.25
**1**	Protocatechuic acid	47.59	202	690.40 ± 45.67	469.48 ± 15.71
**4**	Astragalin	1.48	13	300.006 ± 82.71	144.69 ± 15.36
**8**	Vanillic acid 4-*O*-β-d-glucopyranoside	16.49	75	650.91 ± 185.18	105.56 ± 8.95
**9**	Lavandoside	91.49	381	37.44 ± 24.74	12.14 ± 7.34
**10**	4-Hydroxybenzoic acid 4-*O*-β-d-glucopyranoside	60.45	254	434.86 ± 36.43	407.94 ± 32.73
**12**	Rutin	155.66	644	612.77 ± 165.45	555.66 ± 20.79

(Results expressed as mean ± SD, *n* = 3).

## Supplementary Materials

^1^H-NMR, ^13^C-NMR, DEPT, COSY, HSQC, HMBC, NOESY, and MS spectra are available as supplementary materials. Supplementary materials can be accessed at: http://www.mdpi.com/1420-3049/22/2/239/s1.
