# Supplementary material for: On-Line Screening, Isolation and Identification of Antioxidant Compounds of Helianthemum ruficomum
Source: Molecules. 2017 Feb 8;22(2):239. doi: 10.3390/molecules22020239 (PMC6155680; doi:10.3390/molecules22020239)
Supplement: Supplementary file 1 [file molecules-22-00239-s001.pdf]

Supplementary Materials: On-Line Screening, Isolation and Identification of Antioxidant Compounds of *Helianthemum ruficomum*

Yasmine Chemam, Samir Benayache, Eric Marchioni, Minjie Zhao, Paul Mosset and Fadila Benayache

List of Figures

**Figure S1:**	ESI-HRMS(+) of Protocatechuic acid	S4
**Figure S2:**	^{1}H NMR spectrum (400 MHz, CD_3OD, δppm) of protocatechuic acid	S5
**Figure S3:**	HSQC spectrum (400 MHz, CD_3OD, δppm) of protocatechuic acid	S5
**Figure S4:**	HMBC spectrum (400 MHz, CD_3OD, δppm) of protocatechuic acid	S6
**Figure S5:**	^{13}C NMR spectrum (100 MHz, CD_3OD, δppm) of protocatechuic acid	S6
**Figure S6:**	ESI-HRMS(+) of *trans*-tiliroside	S7
**Figure S7:**	^{1}H NMR spectrum (400 MHz, DMSO-d_6, δppm) of *trans*-tiliroside	S8
**Figure S8:**	HSQC spectrum (Spreading out 1) (500 MHz, DMSO-d_6, δppm) of *trans*-tiliroside	S8
**Figure S9:**	HSQC spectrum (Spreading out 2) (500 MHz, DMSO-d_6, δppm) of *trans*-tiliroside	S9
**Figure S10:**	COSY spectrum (Spreading out 1) (500 MHz, DMSO-d_6, δppm) of *trans*-tiliroside	S9
**Figure S11:**	COSY spectrum (Spreading out 2) (500 MHz, DMSO-d_6, δppm) of *trans*-tiliroside	S10
**Figure S12:**	HMBC spectrum (Spreading out 1) (500 MHz, DMSO-d_6, δppm) of *trans*-tiliroside	S10
**Figure S13:**	HMBC spectrum (Spreading out 2) (500 MHz, DMSO-d_6, δppm) of *trans*-tiliroside	S11
**Figure S14:**	HMBC spectrum (Spreading out 3) (500 MHz, DMSO-d_6, δppm) of *trans*-tiliroside	S11
**Figure S15:**	HMBC spectrum (Spreading out 4) (500 MHz, DMSO-d_6, δppm) of *trans*-tiliroside	S12
**Figure S16:**	HMBC spectrum (Spreading out 5) (500 MHz, DMSO-d_6, δppm) of *trans*-tiliroside	S12
**Figure S17:**	HMBC spectrum (Spreading out 6) (500 MHz, DMSO-d_6, δppm) of *trans*-tliroside	S13
**Figure S18:**	^{1}H NMR spectrum (400 MHz, DMSO-d_6, δppm) of *trans*-tiliroside	S13
**Figure S19:**	^{13}C NMR spectrum (Spreading out 1) (125 MHz, DMSO-d_6, δppm) of *trans*-tiliroside	S14
**Figure S20:**	^{13}C NMR spectrum (Spreading out 2) (125 MHz, DMSO-d_6, δppm) of *trans*-tiliroside	S14
**Figure S21:**	^{13}C NMR spectrum (Spreading out 3) (125 MHz, DMSO-d_6, δppm) of *trans*-tiliroside	S15
**Figure S22:**	^{13}C NMR spectrum (Spreading out 4) (125 MHz, DMSO-d_6, δppm) of *trans*-tiliroside	S15
**Figure S23:**	ESI-HRMS(+) of *cis*-tiliroside	S16
**Figure S24:**	^{1}H NMR spectrum (Spreading out 1) (500 MHz, CD_3OD, δppm) of *cis*-tiliroside	S16
**Figure S25:**	^{1}H NMR spectrum (Spreading out 2) (500 MHz, CD_3OD, δppm) of *cis*-tiliroside	S17
**Figure S26:**	HSQC NMR spectrum (500 MHz, CD_3OD, δppm) of *cis*-tiliroside	S17
**Figure S27:**	ESI-HRMS(+) of astragalin	S18
**Figure S28:**	^{1}H NMR spectrum (400 MHz, CD_3OD, δppm) of astragalin	S18
**Figure S29:**	HSQC spectrum (400 MHz, CD_3OD, δppm) of astragalin	S19
**Figure S30:**	COSY spectrum (400 MHz, CD_3OD, δppm) of astragalin	S19
**Figure S31:**	HSQC spectrum (400 MHz, CD_3OD, δppm) of astragalin	S20
**Figure S32:**	HSQC spectrum (Spreading out 1) (400 MHz, CD_3OD, δppm) of astragalin	S20
**Figure S33:**	HMBC spectrum (Spreading out 1) (400 MHz, CD_3OD, δppm) of astragalin	S21
**Figure S34:**	^{13}C spectrum (100 MHz, CD_3OD, δppm) of astragalin	S21
**Figure S35:**	ESI-HRMS(+) of picein	S22
**Figure S36:**	^{1}H NMR spectrum (Spreading out 1) (400 MHz, DMSO-d_6, δppm) of picein	S23
**Figure S37:**	HSQC NMR spectrum (400 MHz, DMSO-d_6, δppm) of picein	S23
**Figure S38:**	COSY NMR spectrum (400 MHz, DMSO-d_6, δppm) of picein	S24
**Figure S39:**	HSQC NMR spectrum (Spreading out 1) (400 MHz, DMSO-d_6, δppm) of picein	S24
**Figure S40:**	HMBC NMR spectrum (400 MHz, DMSO-d_6, δppm) of picein	S25
**Figure S41:**	HSQC NMR spectrum (Spreading out 2) (400 MHz, DMSO-d_6, δppm) of picein	S25
**Figure S42:**	^{13}C NMR spectrum (Spreading out 1) (100 MHz, DMSO-d_6, δppm) of picein	S26
**Figure S43:**	ESI-HRMS(+) of Vanillic acid 4-*O*-β-D-glucopyranoside	S26
**Figure S44:**	^{1}H NMR spectrum (400 MHz, CD_3OD, δppm) of Vanillic acid 4-*O*-β-D-glucopyranoside	S27

Figure S45: HSQC NMR spectrum (400 MHz, CD_3OD, δppm) of Vanillic acid 4-*O*-β-D-glucopyranoside S27
Figure S46: COSY NMR spectrum (Spreading out 1) (400 MHz, CD_3OD, δppm) of Vanillic acid 4-*O*-β-D-glucopyranoside S28
Figure S47: HSQC NMR spectrum (Spreading out 1) (400 MHz, CD_3OD, δppm) of Vanillic acid 4-*O*-β-D-glucopyranoside S28
Figure S48: HMBC NMR spectrum (Spreading out 1) (400 MHz, CD_3OD, δppm) of Vanillic acid 4-*O*-β-D-glucopyranoside S29
Figure S49: HSQC NMR spectrum (Spreading out 2) (400 MHz, CD_3OD, δppm) of Vanillic acid 4-*O*-β-D-glucopyranoside S29
Figure S50: ^{13}C NMR spectrum (100 MHz, CD_3OD, δppm) of Vanillic acid 4-*O*-β-D-glucopyranoside S30
Figure S51: ESI-HRMS(+) of Lavandoside S30
Figure S52: ^{1}H NMR spectrum (400 MHz, CD_3OD, δppm) of Lavandoside S31
Figure S53: ^{1}H NMR spectrum (Spreading out 1) (400 MHz, CD_3OD, δppm) of Lavandoside S31
Figure S54: HSQC NMR spectrum (400 MHz, CD_3OD, δppm) of Lavandoside S32
Figure S55: COSY NMR spectrum (400 MHz, CD_3OD, δppm) of Lavandoside S32
Figure S56: HSQC NMR spectrum (Spreading out 1) (400 MHz, CD_3OD, δppm) of Lavandoside S33
Figure S57: HMBC NMR spectrum (Spreading out 1) (400 MHz, CD_3OD, δppm) of Lavandoside S33
Figure S58: HMBC NMR spectrum (Spreading out 2) (400 MHz, CD_3OD, δppm) of Lavandoside S34
Figure S59: HMBC NMR spectrum (Spreading out 3) (400 MHz, CD_3OD, δppm) of Lavandoside S34
Figure S60: HSQC NMR spectrum (Spreading out 2) (400 MHz, CD_3OD, δppm) of Lavandoside S35
Figure S61: ^{13}C NMR spectrum (100 MHz, CD_3OD, δppm) of Lavandoside S36
Figure S62: ESI-HRMS (−) of 4-Hydroxy*benzoic acid* 4-*O*-β-D-glucopyranoside S36
Figure S63: ^{1}H NMR spectrum (500 MHz, CD_3OD, δppm) of 4-Hydroxy*benzoic acid* 4-*O*-β-D-glucopyrnoside S37
Figure S64: HSQC NMR spectrum (500 MHz, CD_3OD, δppm) of 4-Hydroxy*benzoic acid* 4-*O*-β-D-glucopyranoside S37
Figure S65: COSY NMR spectrum (Spreading out 1) (500 MHz, CD_3OD, δppm) of 4-Hydroxy*benzoic acid* 4-*O*-β-D-glucopyranoside S38
Figure S66: HSQC NMR spectrum (Spreading out 1) (500 MHz, CD_3OD, δppm) of 4-Hydroxy*benzoic acid* 4-*O*-β-D-glucopyranoside S38
Figure S67: HMBCNMR spectrum (Spreading out 1) (500 MHz, CD_3OD, δppm) of 4-Hydroxy*benzoic acid* 4-*O*-β-D-glucopyranoside S39
Figure S68: HSQC NMR spectrum (Spreading out 2) (500 MHz, CD_3OD, δppm) of 4-Hydroxy*benzoic acid* 4-*O*-β-D-glucopyranoside S39
Figure S69: HSQC NMR spectrum (125 MHz, CD_3OD, δppm) of 4-Hydroxy*benzoic acid* 4-O-β-D-glucopyranoside S40
Figure S70: ESI-HRMS(+) of Nicotiflorin S40
Figure S71: ^{1}H NMR spectrum (400 MHz, DMSO-d_6, δppm) of Nicotiflorin S41
Figure S72: HSQC NMR spectrum (Spreading out 1) (400 MHz, DMSO-d_6, δppm) of Nicotiflorin S41
Figure S73: COSY NMR spectrum (Spreading out 1) (400 MHz, DMSO-d_6, δppm) of Nicotiflorin S42
Figure S74: HSQC NMR spectrum (Spreading out 2) (400 MHz, DMSO-d_6, δppm) of Nicotiflorin S42
Figure S75: HSQC NMR spectrum (Spreading out 3) (400 MHz, DMSO-d_6, δppm) of Nicotiflorin S43
Figure S76: COSY NMR spectrum (Spreading out 2) (400 MHz, DMSO-d_6, δppm) of Nicotiflorin S43
Figure S77: HSQC NMR spectrum (Spreading out 4) (400 MHz, DMSO-d_6, δppm) of Nicotiflorin S44
Figure S78: HMBC NMR spectrum (400 MHz, DMSO-d_6, δppm) of Nicotiflorin S44
Figure S79: HSQC NMR spectrum (Spreading out 5) (400 MHz, DMSO-d_6, δppm) of Nicotiflorin S45
Figure S80: ^{13}C NMR spectrum (Spreading out 1) (100 MHz, DMSO-d_6, δppm) of Nicotiflorin S45
Figure S81: ^{13}C NMR spectrum (100 MHz, DMSO-d_6, δppm) of Nicotiflorin S46
Figure S82: ESI-HRMS(+) of rutin S46
Figure S83: ^{1}H NMR spectrum (400 MHz, CD_3OD, δppm) of rutin S47
Figure S84: HSQC NMR spectrum (Spreading out 1) (400 MHz, CD_3OD, δppm) of rutin S47
Figure S85: COSY NMR spectrum (Spreading out 1) (400 MHz, CD_3OD, δppm) of rutin S48
Figure S86: HSQC NMR spectrum (Spreading out 2) (400 MHz, CD_3OD, δppm) of rutin S48
Figure S87: HSQC NMR spectrum (Spreading out 3) (400 MHz, CD_3OD, δppm) of rutin S49
Figure S88: COSY NMR spectrum (Spreading out 2) (400 MHz, CD_3OD, δppm) of rutin S49
Figure S89: HMBC NMR spectrum (Spreading out 1) (400 MHz, CD_3OD, δppm) of rutin S50
Figure S90: HMBC NMR spectrum (Spreading out 2) (400 MHz, CD_3OD, δppm) of rutin S50
Figure S91: HMBC NMR spectrum (Spreading out 4) (400 MHz, CD_3OD, δppm) of rutin S51
Figure S92: ^{13}CNMR spectrum (100 MHz, CD_3OD, δppm) of rutin S51
Figure S93: ESI-HRMS(+) of vicenin-2 S52
Figure S94: ^{1}H NMR spectrum (400 MHz, DMSO-d_6, δppm) of vicenin-2 S52
Figure S95: HSQC NMR spectrum (Spreading out 1) (400 MHz, DMSO-d_6, δppm) of vicenin-2 S53
Figure S96: COSY NMR spectrum (Spreading out 1) (400 MHz, DMSO-d_6, δppm) of vicenin-2 S53
Figure S97: HSQC NMR spectrum (Spreading out 2) (400 MHz, DMSO-d_6, δppm) of vicenin-2 S54
Figure S98: COSY NMR spectrum (Spreading out 2) (400 MHz, DMSO-d_6, δppm) of vicenin-2 S54

Figure S99: ^{13}C NMR DEPT spectrum (100 MHz, DMSO-d_6, δppm) of vicenin-2 S55

Figure S100: ^{13}C NMR spectrum (100 MHz, DMSO-d_6, δppm) of vicenin-2 S55

Figure S101: HSQC NMR spectrum (Spreading out 3) (400 MHz, DMSO-d_6, δppm) of vicenin-2 S56

Figure S102: HMBC NMR spectrum (Spreading out 1) (400 MHz, DMSO-d_6, δppm) of vicenin-2 S56

Figure S103: HSQC NMR spectrum (Spreading out 4) (400 MHz, DMSO-d_6, δppm) of vicenin-2 S57

Figure S104: ESI-HRMS(+) of narcissin S58

Figure S105: ^{1}H NMR spectrum (400 MHz, CD_3OD, δppm) of narcissin S58

Figure S106: HSQC NMR spectrum (Spreading out 1) (400 MHz, CD_3OD, δppm) of narcissin S59

Figure S107: COSY NMR spectrum (Spreading out 1) (400 MHz, CD_3OD, δppm) of narcissin S59

Figure S108: ^{13}C NMR spectrum (Spreading out 1) (100 MHz, CD_3OD, δppm) of narcissin S60

Figure S109: HSQC NMR spectrum (Spreading out 2) (400 MHz, CD_3OD, δppm) of narcissin S60

Figure S110: HSQC NMR spectrum (Spreading out 3) (400 MHz, CD_3OD, δppm) of narcissin S61

Figure S111: COSY NMR spectrum (Spreading out 2) (400 MHz, CD_3OD, δppm) of narcissin S61

Figure S112: ^{13}C NMR spectrum (Spreading out 2) (100 MHz, CD_3OD, δppm) of narcissin S62

Figure S113: HSQC NMR spectrum (Spreading out 4) (400 MHz, CD_3OD, δppm) of narcissi S62

Figure S114: HMBC NMR spectrum (Spreading out 1) (400 MHz, CD_3OD, δppm) of narcissin S63

Figure S115: HSQC NMR spectrum (Spreading out 5) (400 MHz, CD_3OD, δppm) of narcissin S63

Figure S116: ^{13}C NMR spectrum (Spreading out 3) (100 MHz, CD_3OD, δppm) of narcissin S64

Figure S117: ^{13}C NMR spectrum (Spreading out 4) (100 MHz, CD_3OD, δppm) of narcissin S64

Molecule 1: Protocatechuic acid

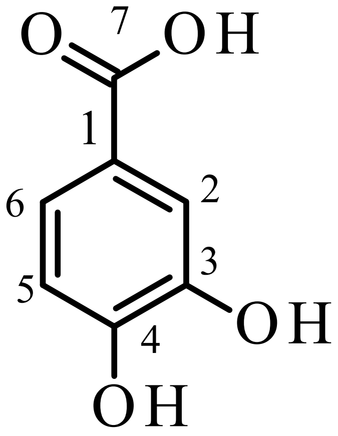

1

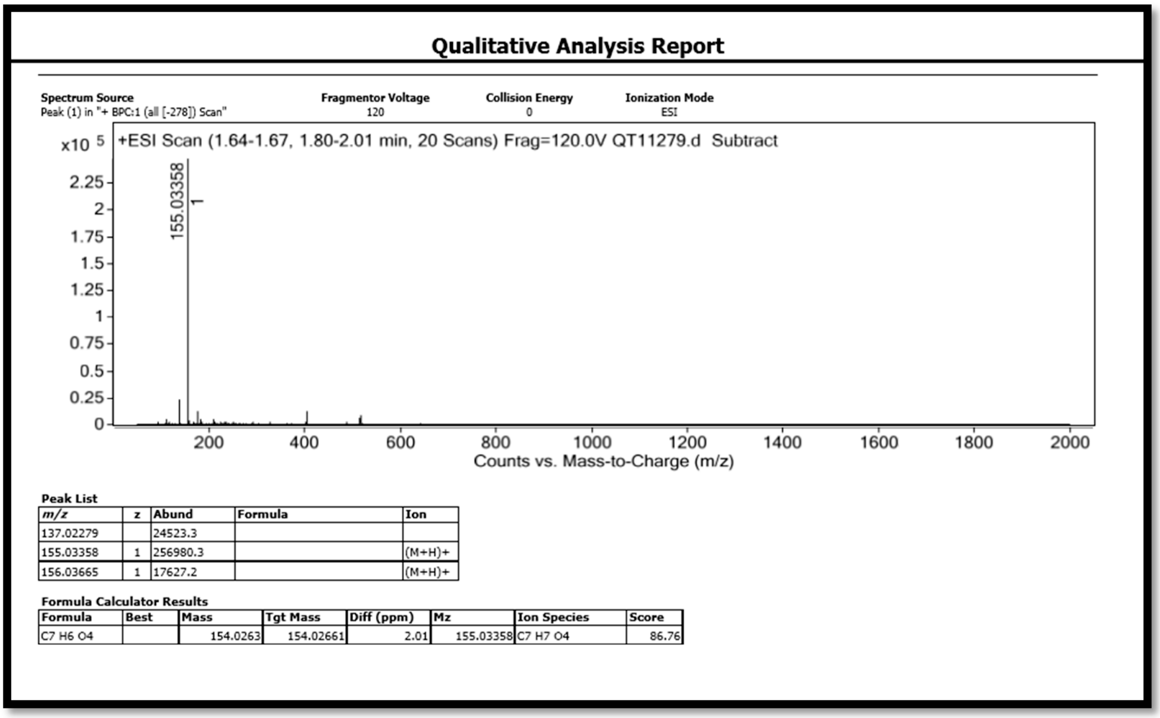

Qualitative Analysis Report

Spectrum Source	Fragmentor Voltage	Collision Energy	Ionization Mode
Peak (1) in "+ BPC:1 (all [-278]) Scan"	120	0	ESI

Peak List

m/z	z	Abund	Formula	Ion
137.02279		24523.3		
155.03358	1	256980.3		(M+H)+
156.03665	1	17627.2		(M+H)+

Formula Calculator Results

Formula	Best	Mass	Tgt Mass	Diff (ppm)	Mz	Ion Species	Score
C7 H6 O4		154.0263	154.02661	2.01	155.03358	C7 H7 O4	86.76

Figure S1. ESI-HRMS(+) of Protocatechuic acid.

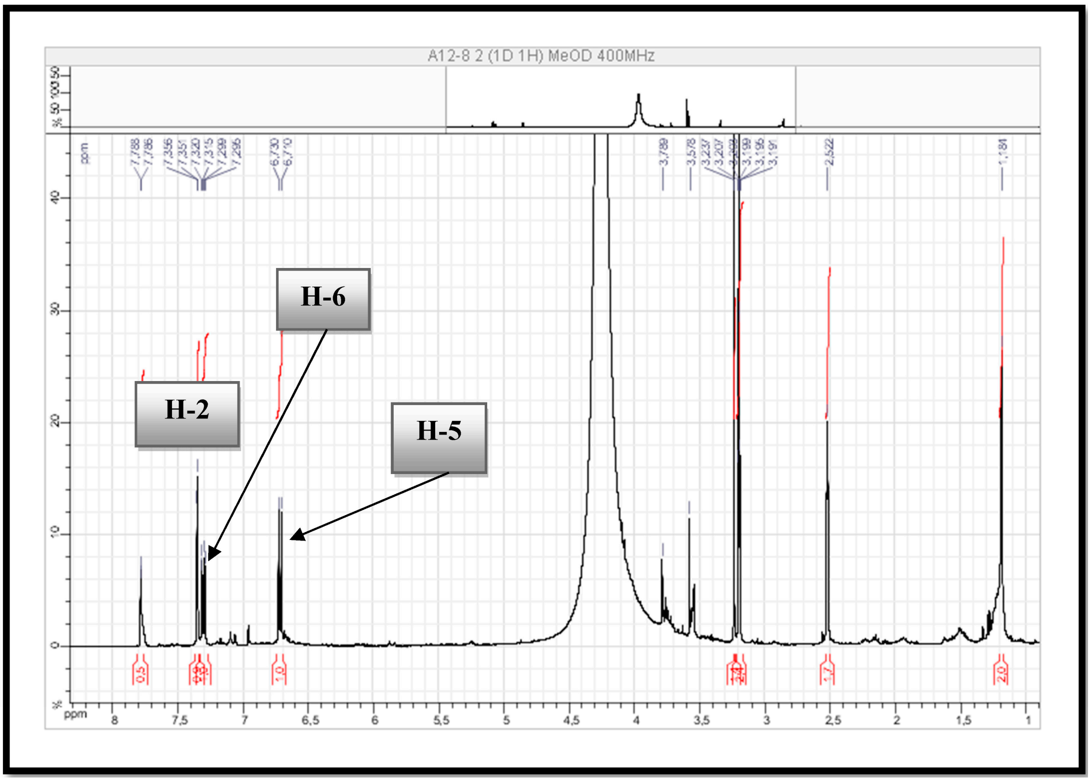

Figure S2. ^{1}H NMR spectrum (400 MHz, CD_3OD, δppm) of protocatechuic acid.

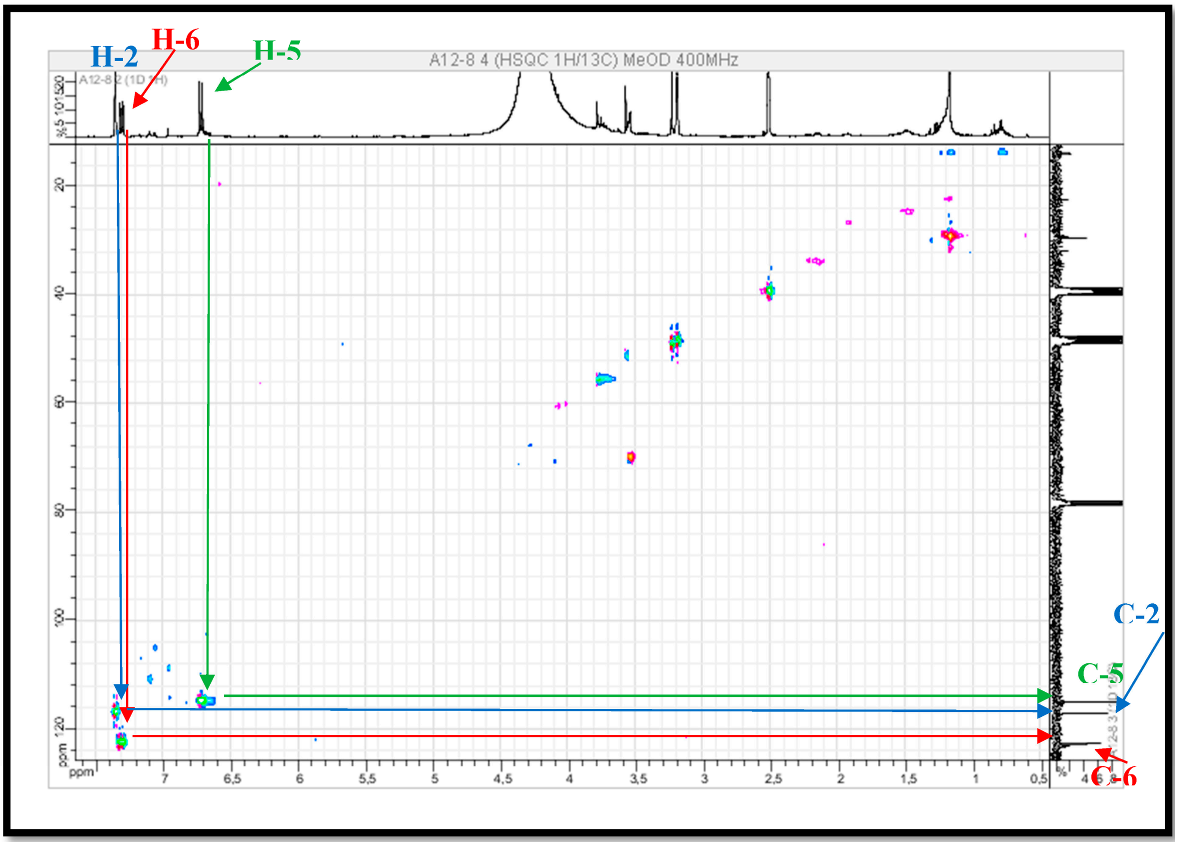

Figure S3. HSQC spectrum (400 MHz, CD_3OD, δppm) of protocatechuic acid.

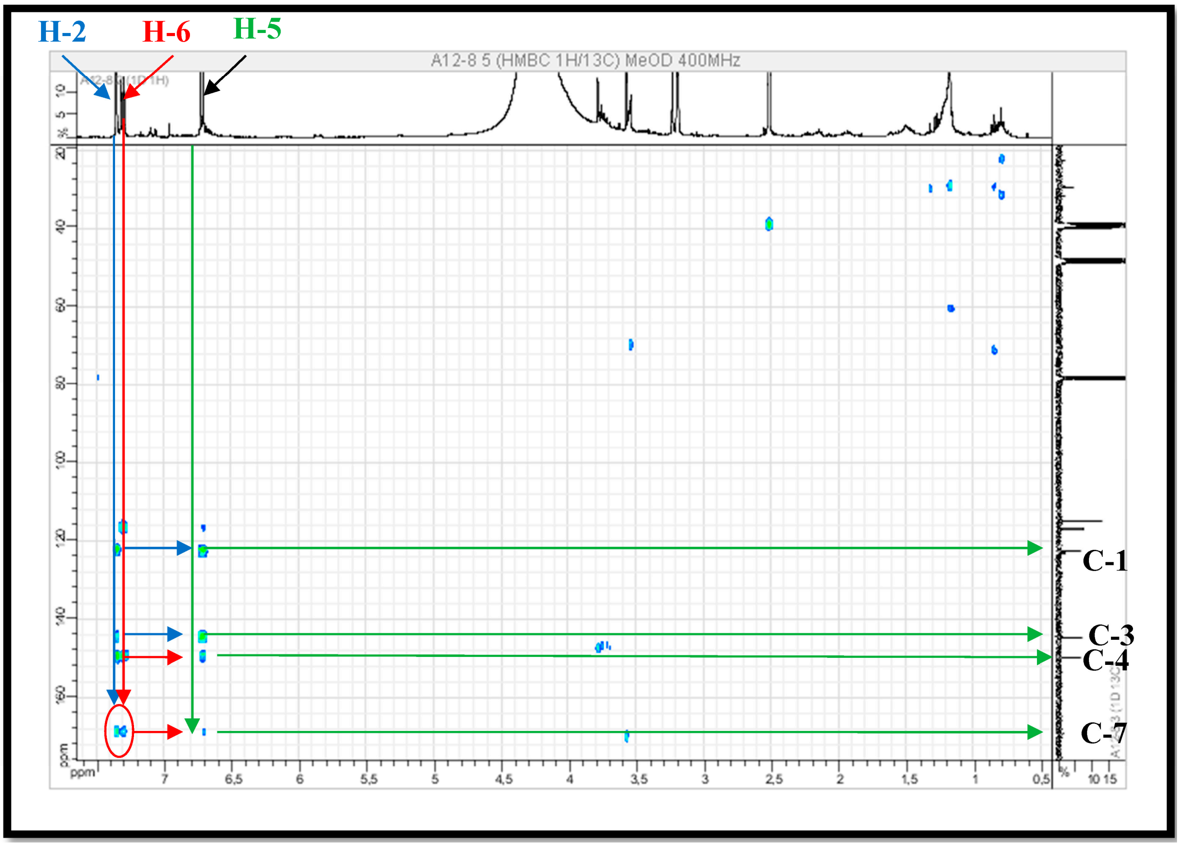

Figure S4. HMBC spectrum (400 MHz, CD_3OD, δppm) of protocatechuic acid.

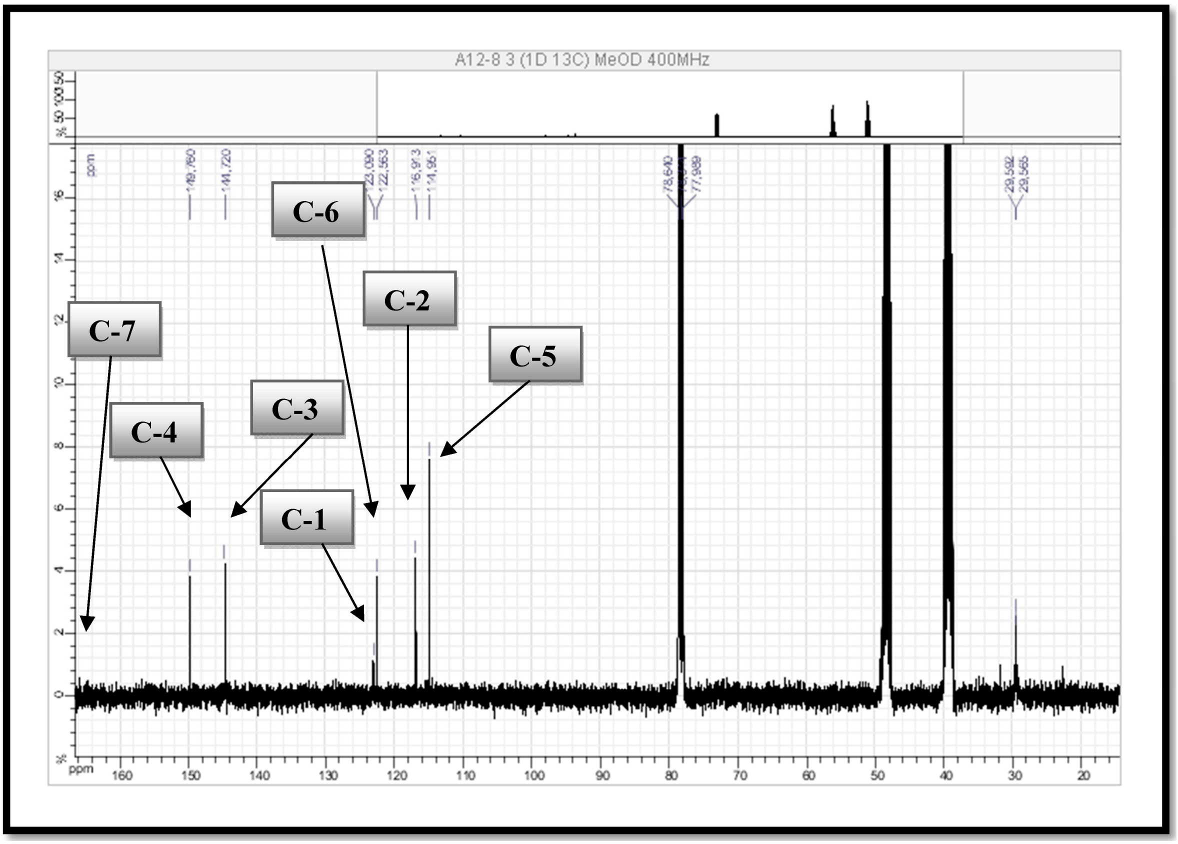

Figure S5. ^{13}C NMR spectrum (100 MHz, CD_3OD, δppm) of protocatechuic acid.

Molecule 2: *Trans*-tiliroside

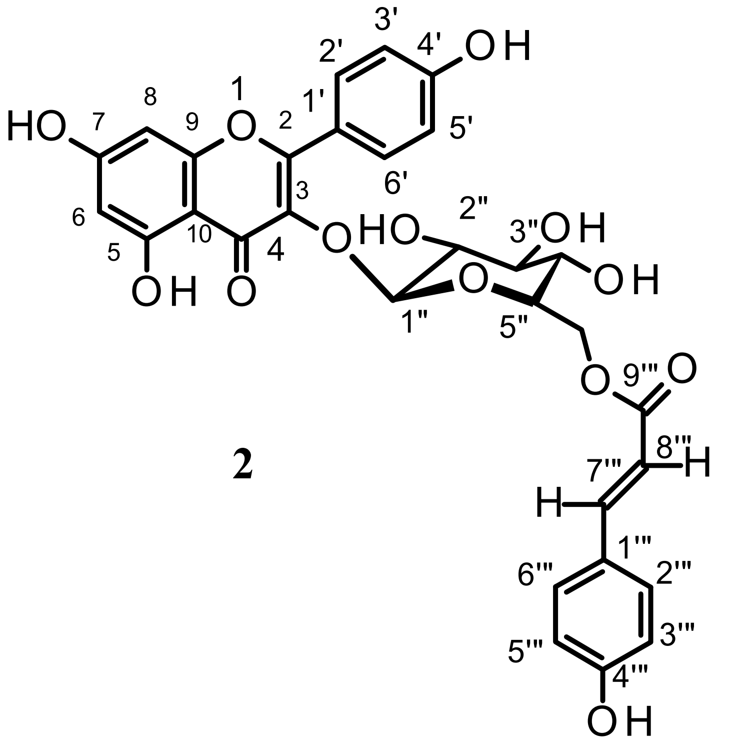

2

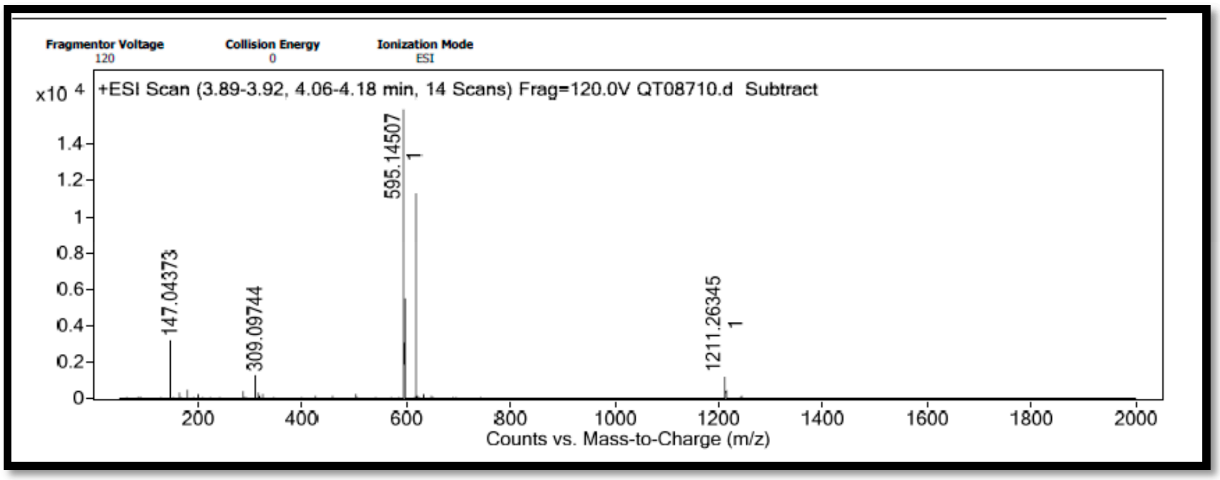

Figure S6. ESI-HRMS(+) of *trans*-tiliroside.

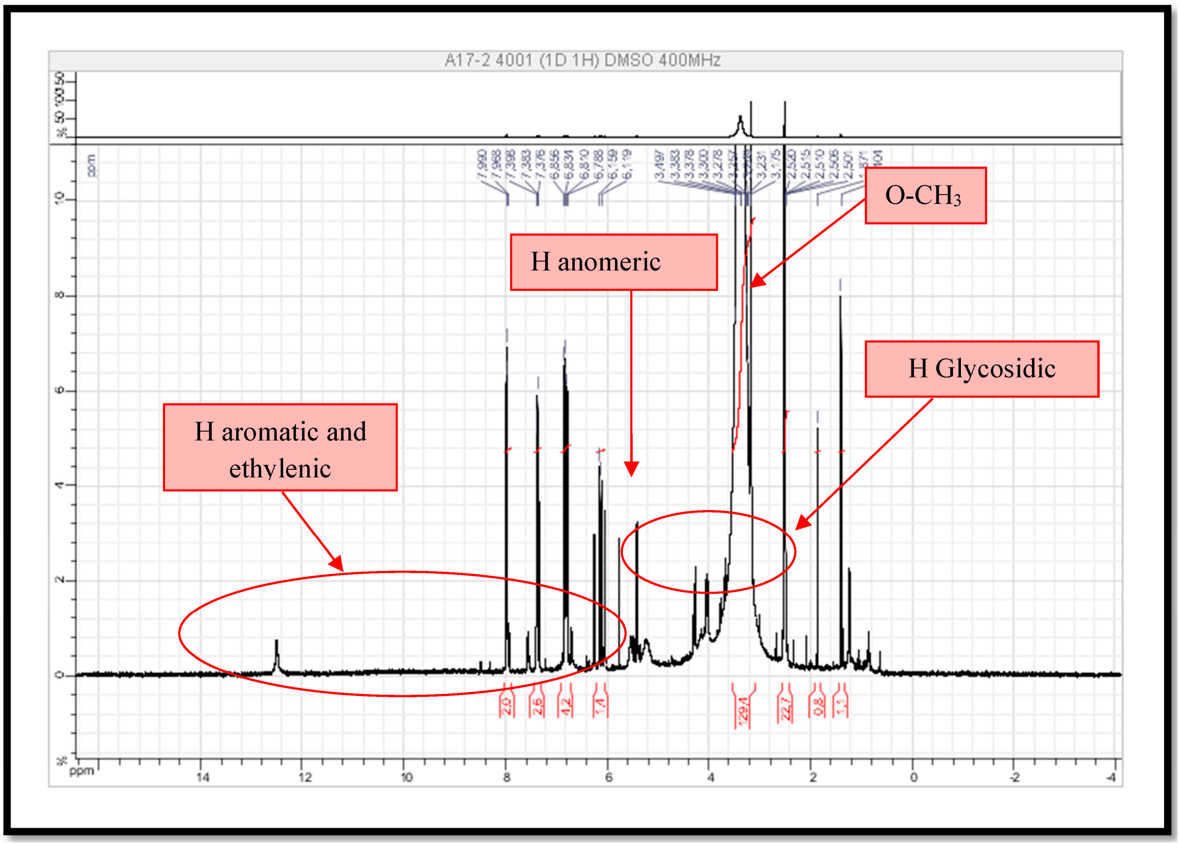

Figure S7. ^{1}H NMR spectrum (400 MHz, DMSO-d_6, δppm) of *trans*-tiliroside.

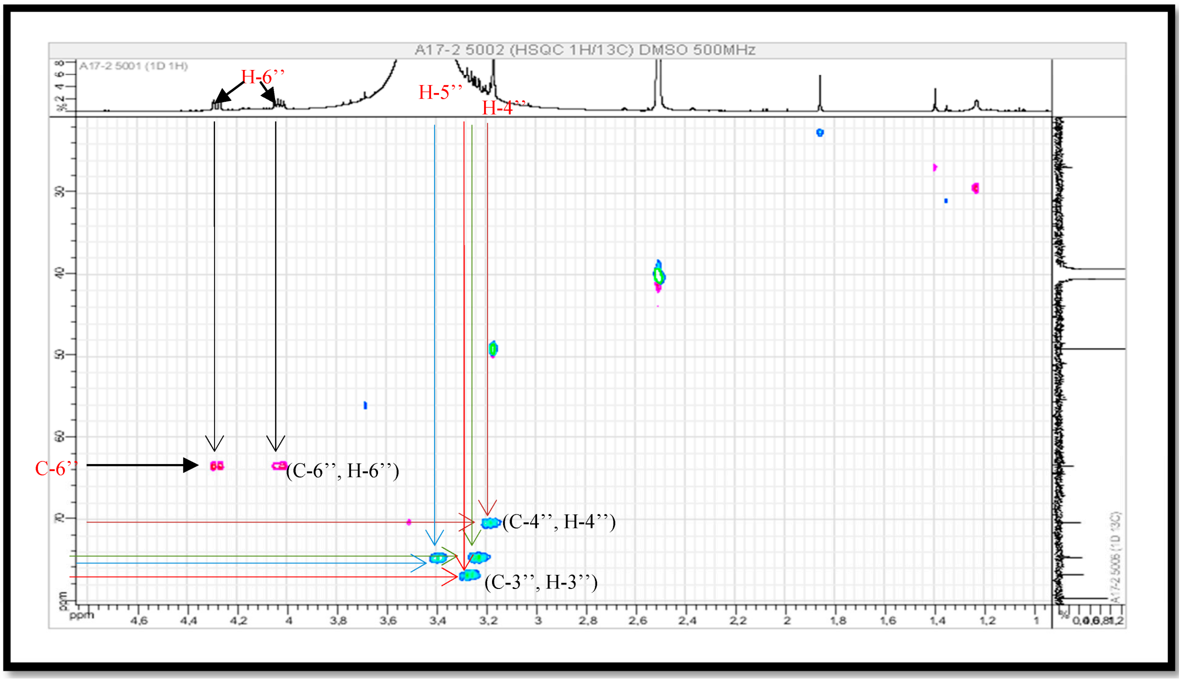

Figure S8. HSQC spectrum (spreading out 1) (500 MHz, DMSO-d_6, δppm) of *trans*-tiliroside.

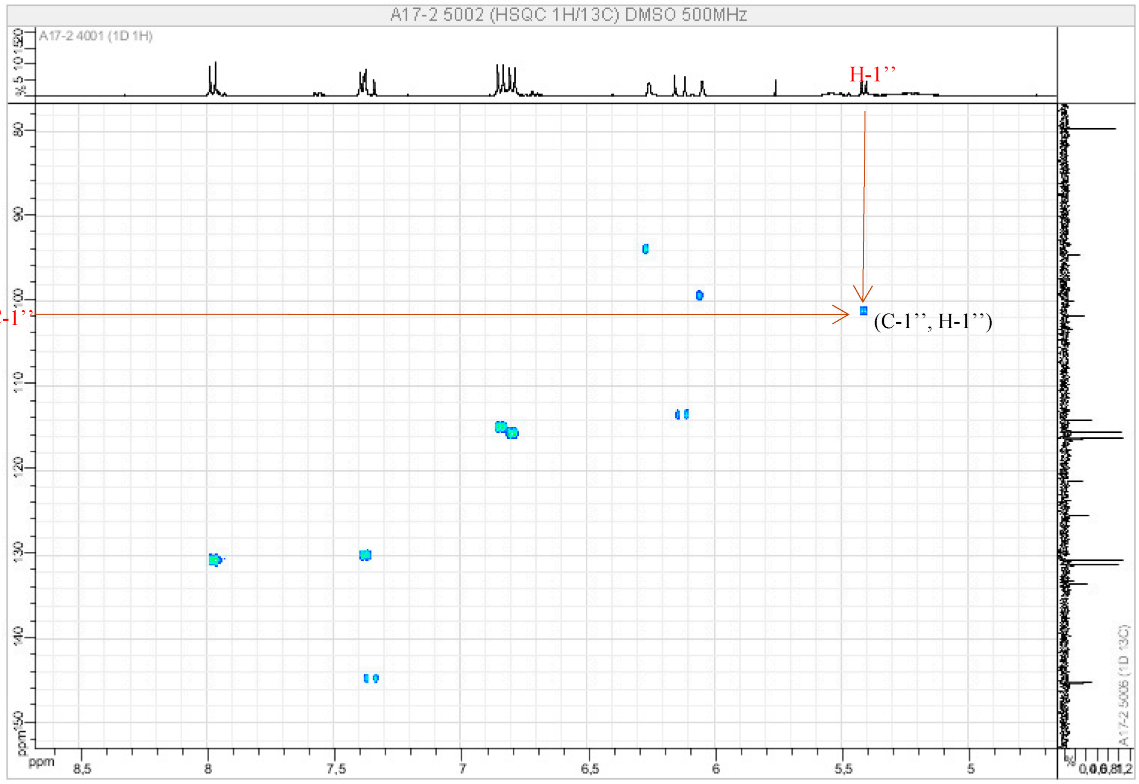

Figure S9. HSQC spectrum (spreading out 2) (500 MHz, DMSO-d_6, δppm) of *trans*-tiliroside.

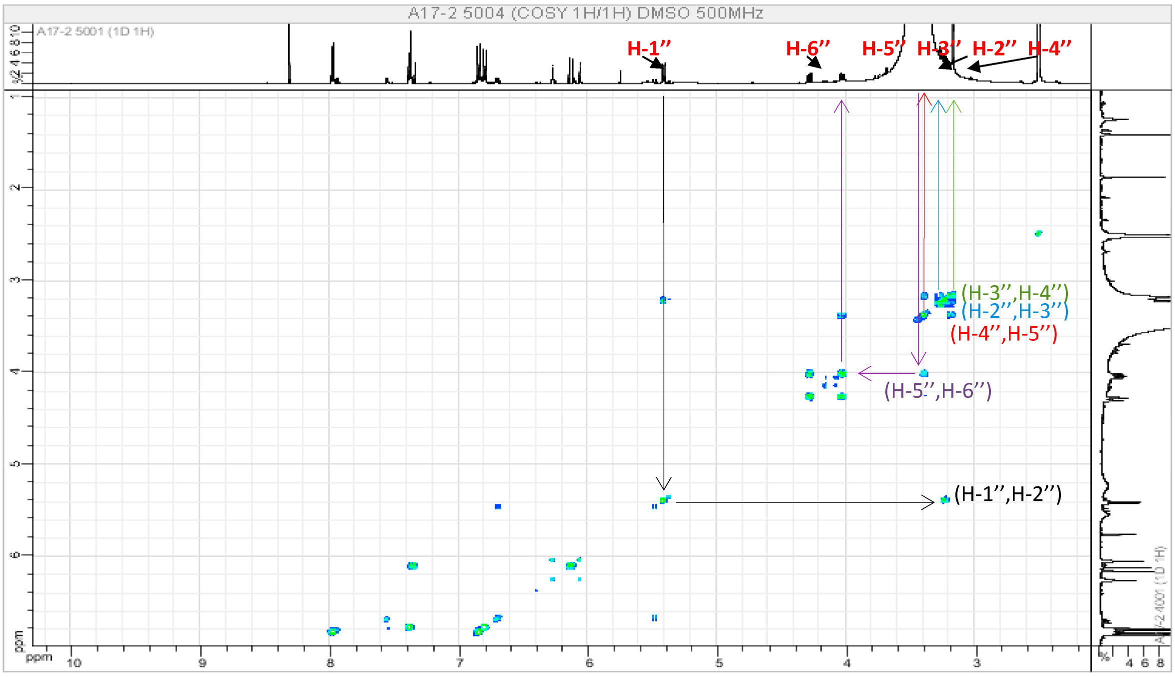

Figure S10. COSY spectrum (spreading out 1) (500 MHz, DMSO-d_6, δppm) of *trans*-tiliroside.

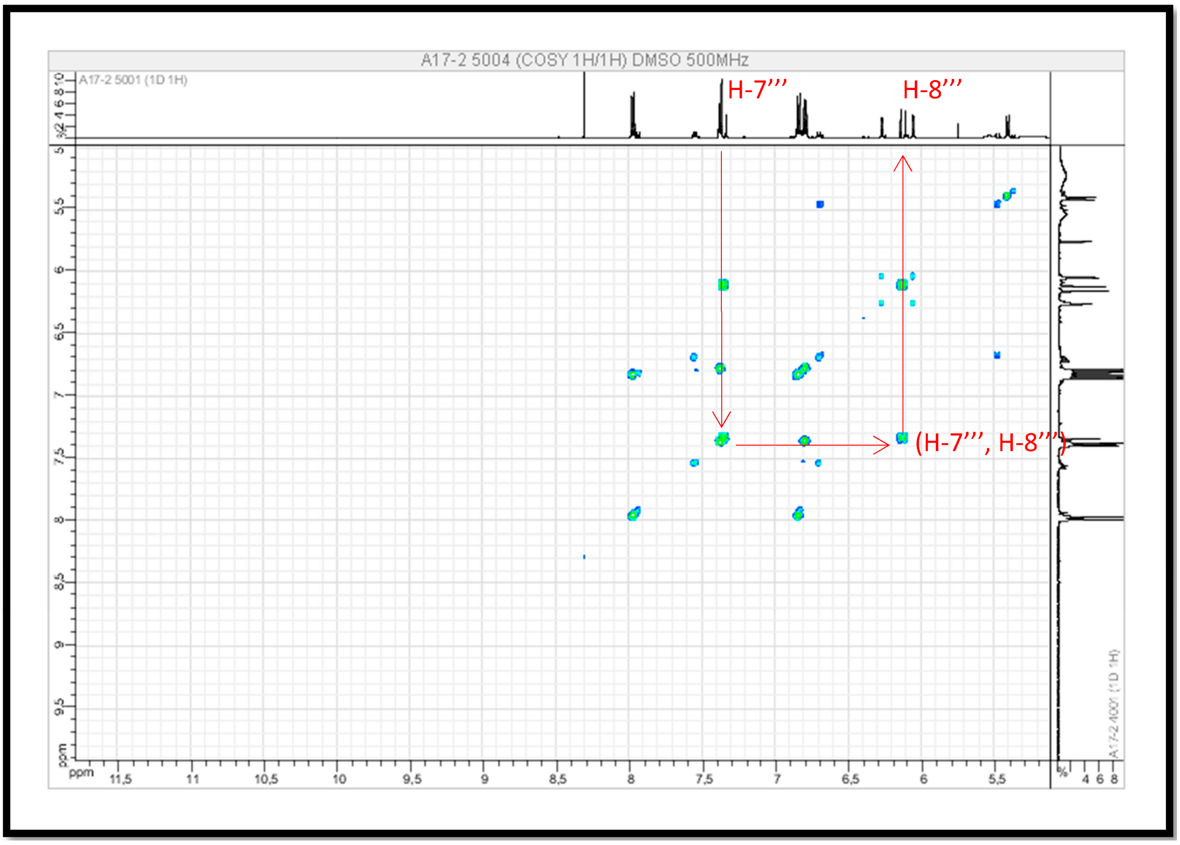

Figure S11. COSY spectrum (spreading out 2) (500 MHz, DMSO-d_6, δppm) of *trans*-tiliroside.

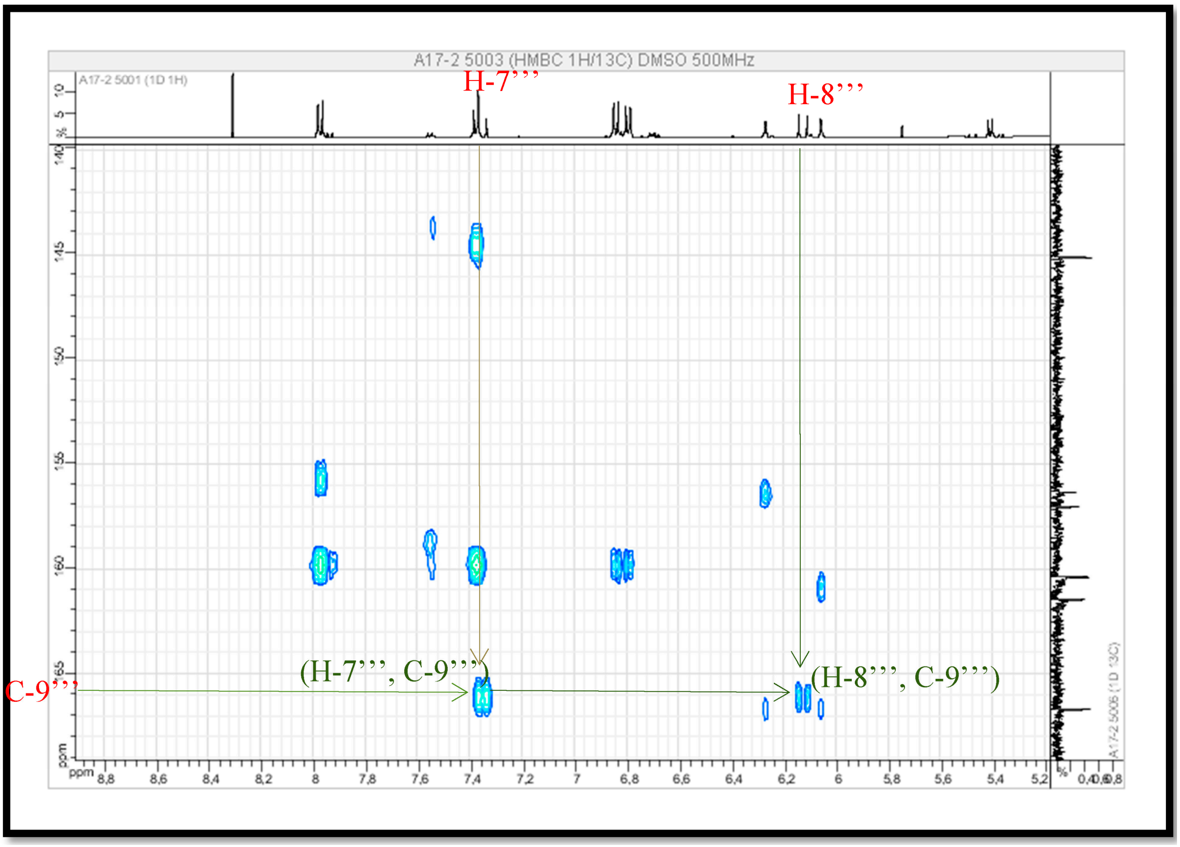

Figure S12. HMBC spectrum (spreading out 1) (500 MHz, DMSO-d_6, δppm) of *trans*-tiliroside.

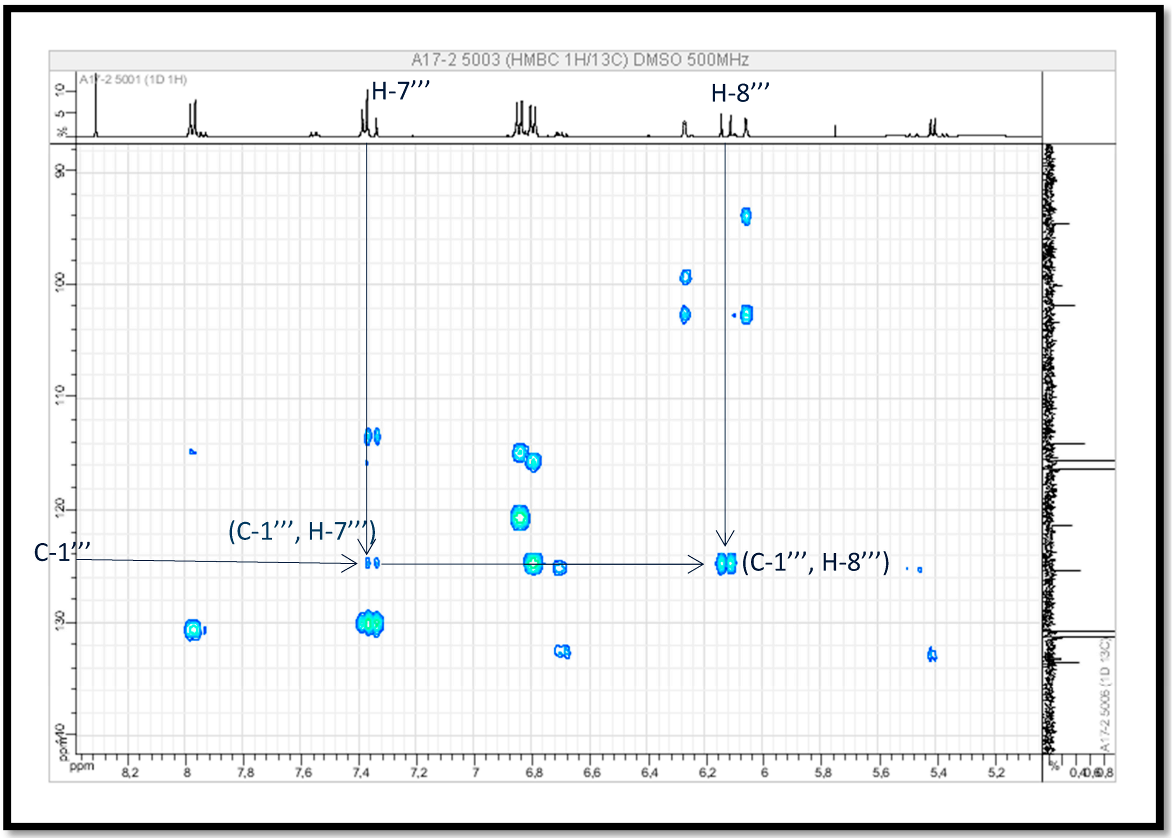

Figure S13. HMBC spectrum (spreading out 2) (500 MHz, DMSO-d_6, δppm) of *trans*-tiliroside.

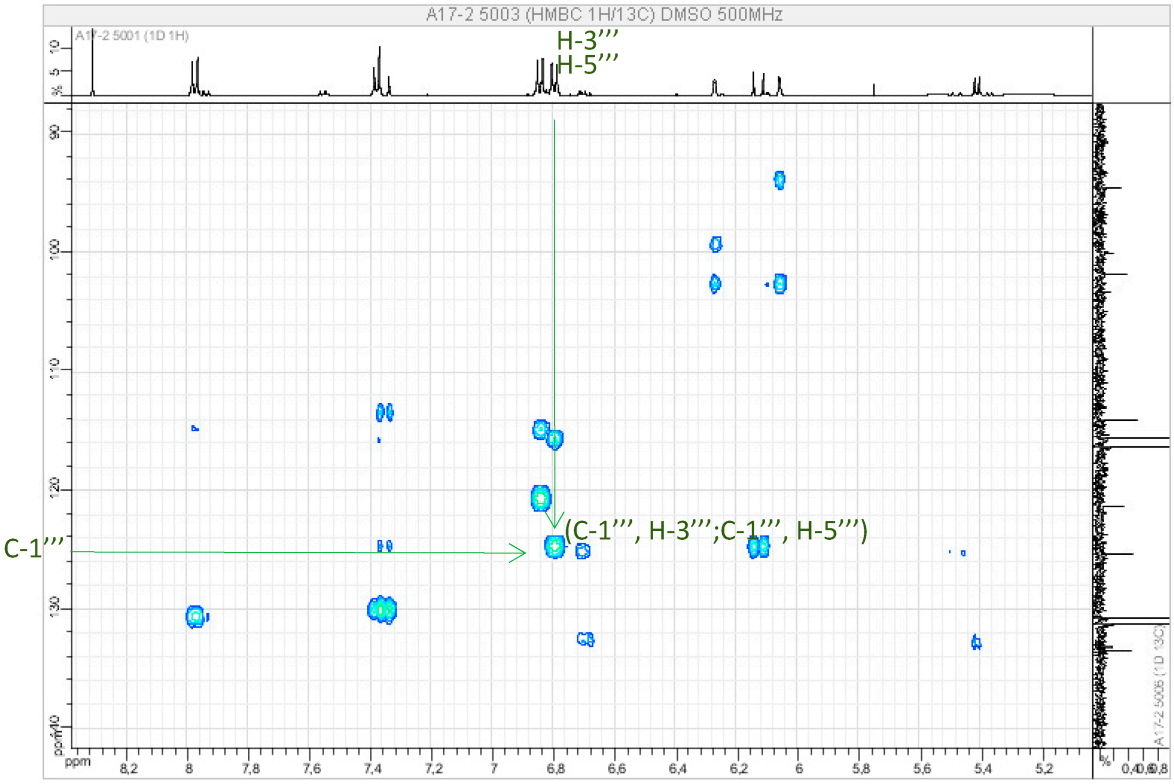

Figure S14. HMBC spectrum (spreading out 3) (500 MHz, DMSO-d_6, δppm) of *trans*-tiliroside.

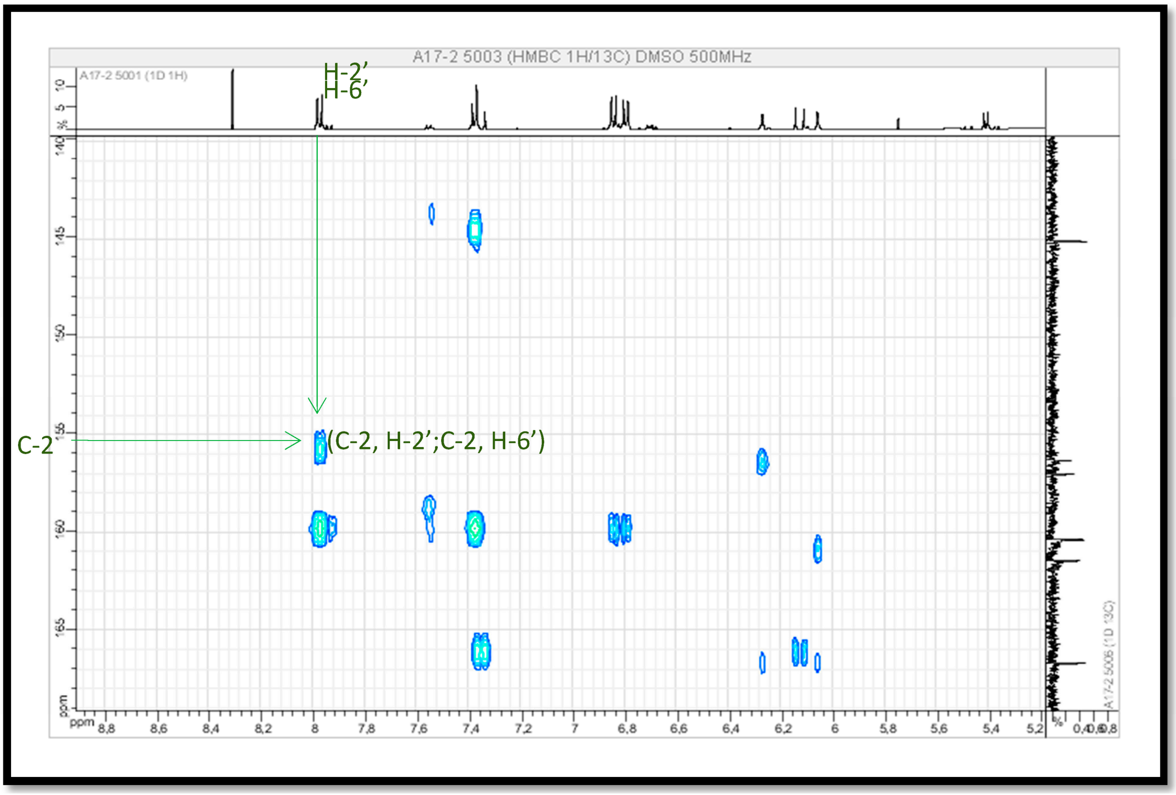

Figure S15. HMBC spectrum (spreading out 4) (500 MHz, DMSO-*d*6, δppm) of *trans*-tiliroside.

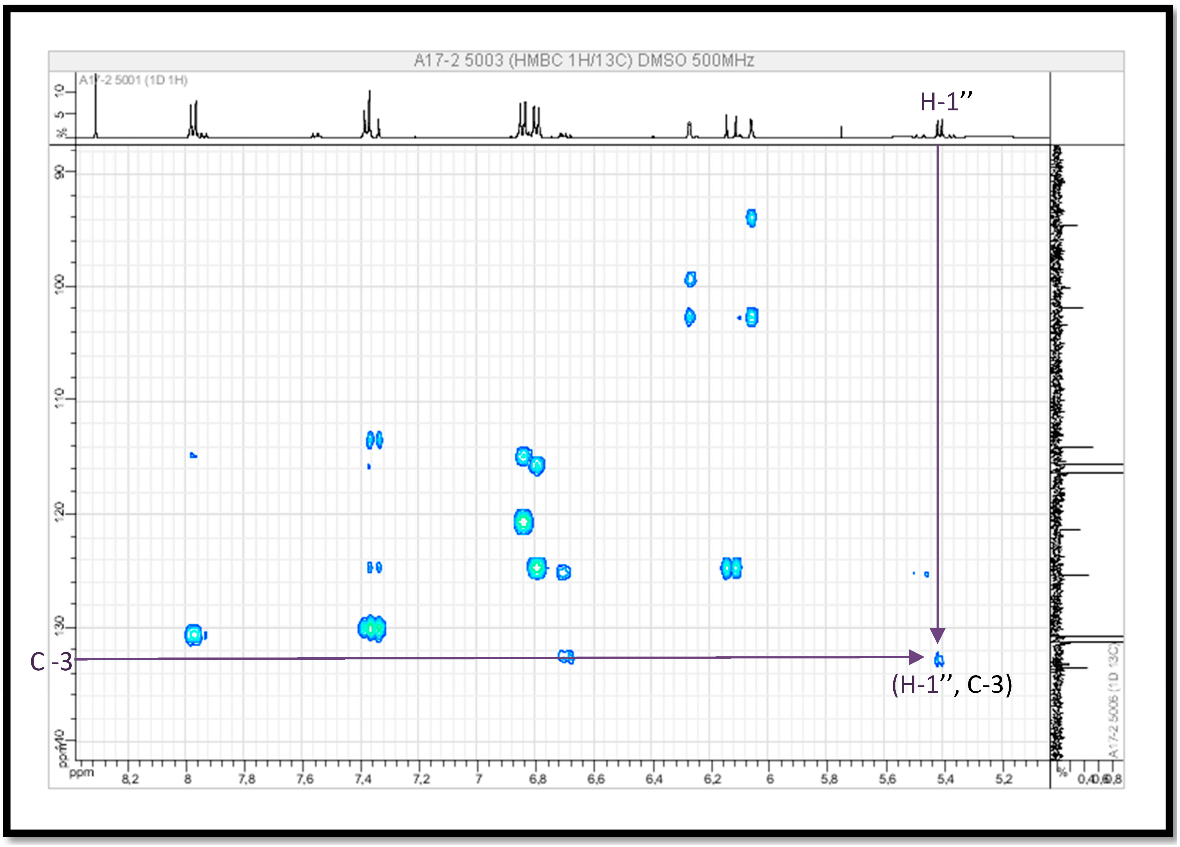

Figure S16. HMBC spectrum (spreading out 5) (500 MHz, DMSO-*d*6, δppm) of *trans*-tiliroside.

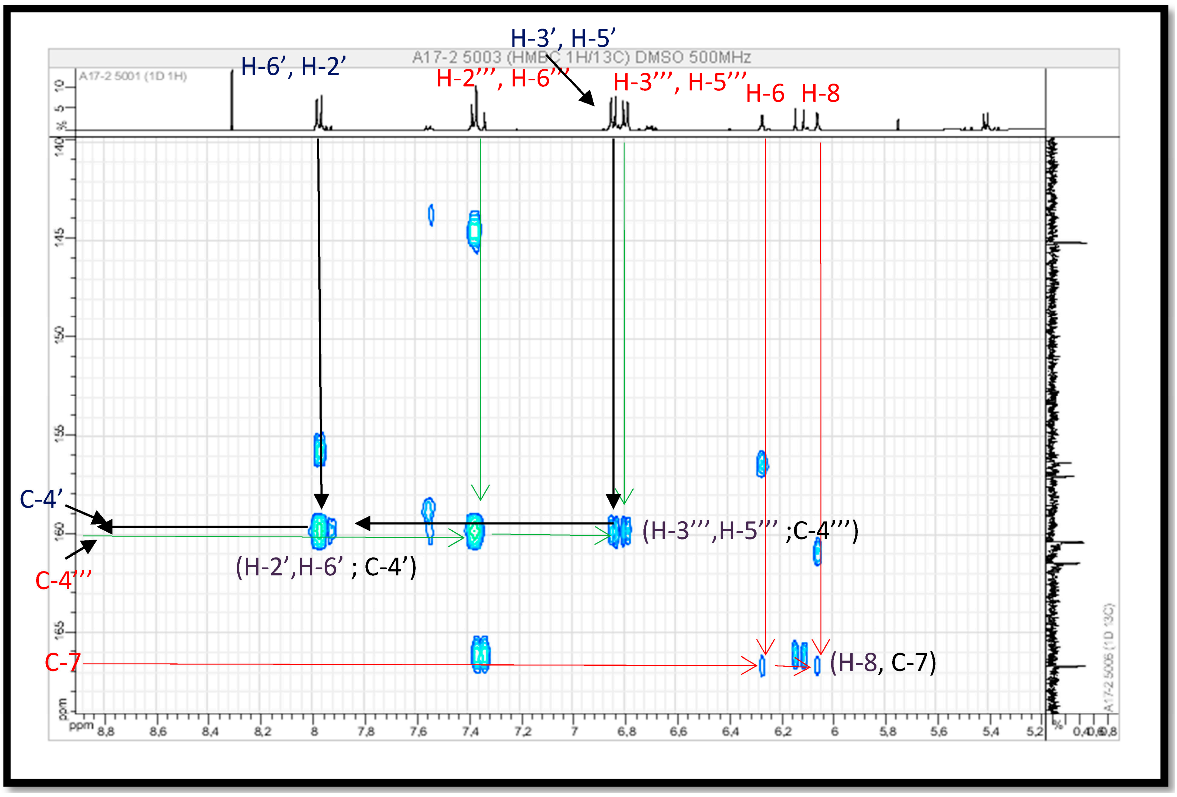

Figure S17. HMBC spectrum (spreading out 6) (500 MHz, DMSO-d_6, δppm) of *trans*-tiliroside.

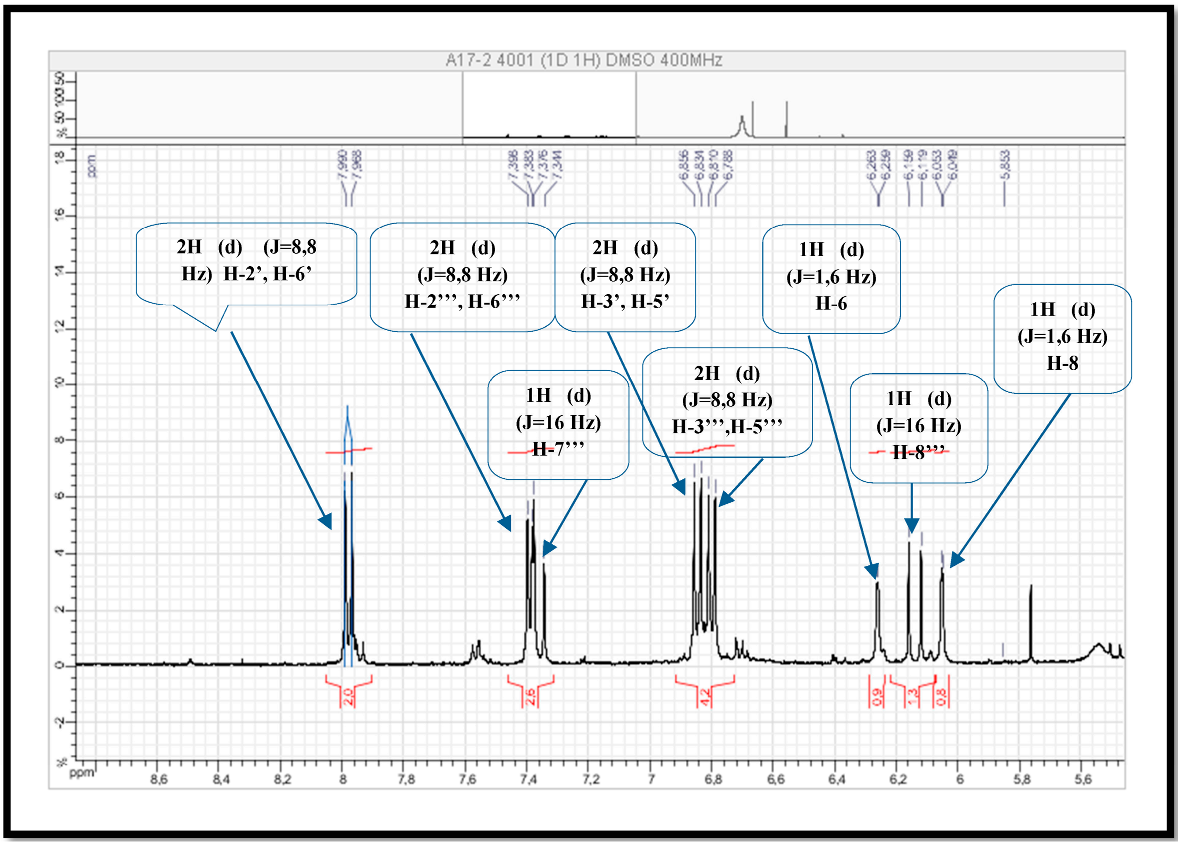

Figure S18. ^{1}H NMR spectrum (400 MHz, DMSO-d_6, δppm) of *trans*-tiliroside.

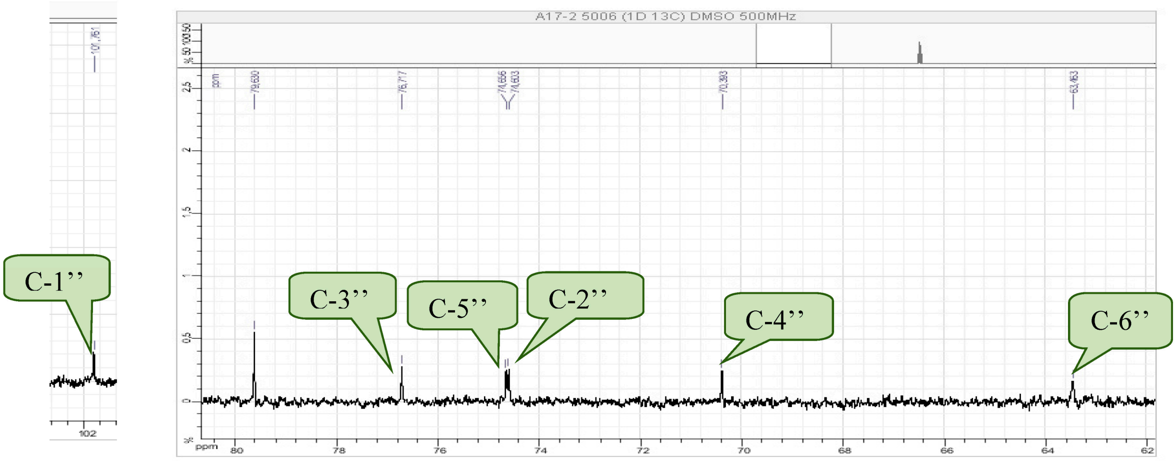

Figure S19. ^{13}C NMR spectrum (spreading out 1) (125 MHz, DMSO-d_6, δppm) of *trans*-tiliroside.

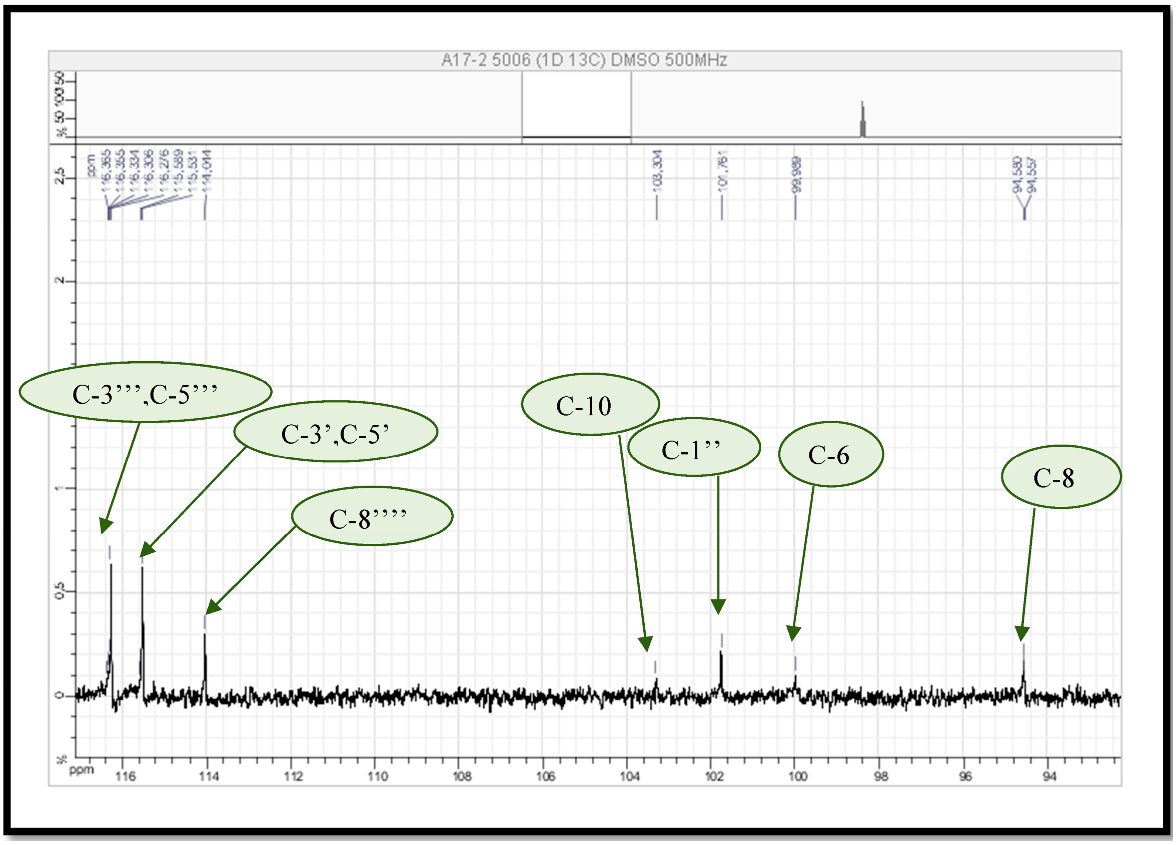

Figure S20. ^{13}C NMR spectrum (spreading out 2) (125 MHz, DMSO-d_6, δppm) of *trans*-tiliroside.

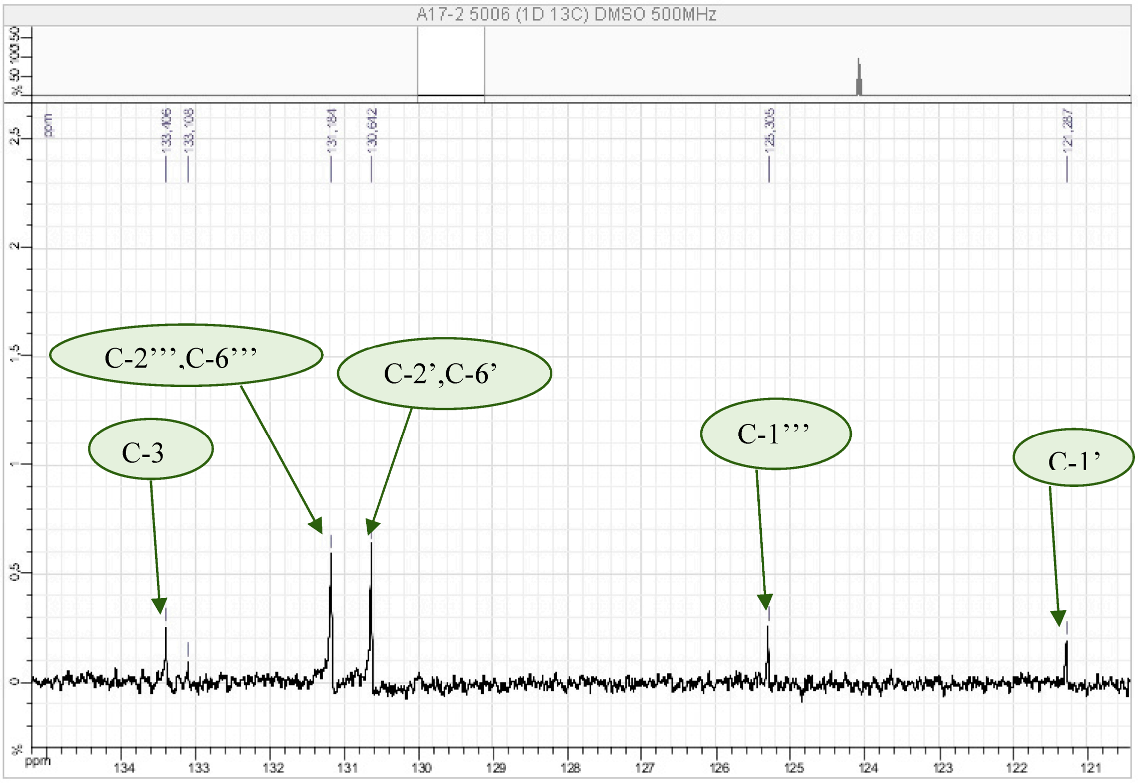

Figure S21. ^{13}C NMR spectrum (spreading out 3) (125 MHz, DMSO-*d*$_6$, δppm) of *trans*-tiliroside.

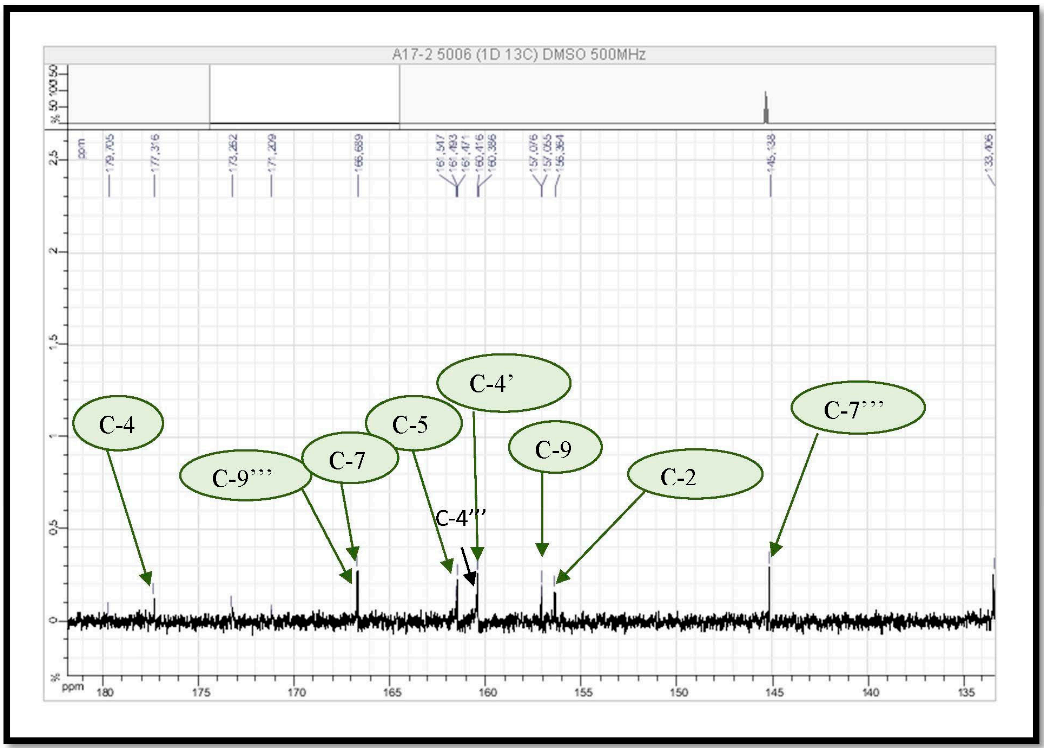

Figure S22. ^{13}C NMR spectrum (spreading out 4) (125 MHz, DMSO-*d*$_6$, δppm) of *trans*-tiliroside.

Molecule 3: *Cis*-tiliroside

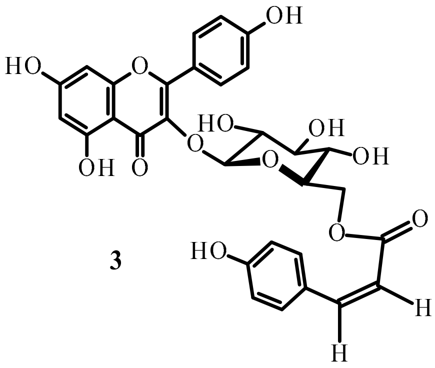

3

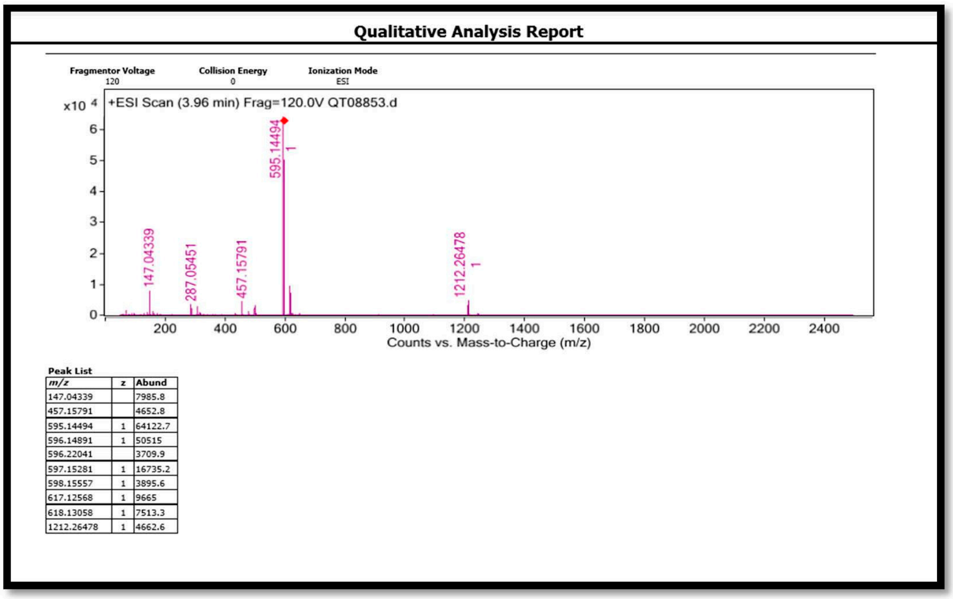

m/z	z	Abund
147.04339		7985.8
457.15791		4652.8
595.14494	1	64122.7
596.14891	1	50515
596.22041		3709.9
597.15281	1	16735.2
598.15557	1	3895.6
617.12568	1	9665
618.13058	1	7513.3
1212.26478	1	4662.6

Figure S23. ESI-HRMS(+) of *cis*-tiliroside.

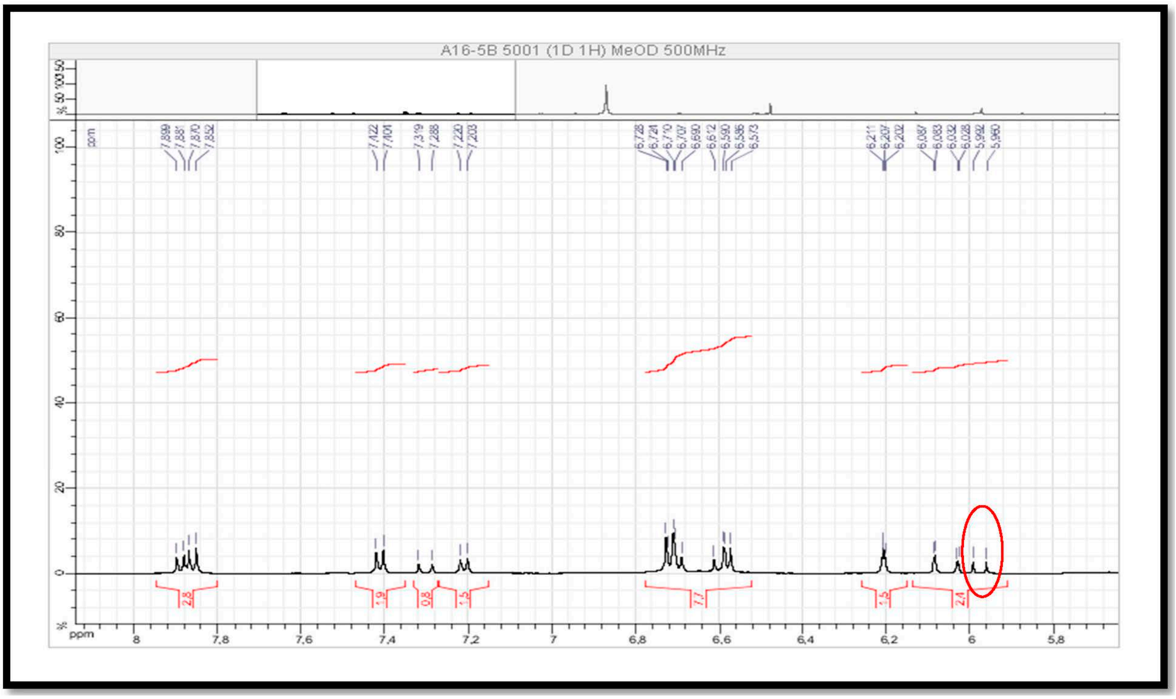

Figure S24. ^{1}H NMR spectrum (spreading out 1) (500 MHz, CD_3OD, δppm) of *cis*-tiliroside.

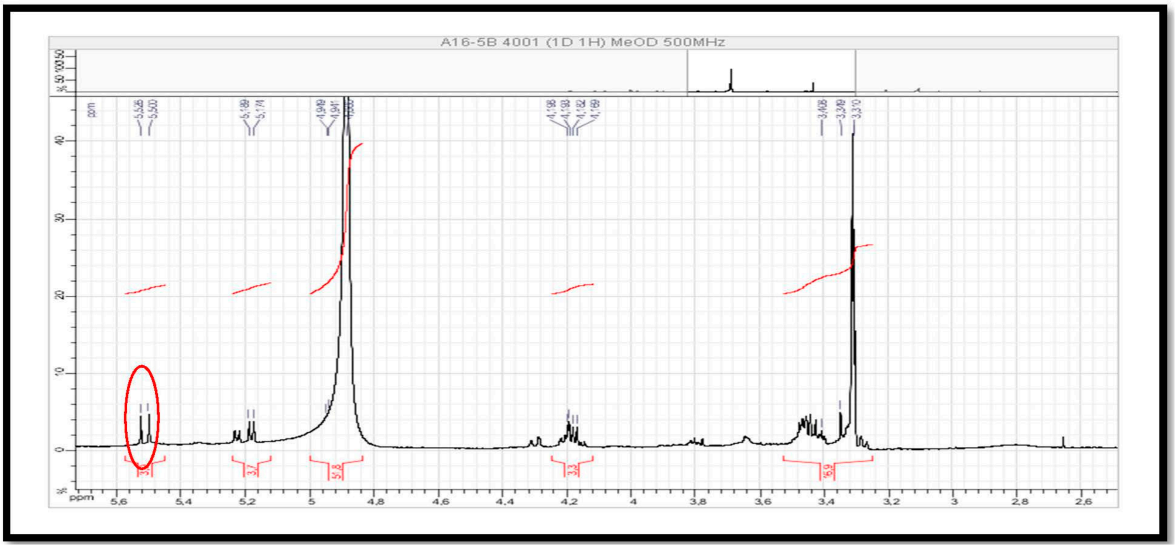

Figure S25. ^{1}H NMR spectrum (spreading out 2) (500 MHz, CD_3OD, δppm) of *cis*-tiliroside.

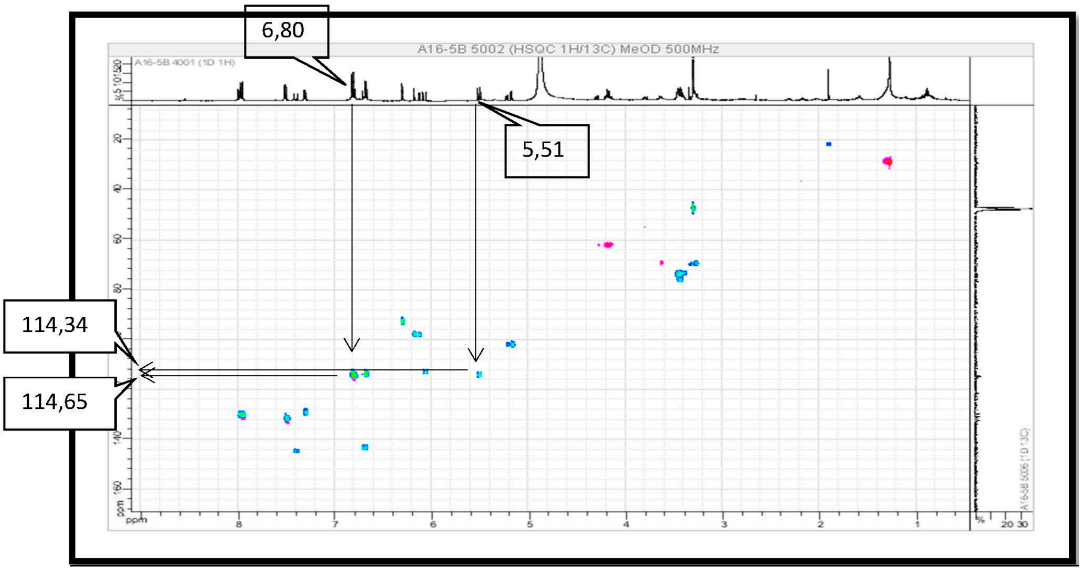

Figure S26. HSQC NMR spectrum (500 MHz, CD_3OD, δppm) of *cis*-tiliroside.

Molecule 4: Astragalin

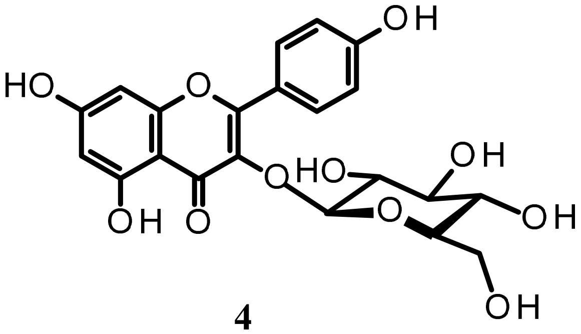

4

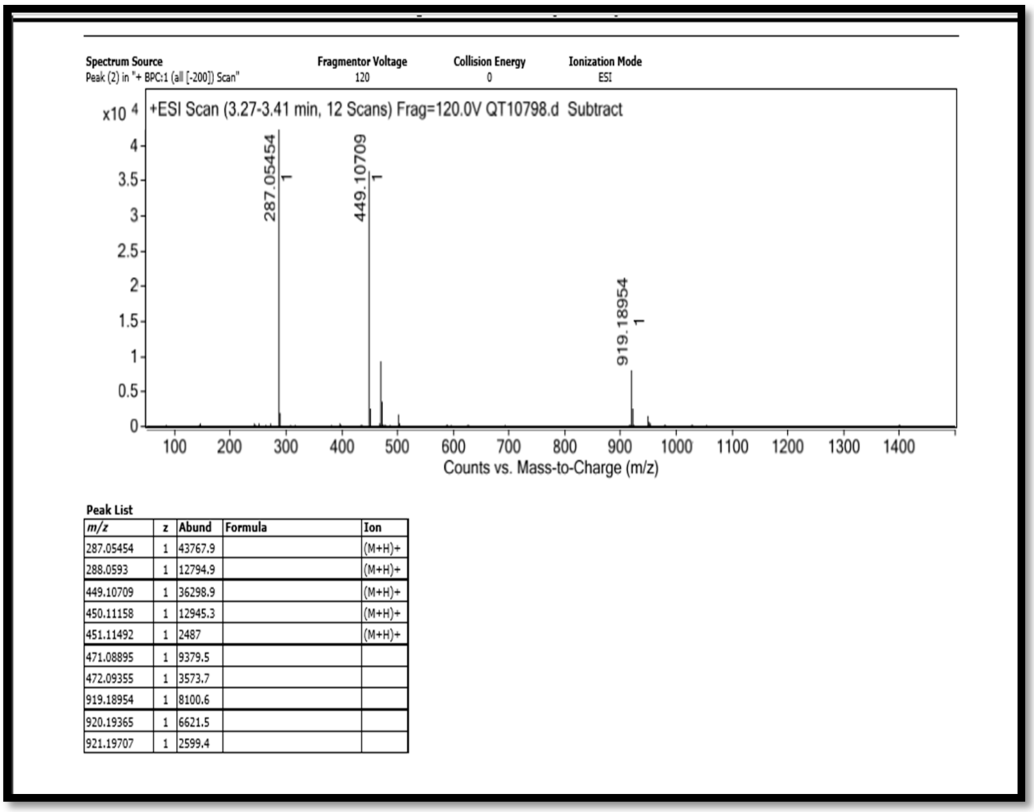

m/z	z	Abund	Formula	Ion
287.05454	1	43767.9		(M+H)+
288.0593	1	12794.9		(M+H)+
449.10709	1	36298.9		(M+H)+
450.11158	1	12945.3		(M+H)+
451.11492	1	2487		(M+H)+
471.08895	1	9379.5		
472.09355	1	3573.7		
919.18954	1	8100.6		
920.19365	1	6621.5		
921.19707	1	2599.4		

Figure S27. ESI-HRMS(+) of astragalin.

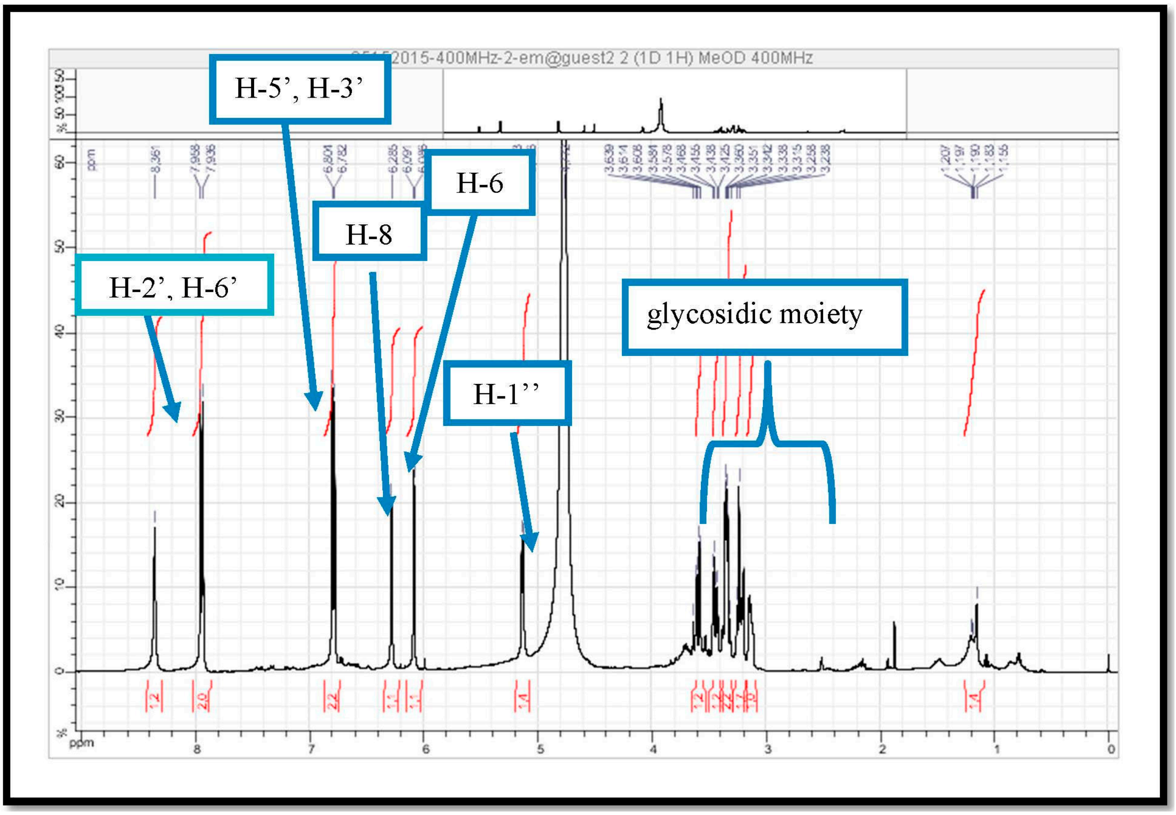

Figure S28. ^{1}H NMR spectrum (400 MHz, CD_3OD, δppm) of astragalin.

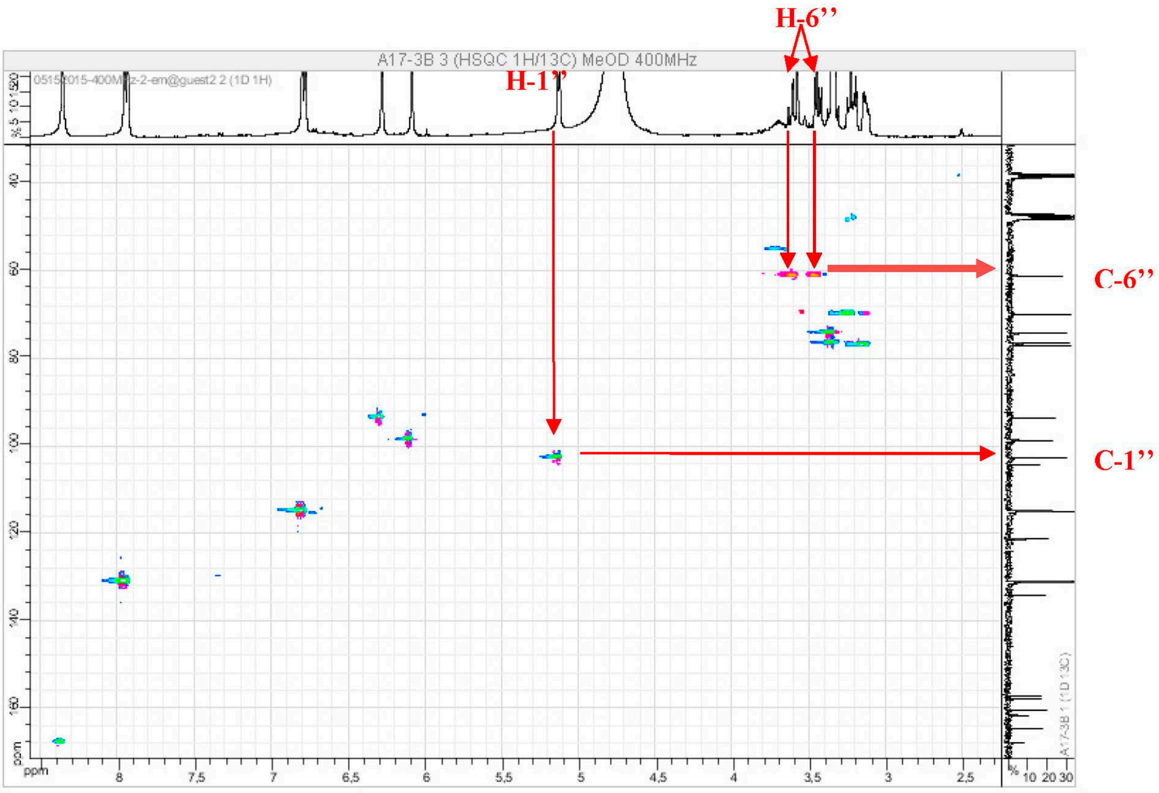

Figure S29. HSQC spectrum (400 MHz, CD_3OD, δppm) of astragalin.

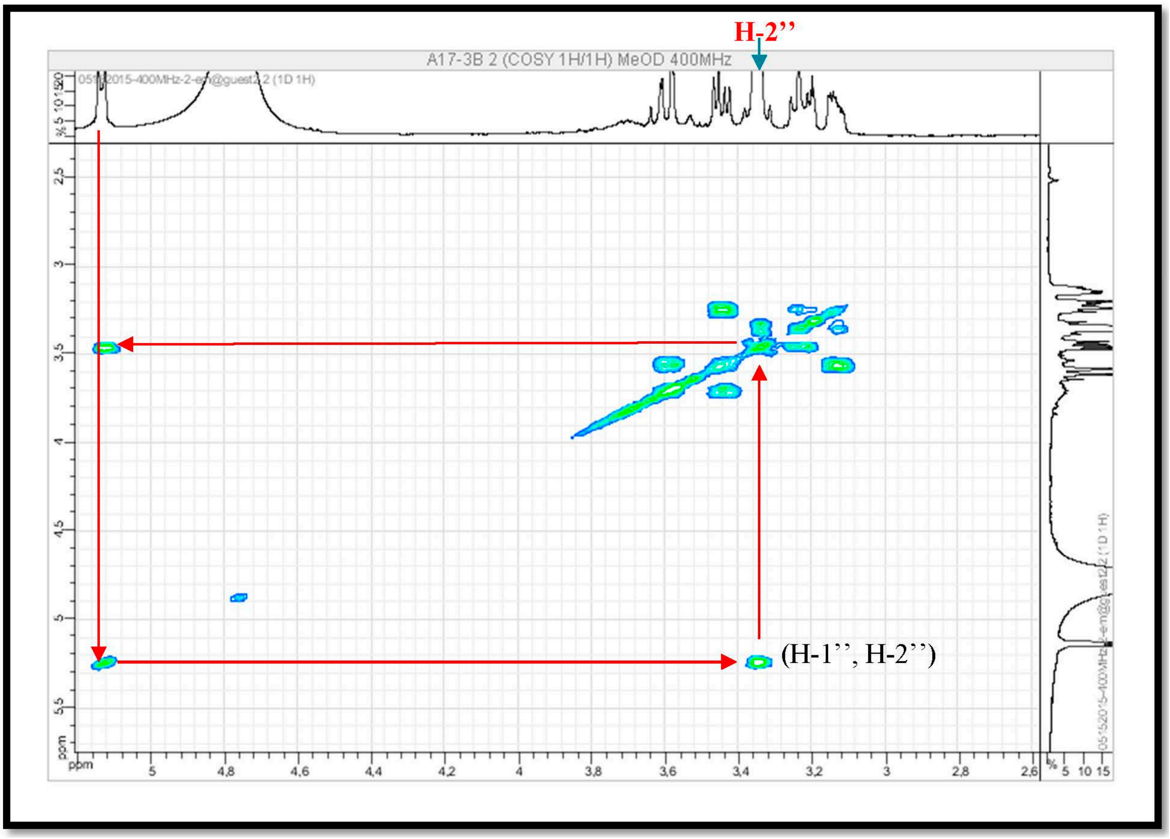

Figure S30. COSY spectrum (400 MHz, CD_3OD, δppm) of astragalin.

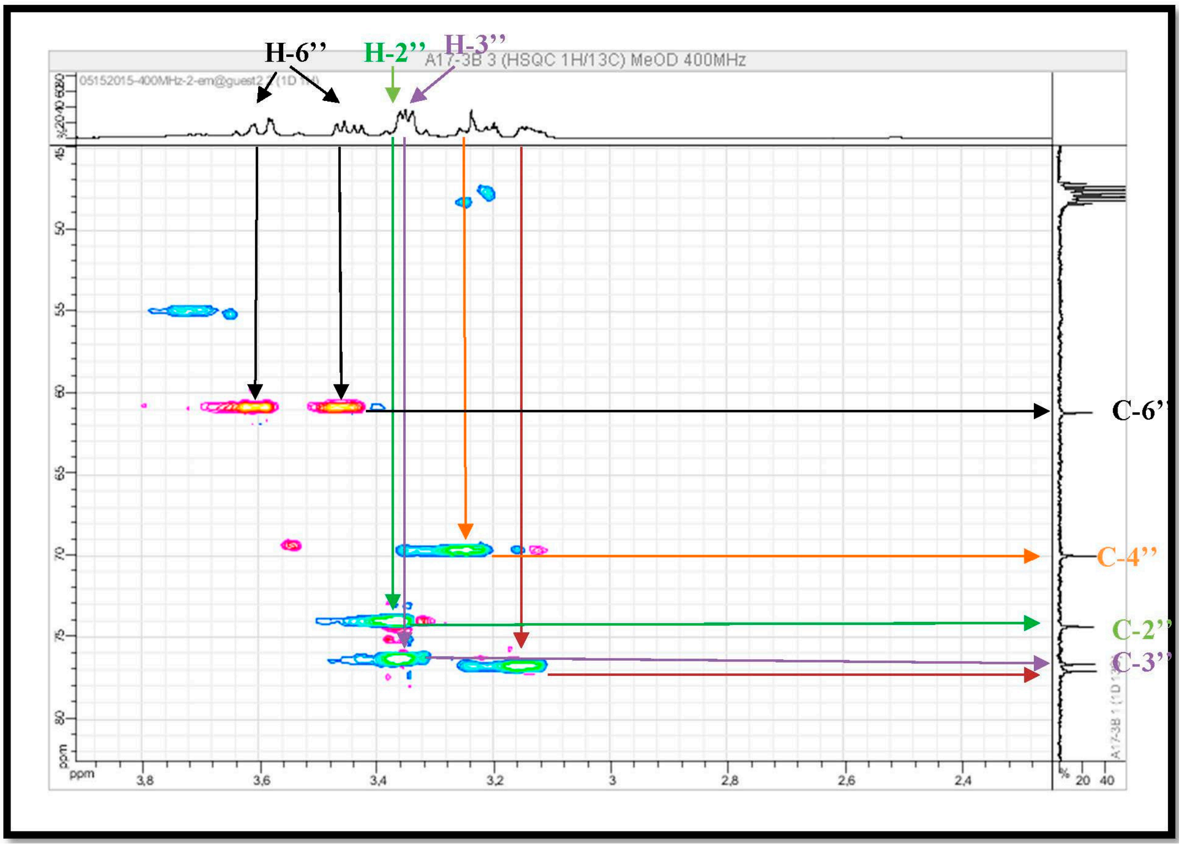

Figure S31. HSQC spectrum (400 MHz, CD_3OD, δppm) of astragalin.

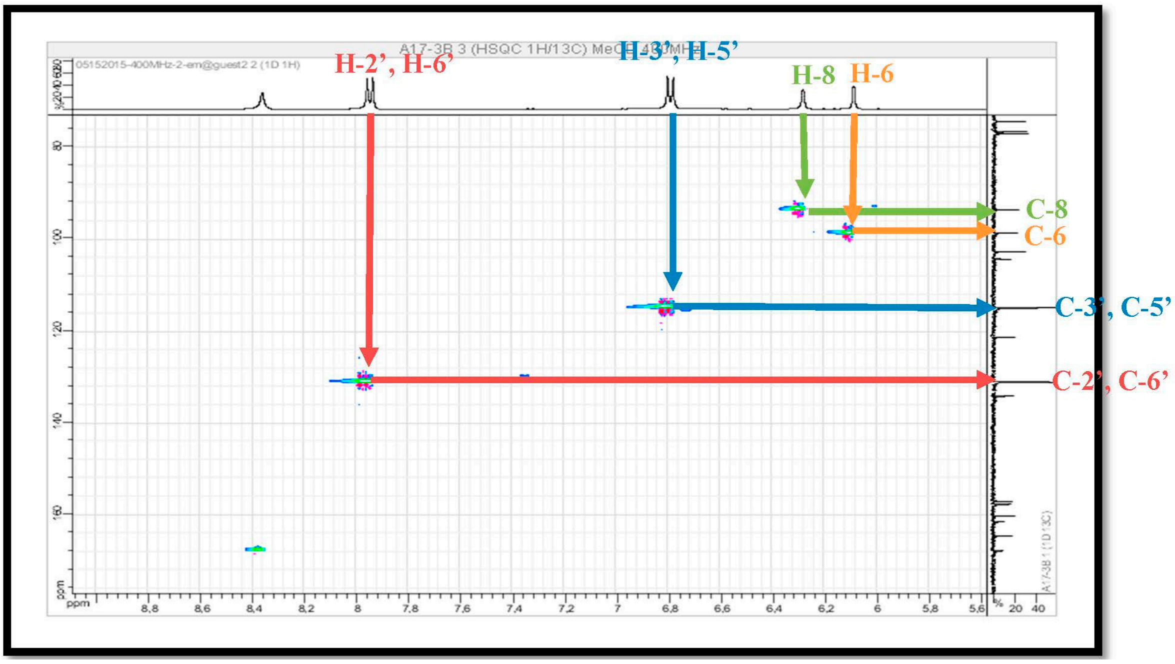

Figure S32. HSQC spectrum (spreading out 1) (400 MHz, CD_3OD, δppm) of astragalin.

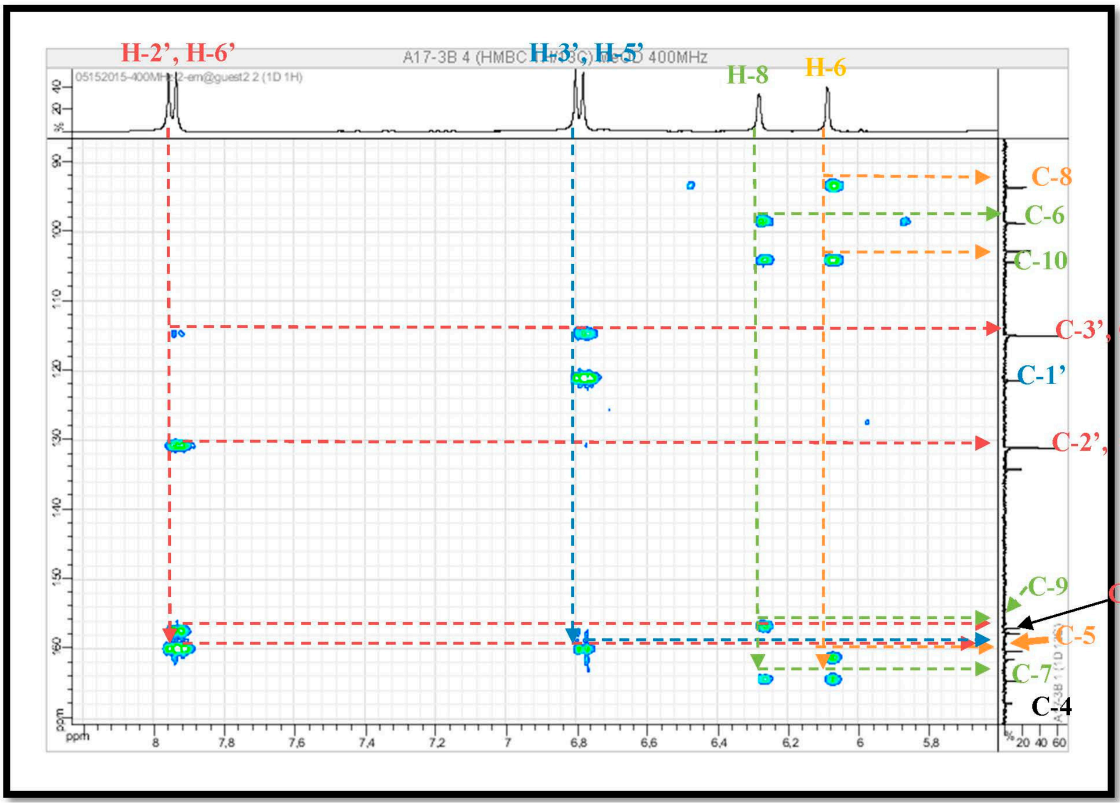

Figure S33. HMBC spectrum (spreading out1) (400 MHz, CD3OD, δppm) of astragalin.

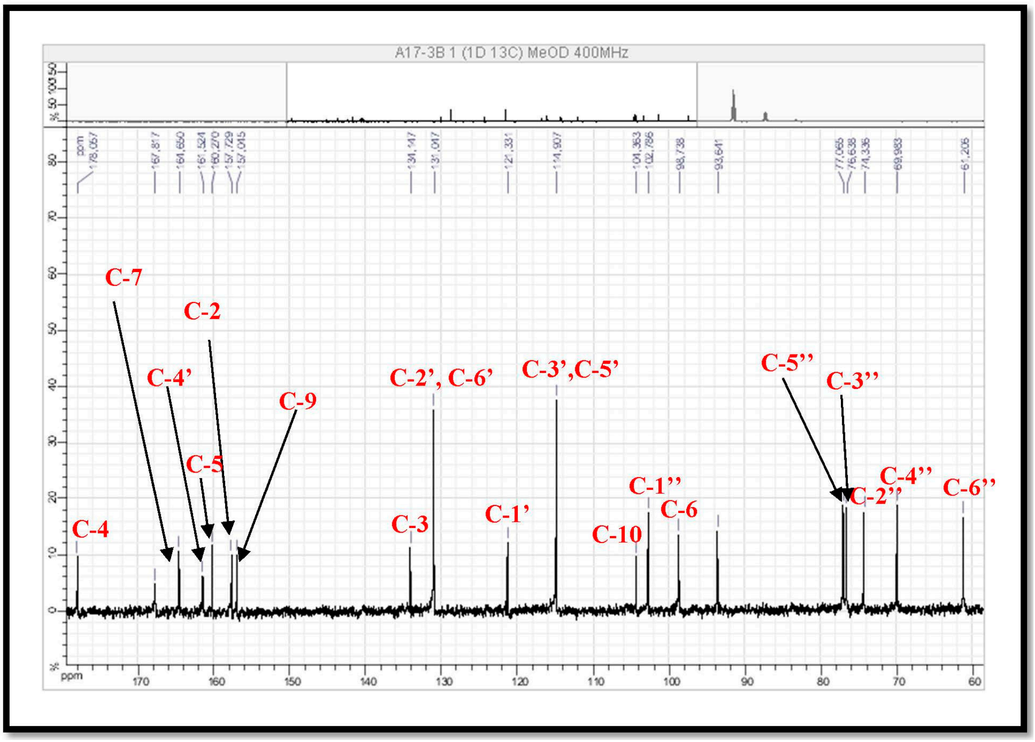

Figure S34. ^{13}C spectrum (100 MHz, CD3OD, δppm) of astragalin.

Molecule 7: Picein

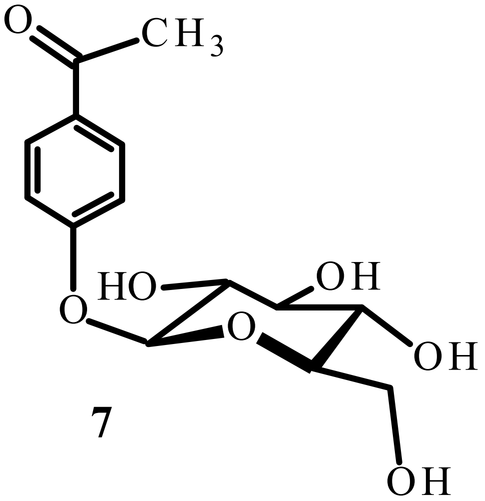

7

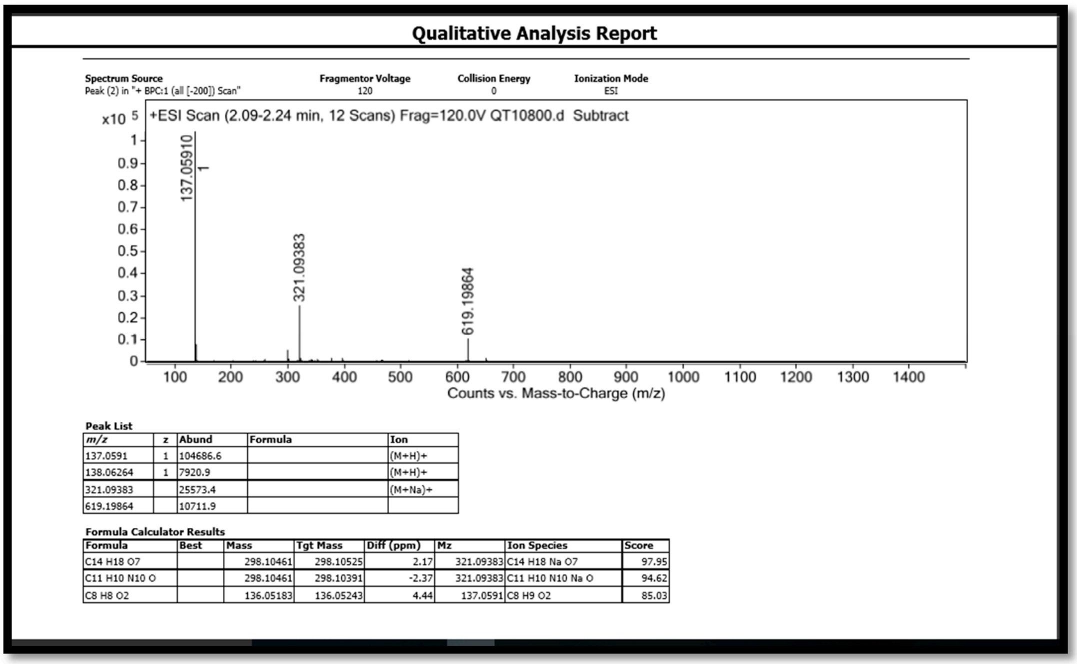

Peak List

m/z	z	Abund	Formula	Ion
137.0591	1	104686.6		(M+H)+
138.06264	1	7920.9		(M+H)+
321.09383		25573.4		(M+Na)+
619.19864		10711.9		

Formula Calculator Results

Formula	Best	Mass	Tgt Mass	Diff (ppm)	Mz	Ion Species	Score
C14 H18 O7		298.10461	298.10525	2.17	321.09383	C14 H18 Na O7	97.95
C11 H10 N10 O		298.10461	298.10391	-2.37	321.09383	C11 H10 N10 Na O	94.62
C8 H8 O2		136.05183	136.05243	4.44	137.0591	C8 H9 O2	85.03

Figure S35. ESI-HRMS(+) of picein.

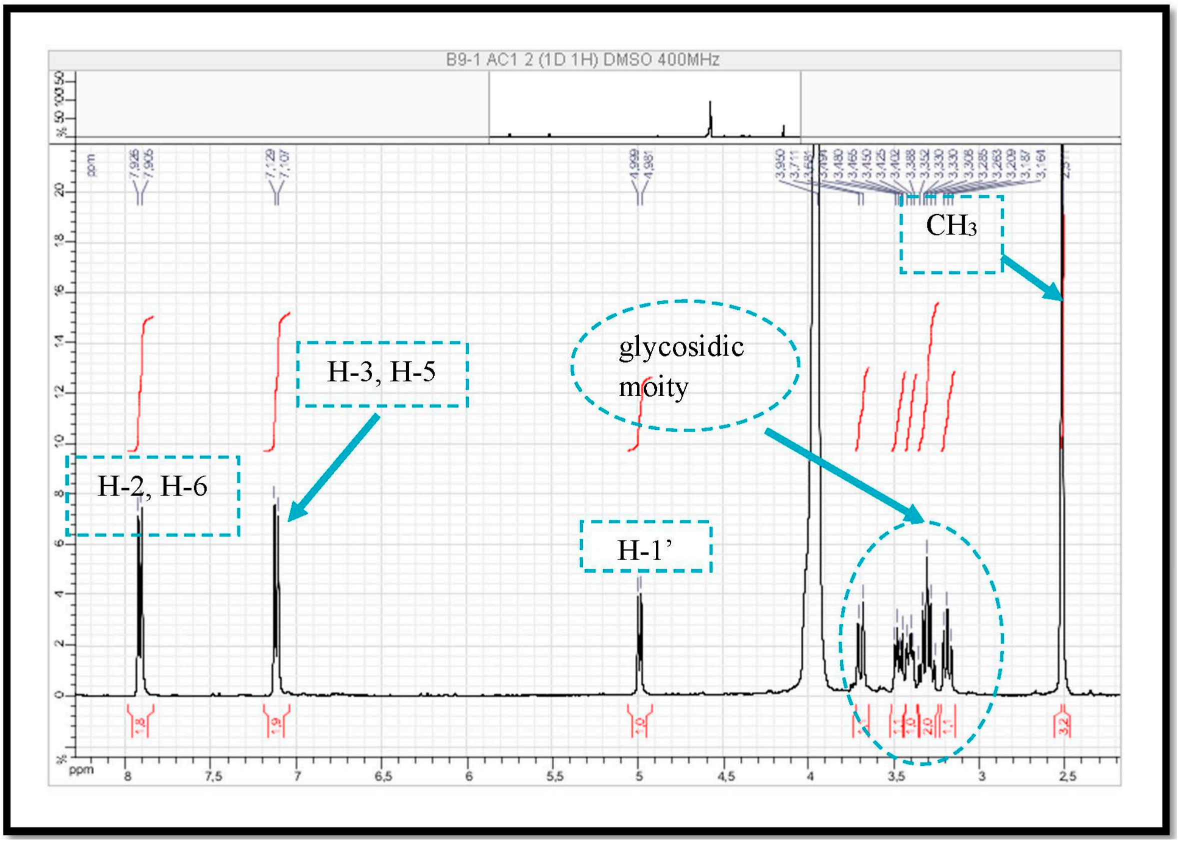

Figure S36. ^{1}H NMR spectrum (spreading out1) (400 MHz, DMSO-d_6, δppm) of picein.

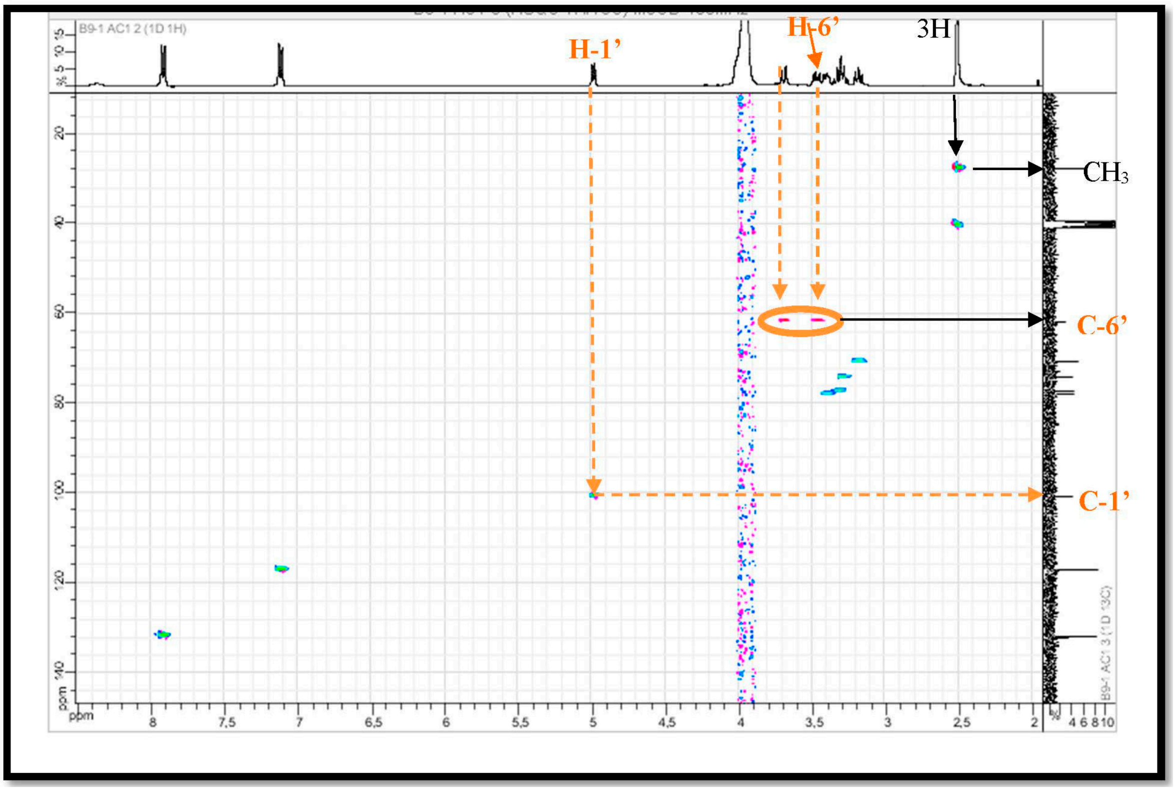

Figure S37. HSQC NMR spectrum (400 MHz, DMSO-d_6, δppm) of picein.

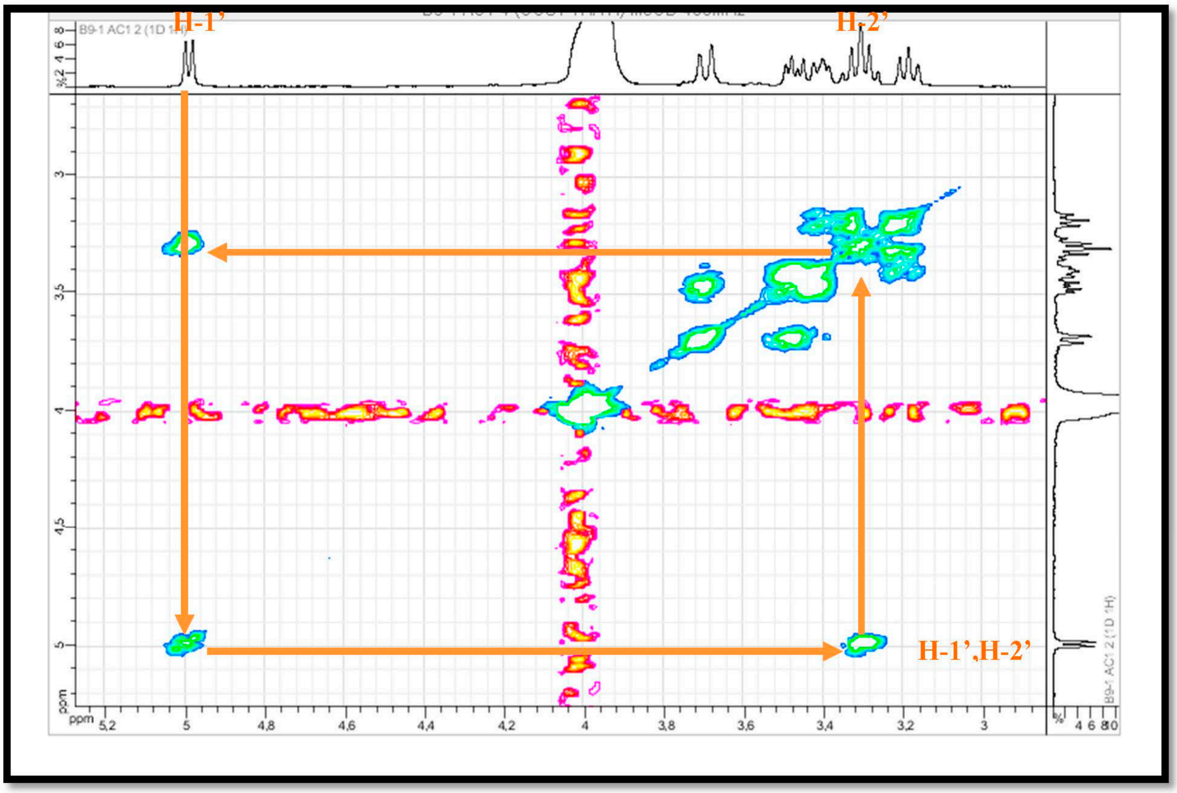

Figure S38. COSY NMR spectrum (400 MHz, DMSO-d_6, δppm) of picein.

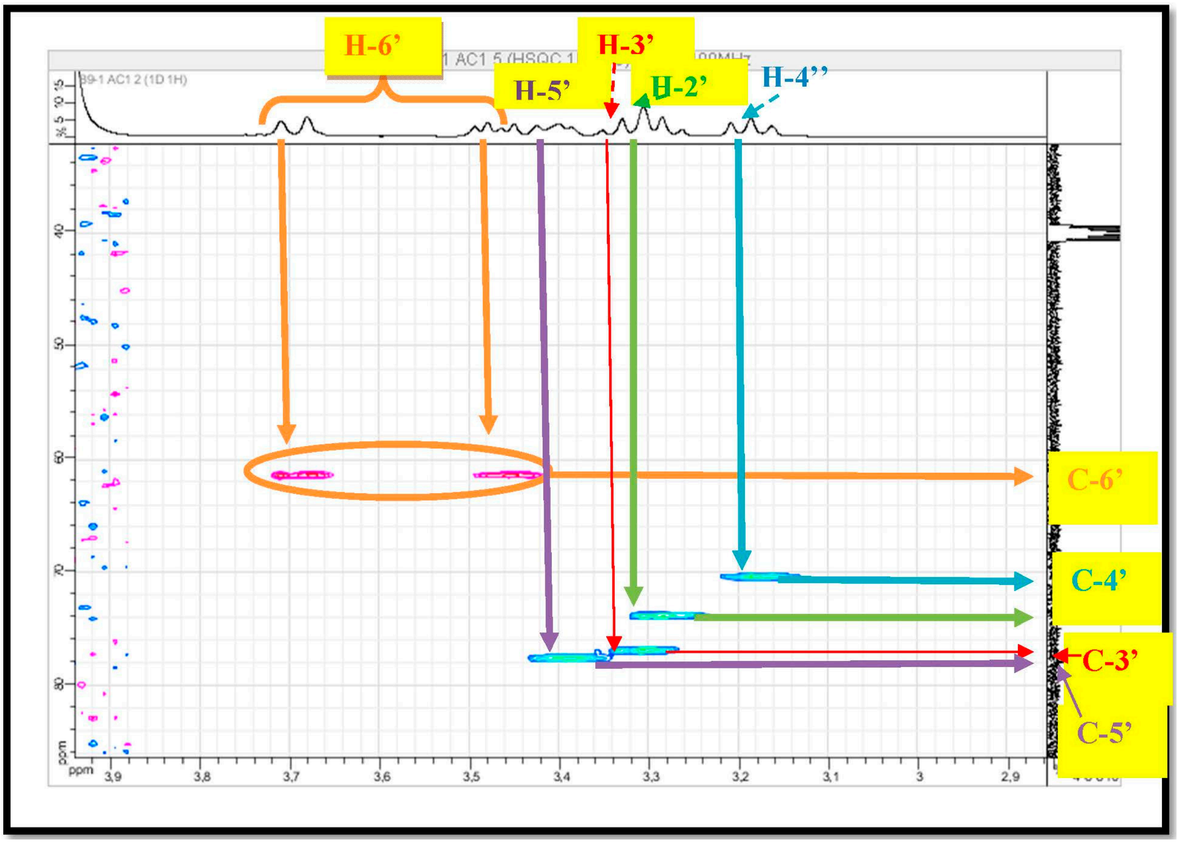

Figure S39. HSQC NMR spectrum (spreading out 1) (400 MHz, DMSO-d_6, δppm) of picein.

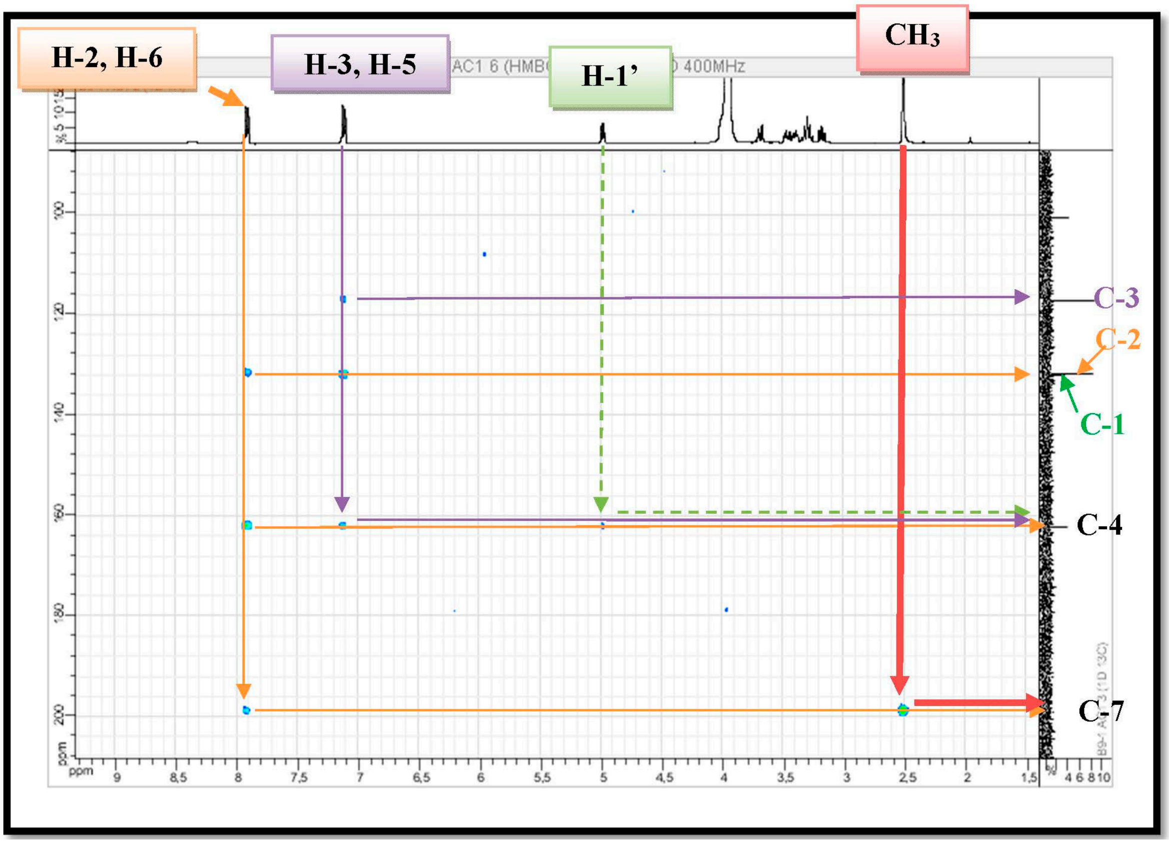

Figure S40. HMBC NMR spectrum (400 MHz, DMSO-d_6, δppm) of picein.

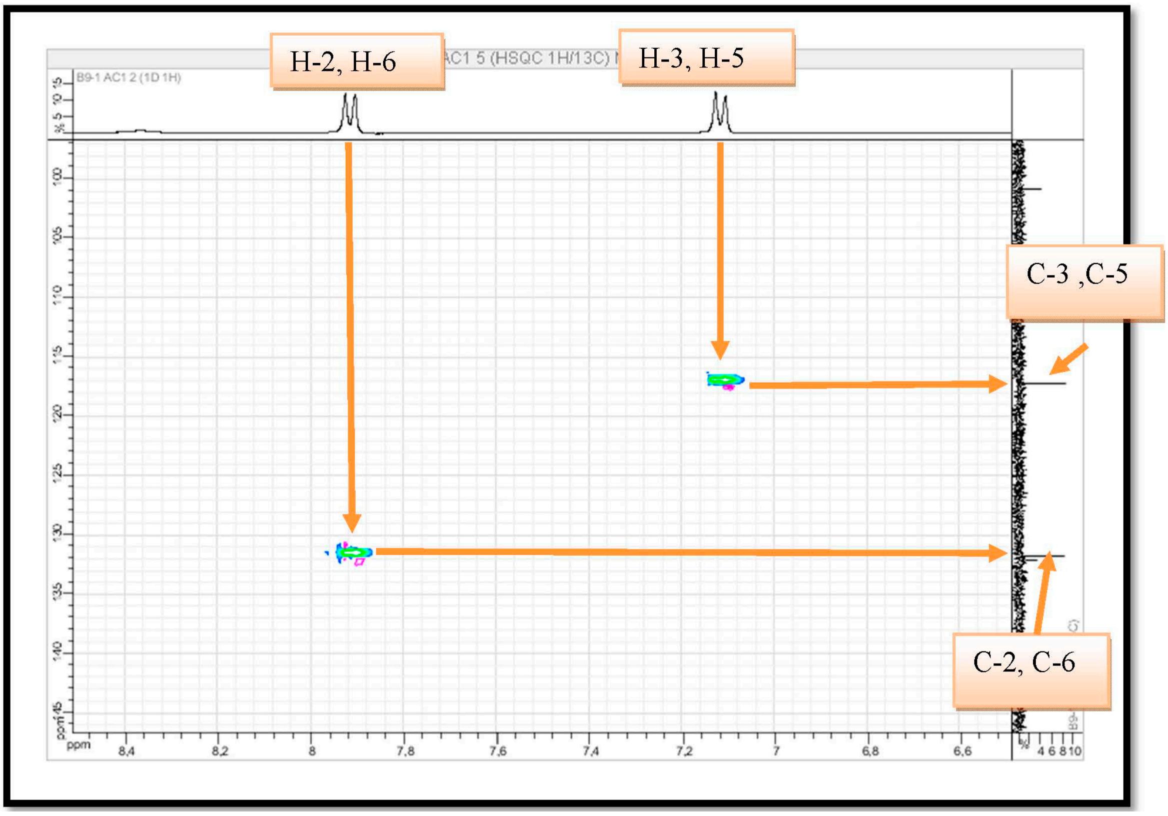

Figure S41. HSQC NMR spectrum (spreading out 2) (400 MHz, DMSO-d_6, δppm) of picein.

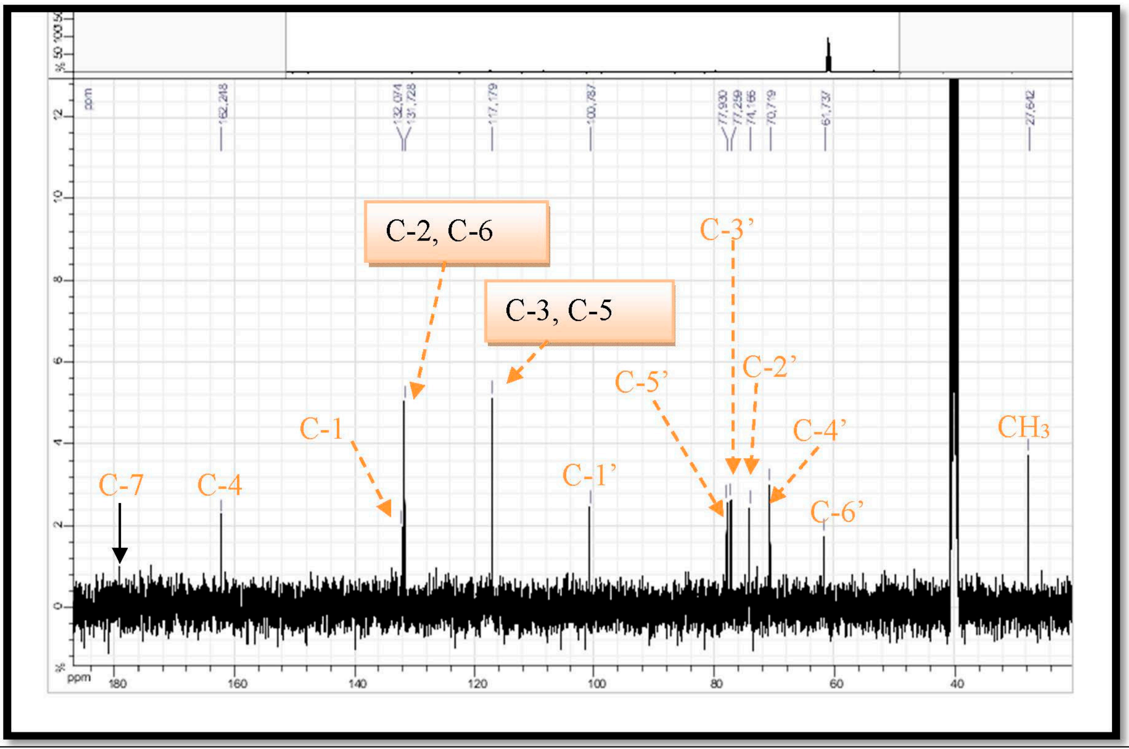

Figure S42. ^{13}C NMR spectrum (spreading out 1) (100 MHz, DMSO-d_6, δppm) of picein.

Molecule 8: Vanillic acid 4-*O*-β-D-glucopyranoside

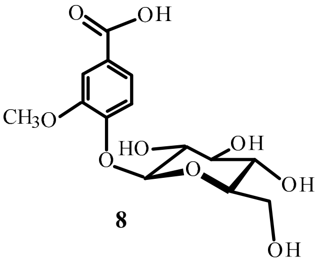

8

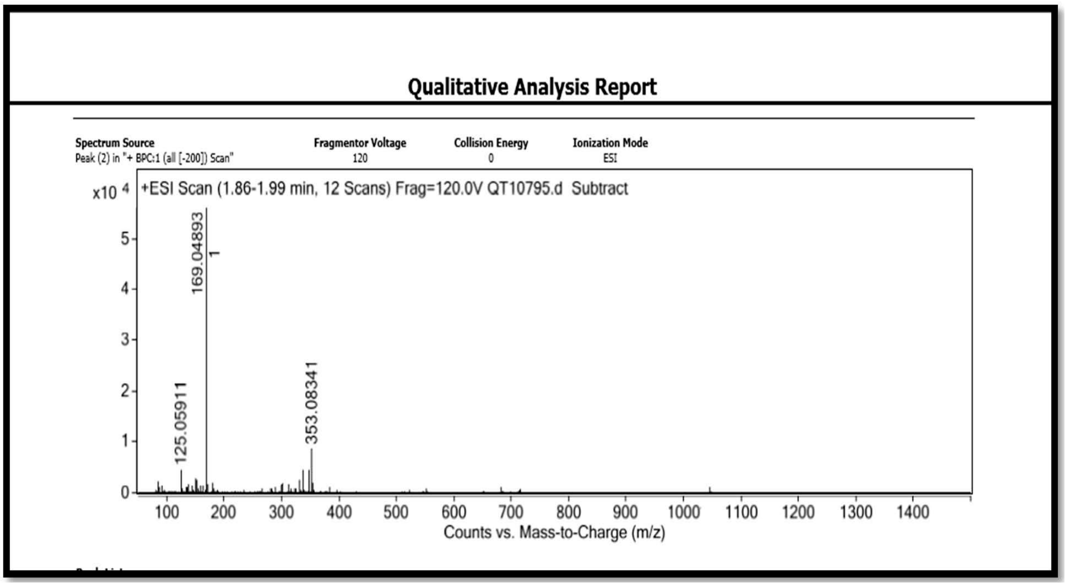

Figure S43. ESI-HRMS(+) of Vanillic acid 4-*O*-β-D-glucopyranoside.

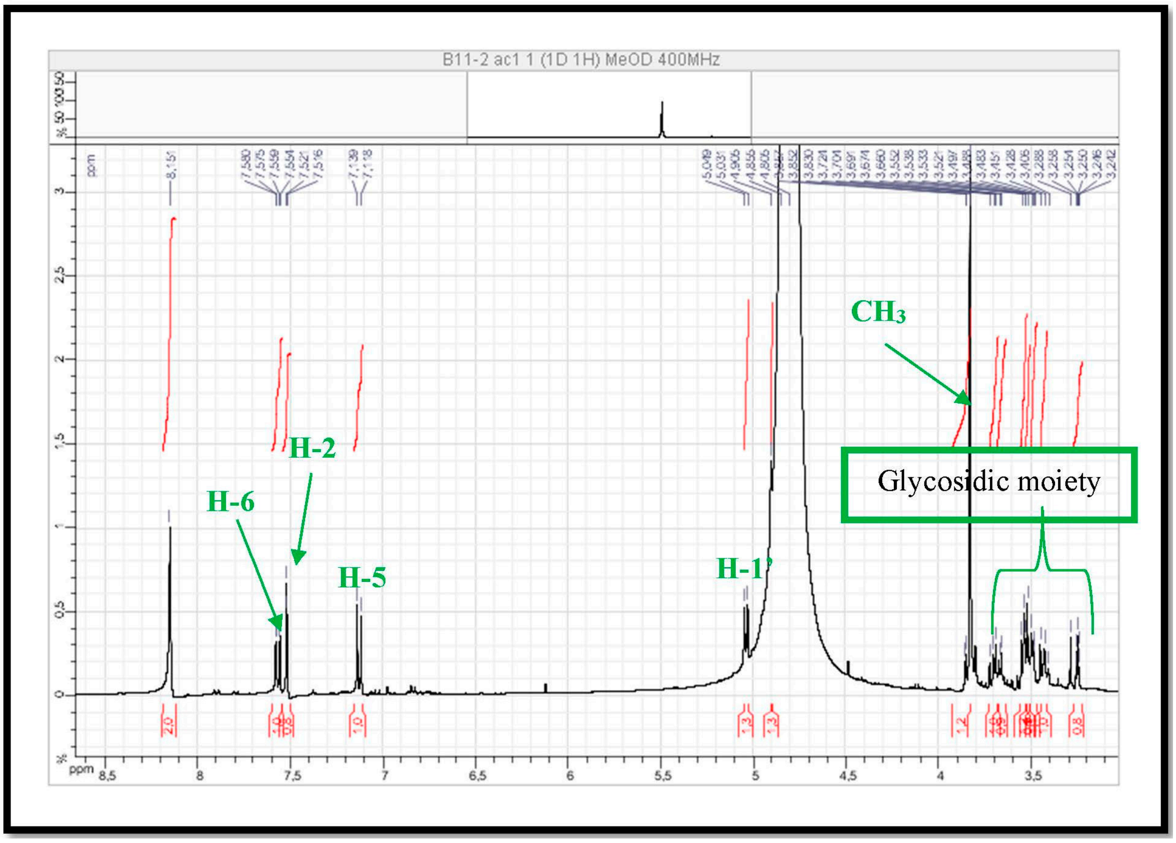

Figure S44. ^{1}H NMR spectrum (400 MHz, CD_3OD, δppm) of Vanillic acid 4-*O*-β-D-glucopyranoside.

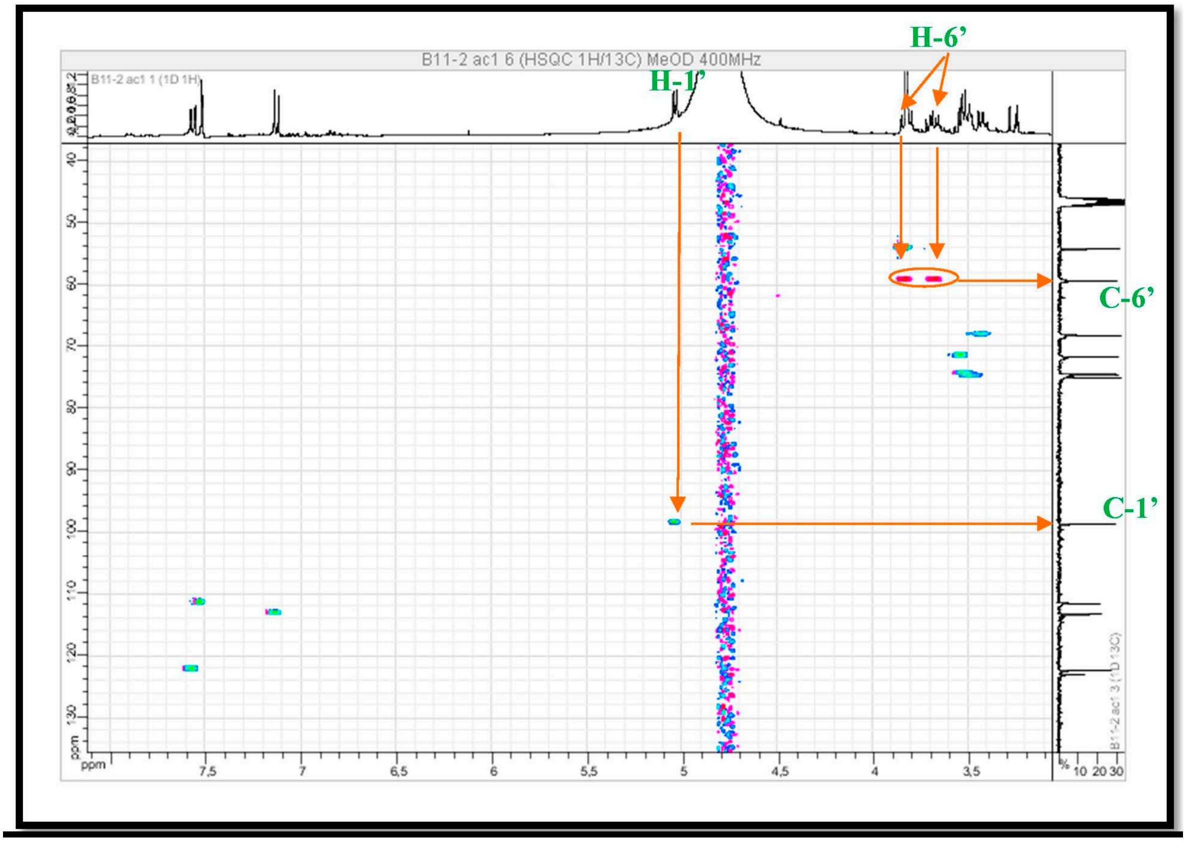

Figure S45. HSQC NMR spectrum (400 MHz, CD_3OD, δppm) of Vanillic acid 4-*O*-β-D-glucopyranoside.

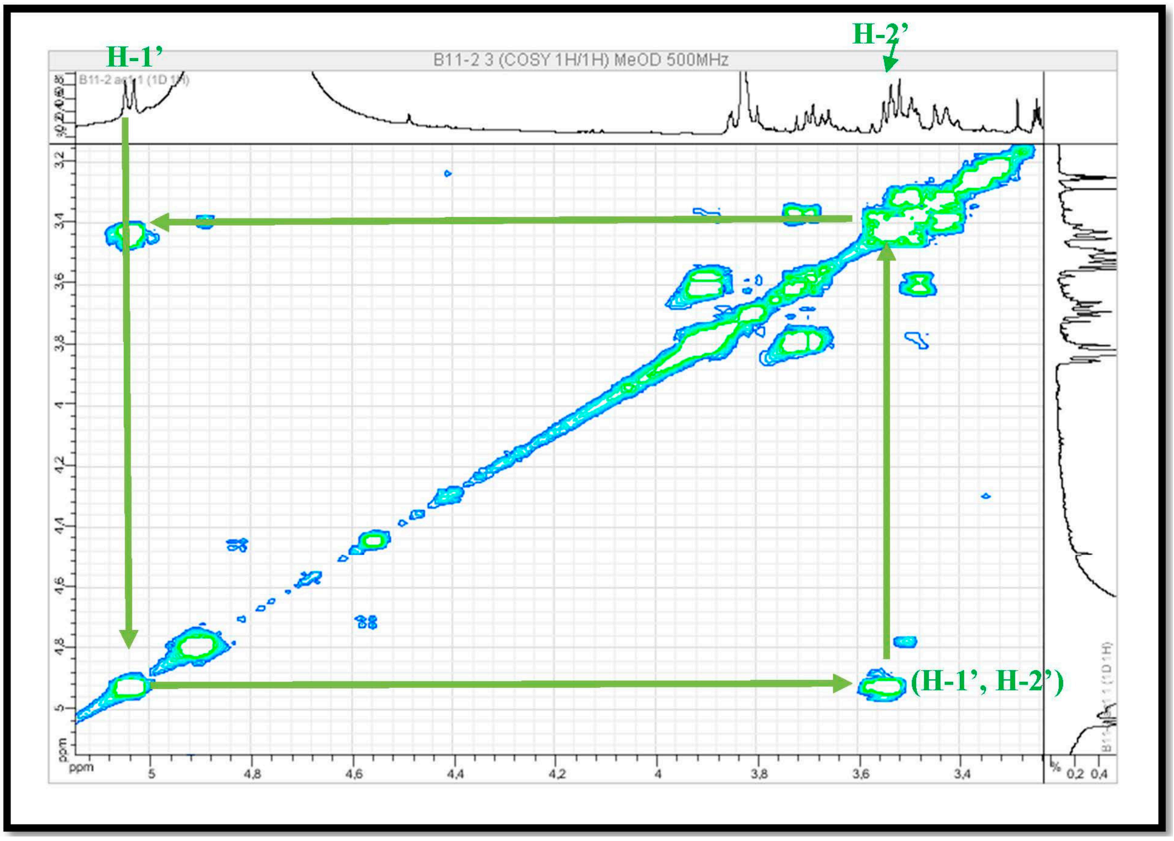

Figure S46. COSY NMR spectrum (spreading out 1) (400 MHz, CD_3OD, δppm) of Vanillic acid 4-*O*-β-D-glucopyranoside.

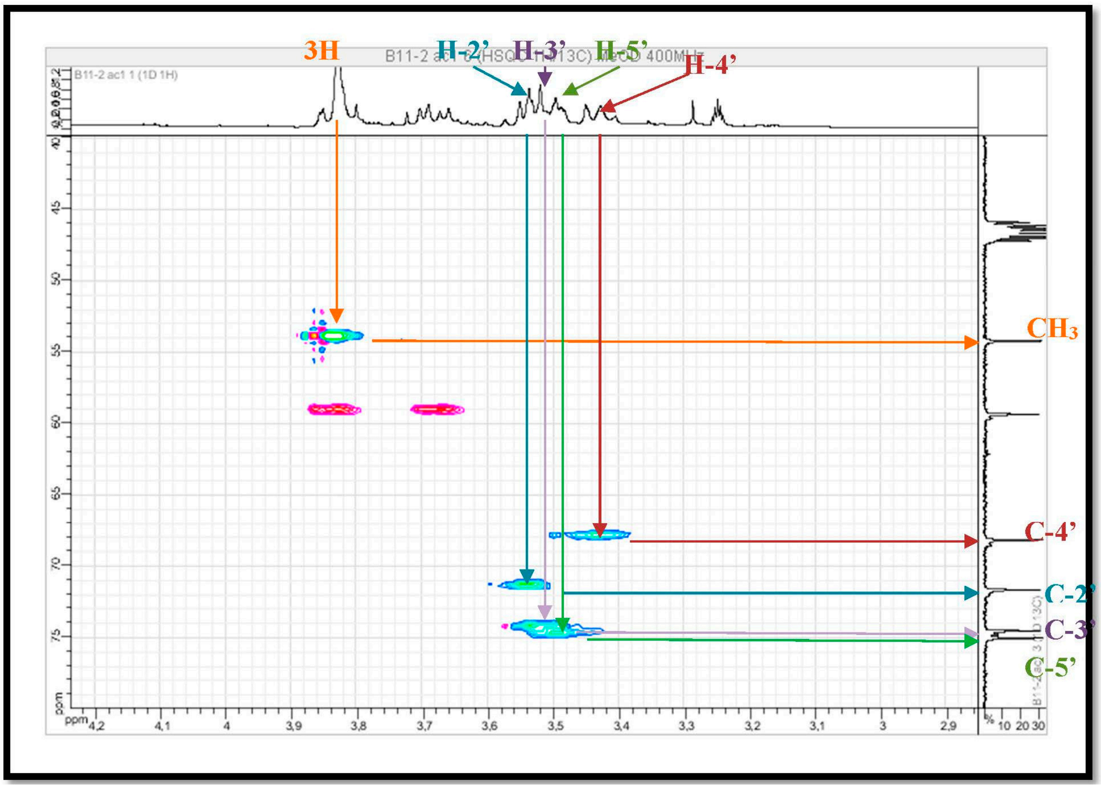

Figure S47. HSQC NMR spectrum (spreading out 1) (400 MHz, CD_3OD, δppm) of Vanillic acid 4-*O*-β-D-glucopyranoside.

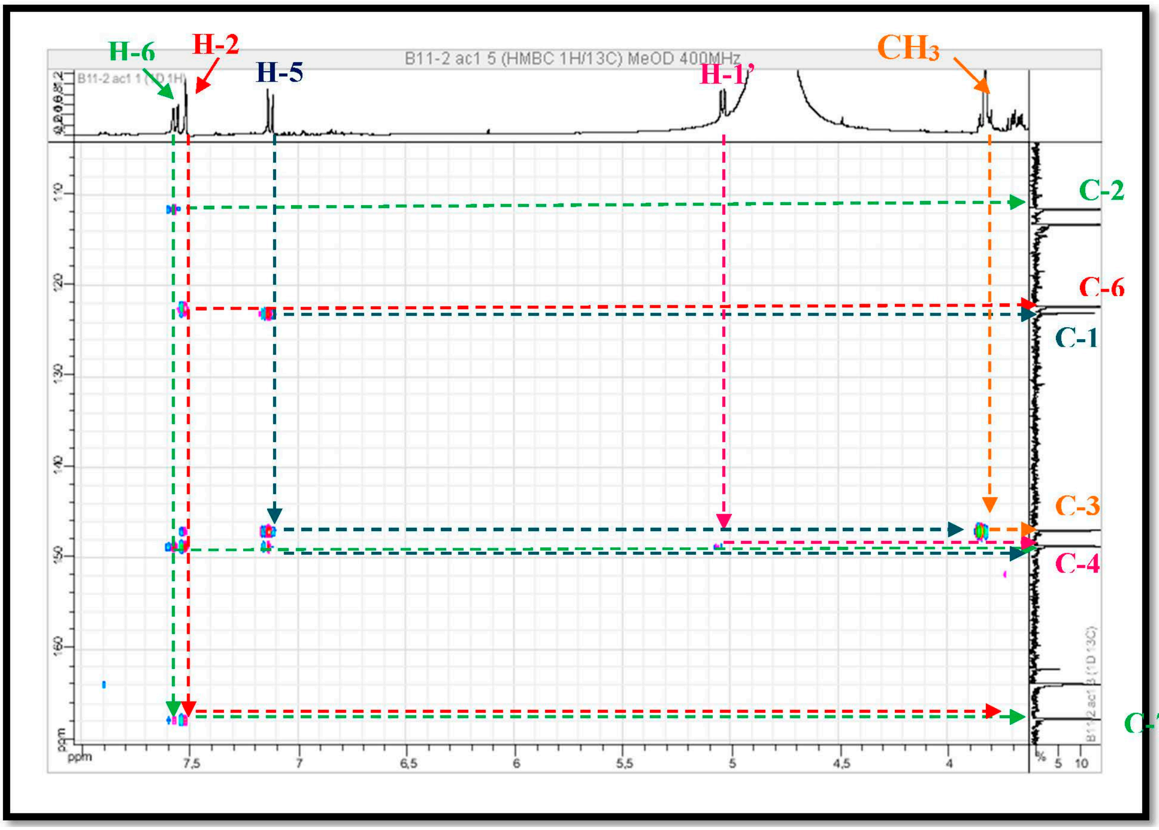

Figure S48. HMBC NMR spectrum (spreading out 1) (400 MHz, CD_3OD, δppm) of Vanillic acid 4-*O*-β-D-glucopyranoside.

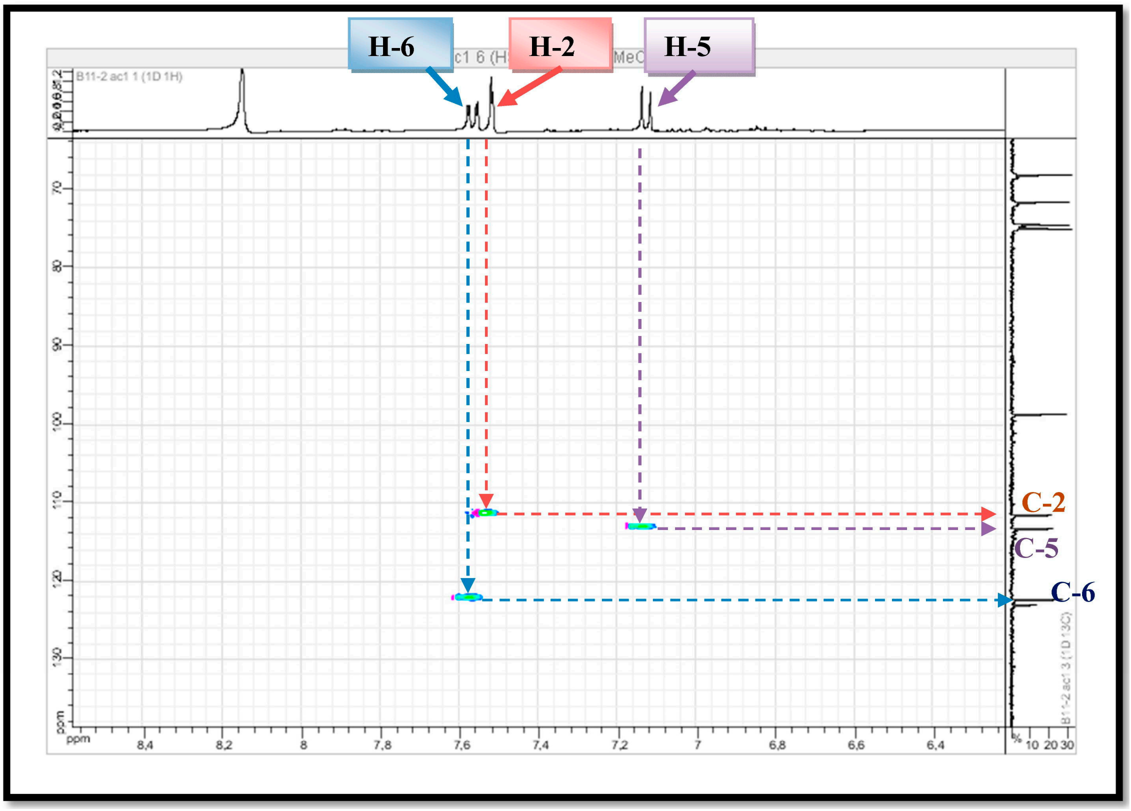

Figure S49. HSQC NMR spectrum (spreading out 2) (400 MHz, CD_3OD, δppm) of Vanillic acid 4-*O*-β-D-glucopyranoside.

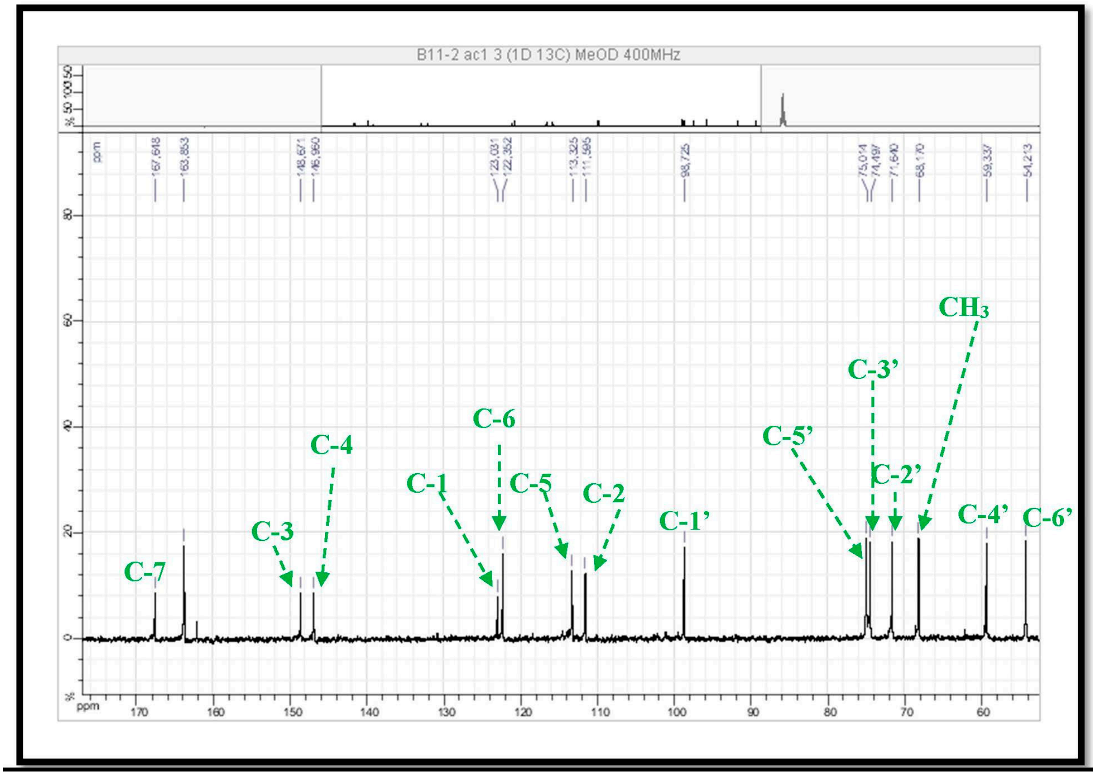

Figure S50. ^{13}C NMR spectrum (100 MHz, CD_3OD, δppm) of vanillic acid 4-*O*-β-D-glucopyranoside.

Molecule 9: Lavandoside

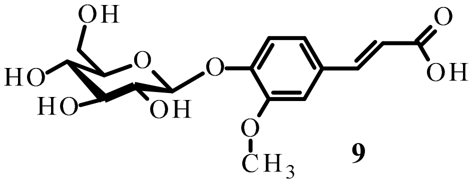

9

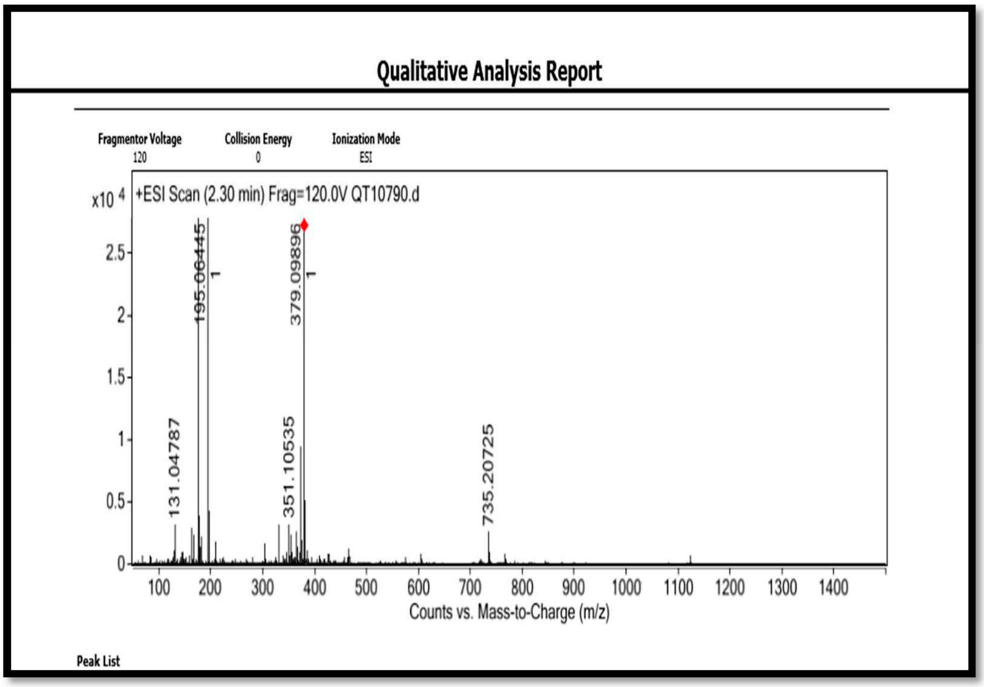

Figure S51. ESI-HRMS(+) of lavandoside.

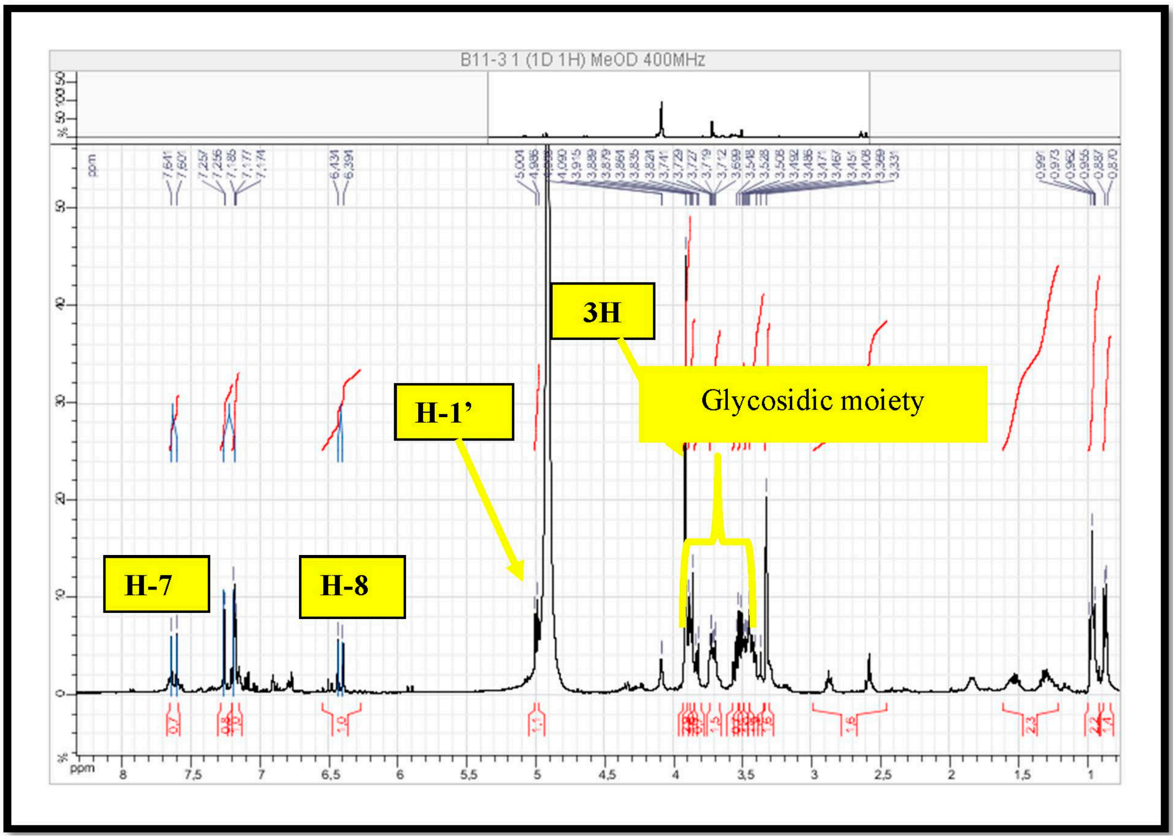

Figure S52. ^{1}H NMR spectrum (400 MHz, CD_3OD, δppm) of lavandoside.

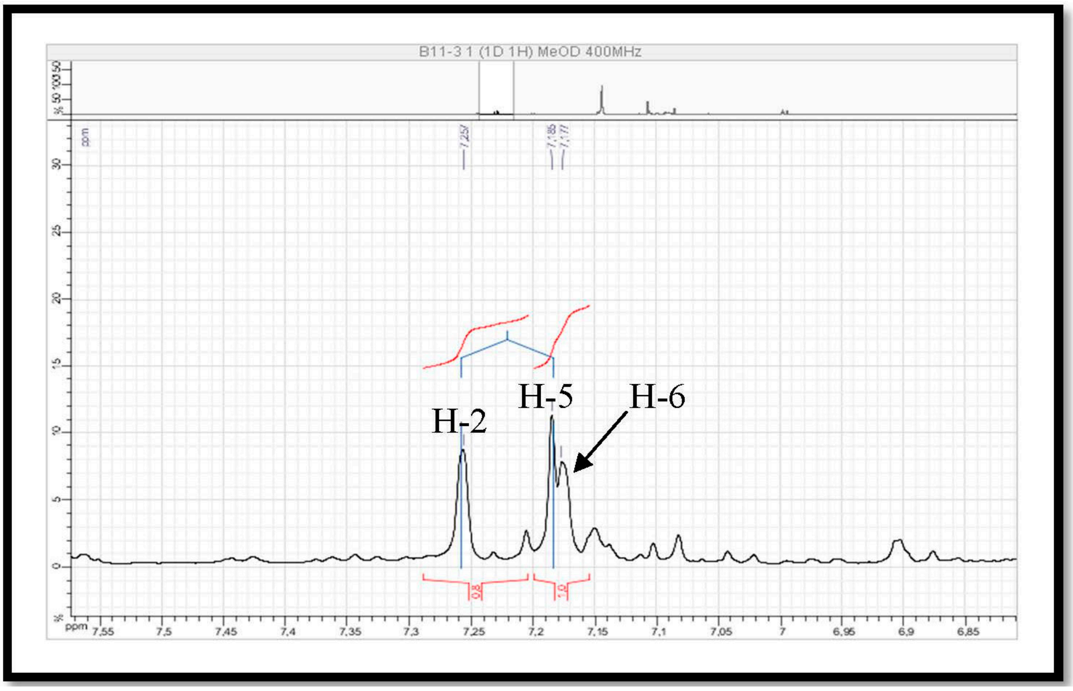

Figure S53. ^{1}H NMR spectrum (spreading out 1) (400 MHz, CD_3OD, δppm) of lavandoside.

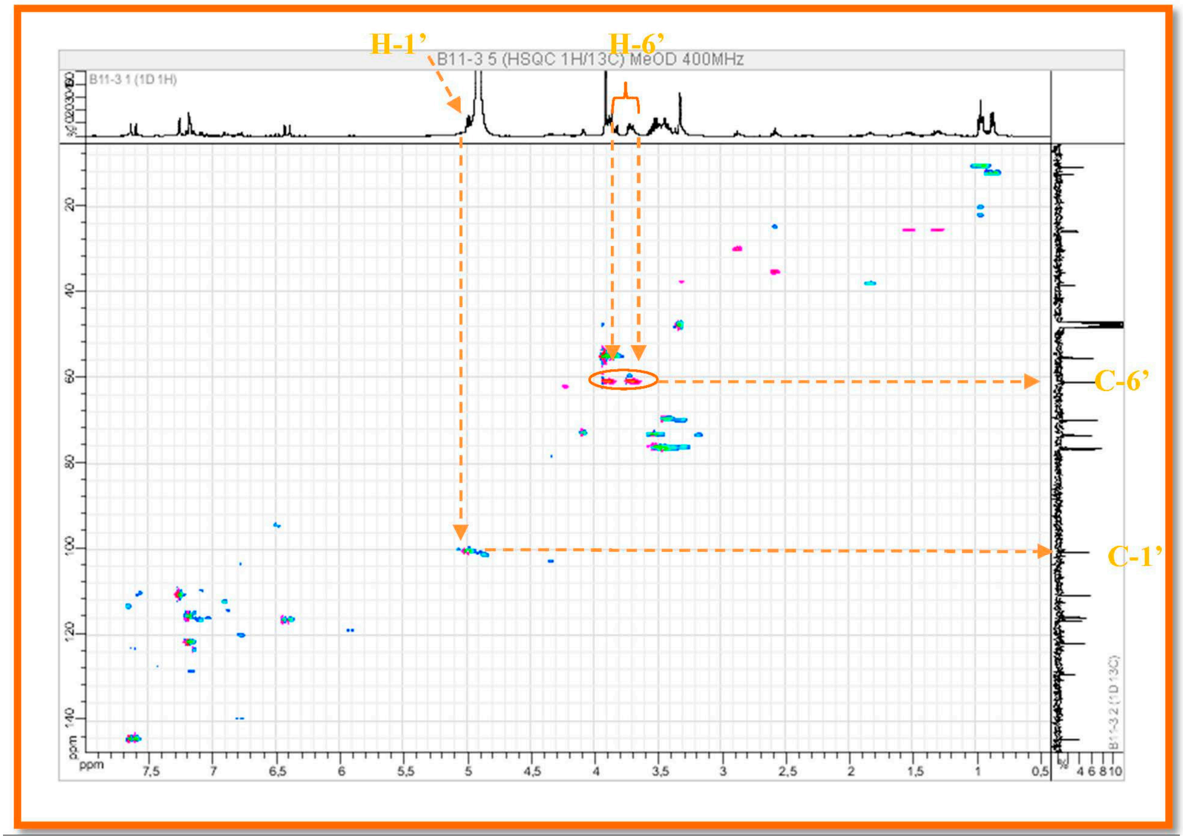

Figure S54. HSQC NMR spectrum (400 MHz, CD_3OD, δppm) of lavandoside.

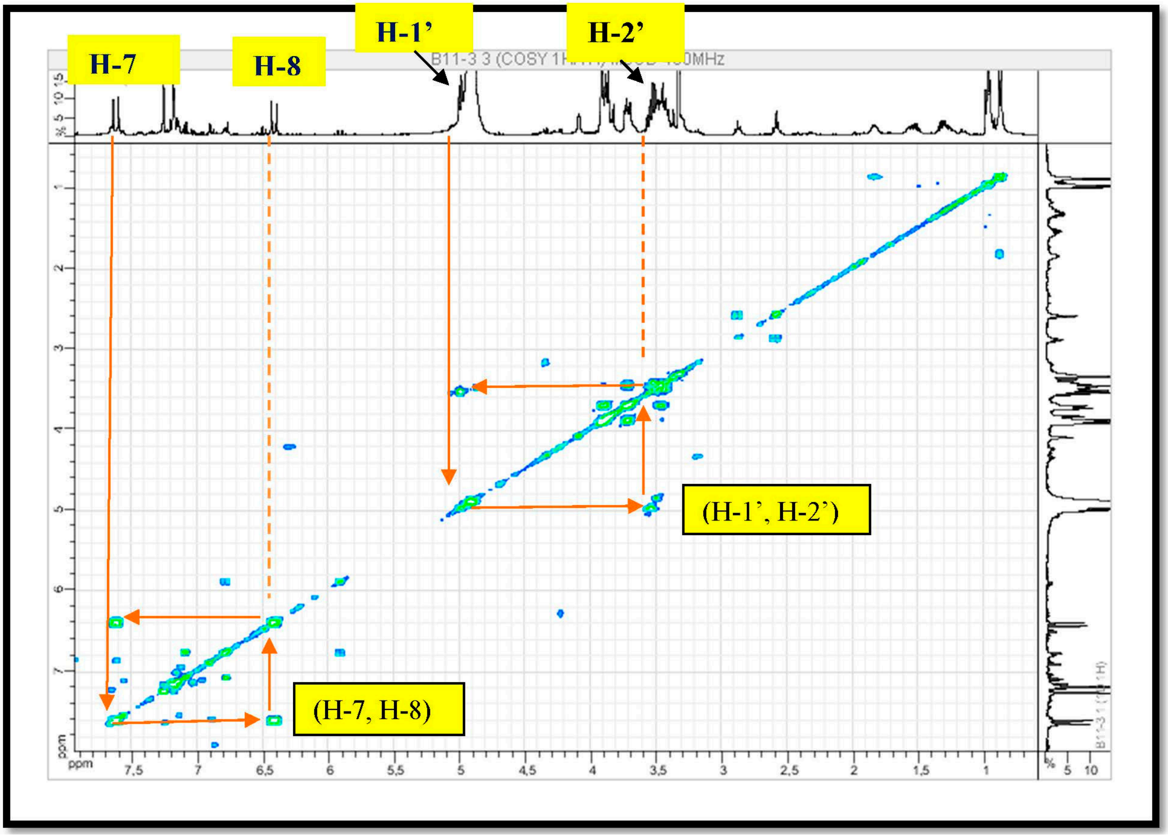

Figure S55. COSY NMR spectrum (400 MHz, CD_3OD, δppm) of lavandoside.

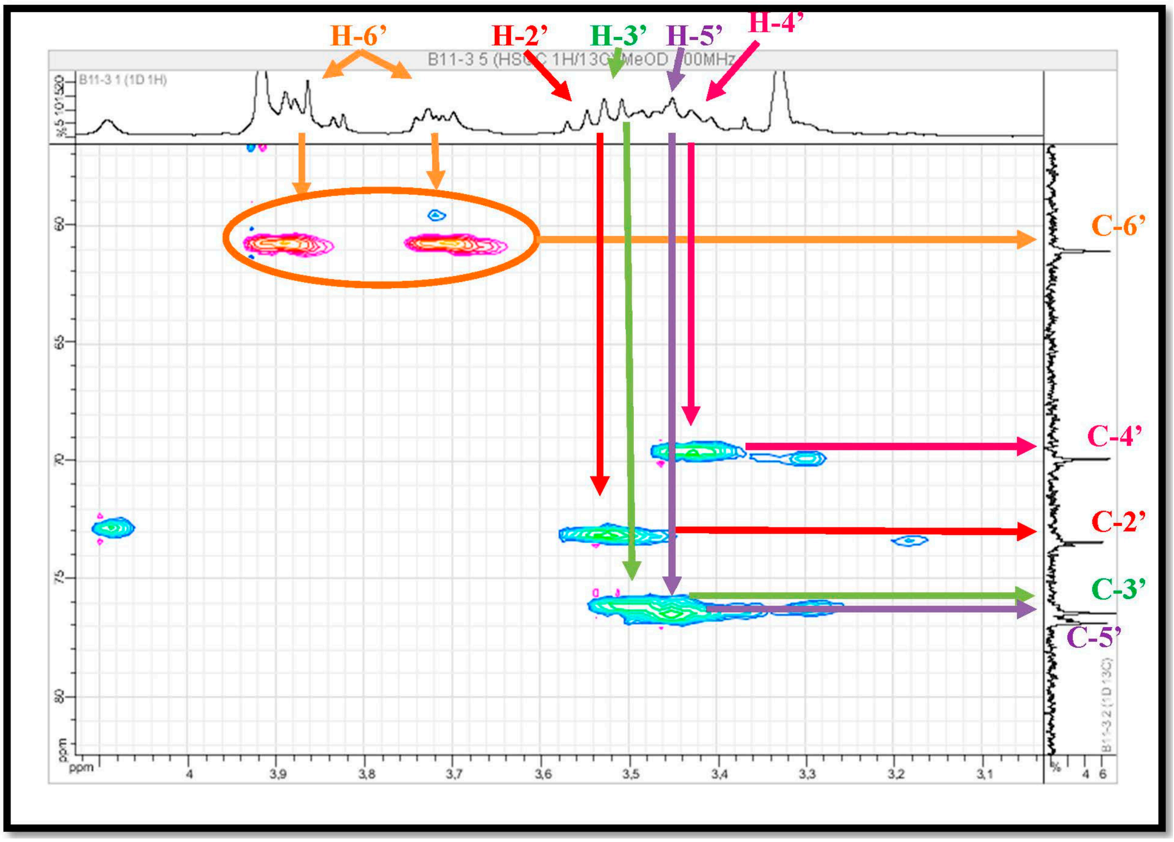

Figure S56. HSQC NMR spectrum (spreading out 1) (400 MHz, CD_3OD, δppm) of lavandoside.

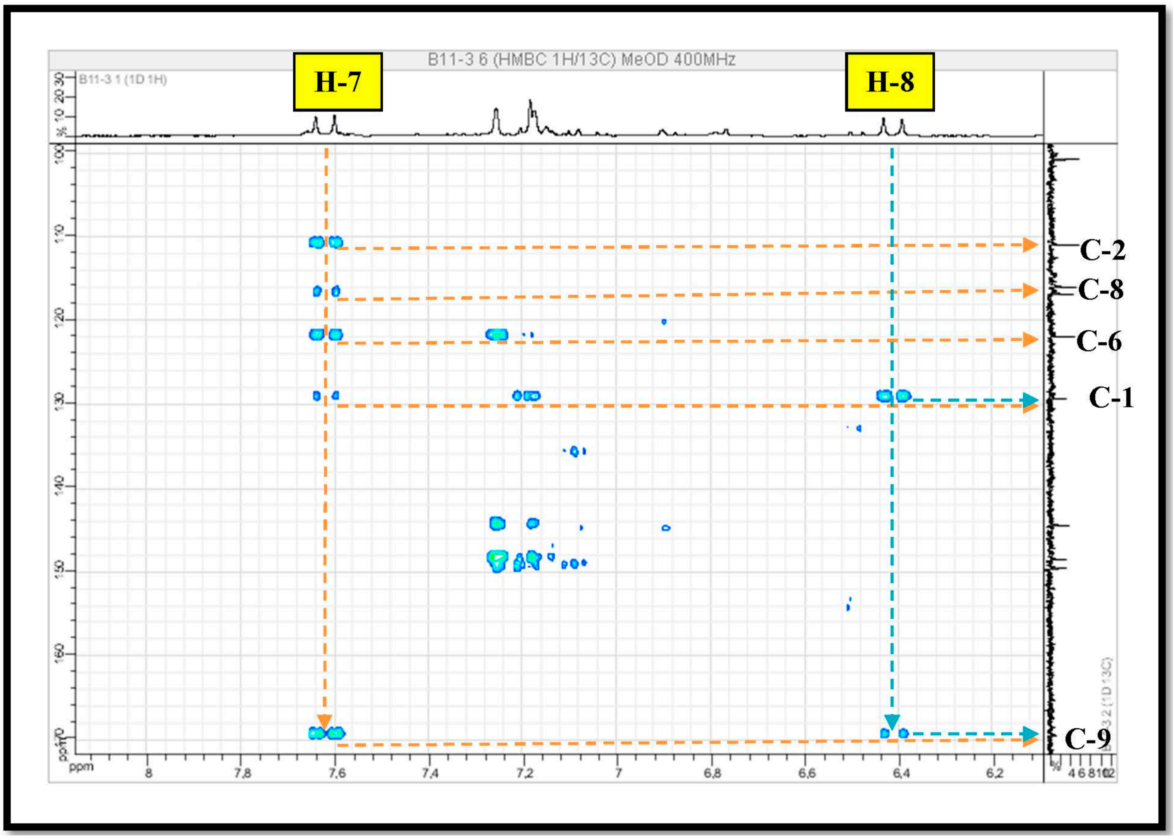

Figure S57. HMBC NMR spectrum (spreading out 1) (400 MHz, CD_3OD, δppm) of lavandoside.

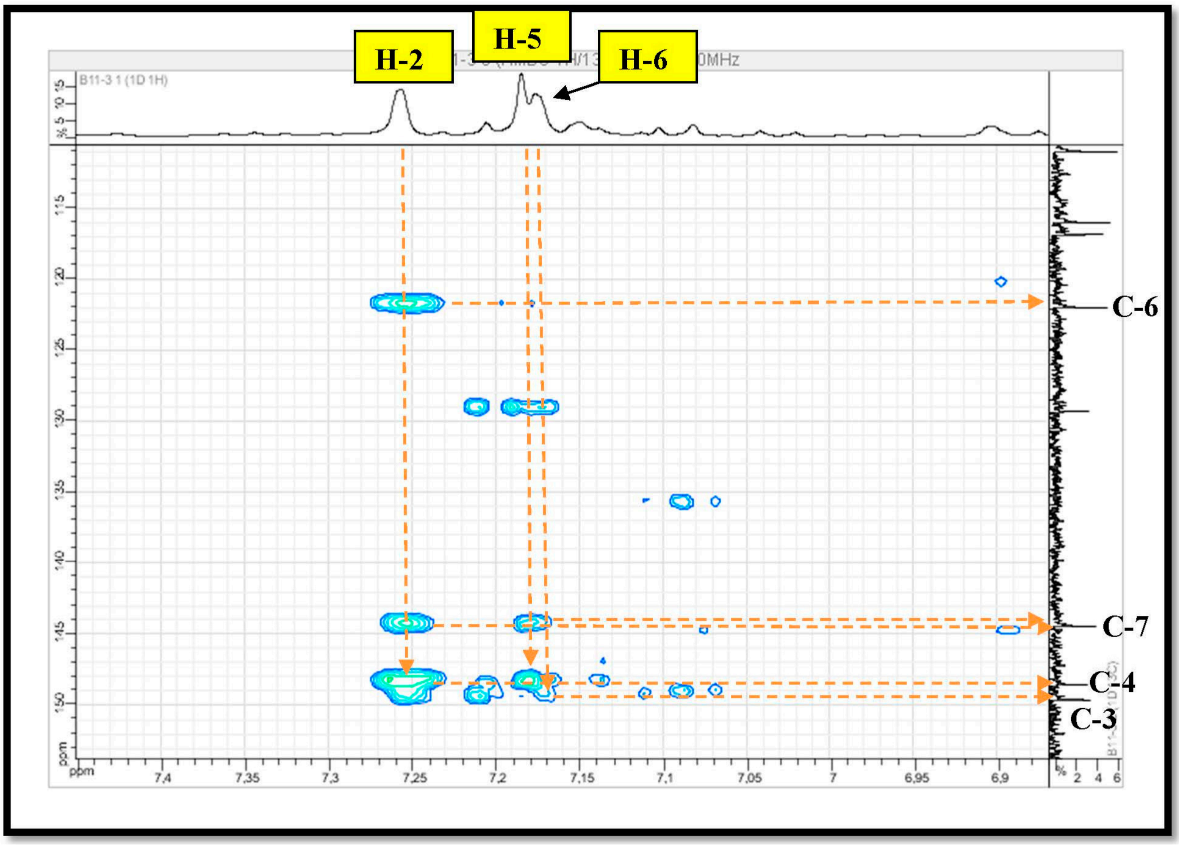

Figure S58. HMBC NMR spectrum (spreading out 2) (400 MHz, CD_3OD, δppm) of lavandoside.

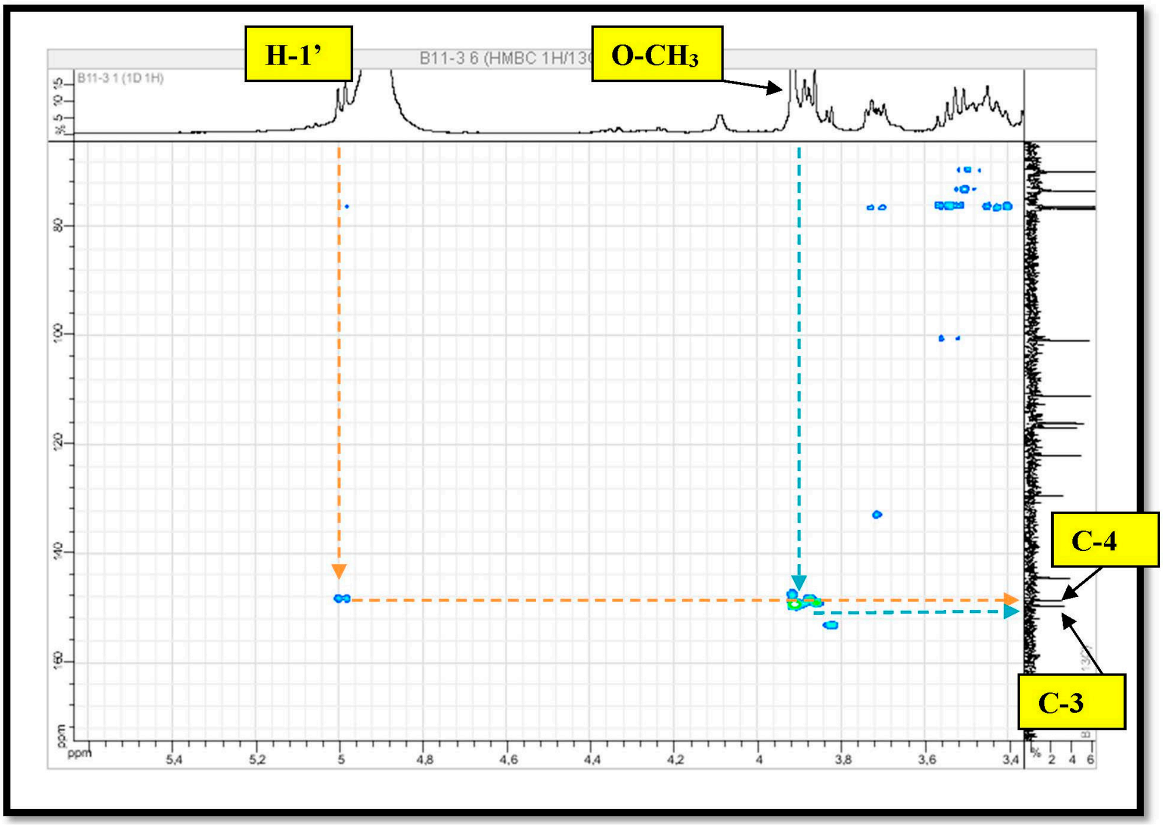

Figure S59. HMBC NMR spectrum (spreading out 3) (400 MHz, CD_3OD, δppm) of lavandoside.

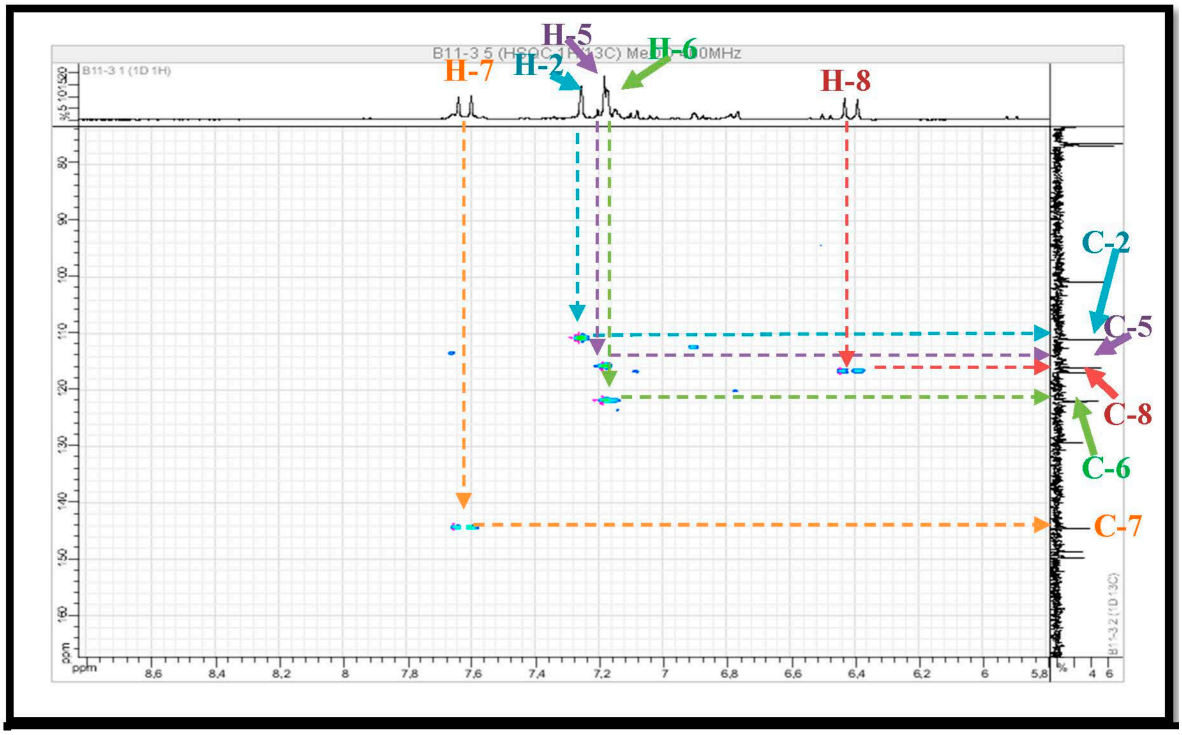

Figure S60. HSQC NMR spectrum (spreading out 2) (400 MHz, CD3OD, δppm) of lavandoside.

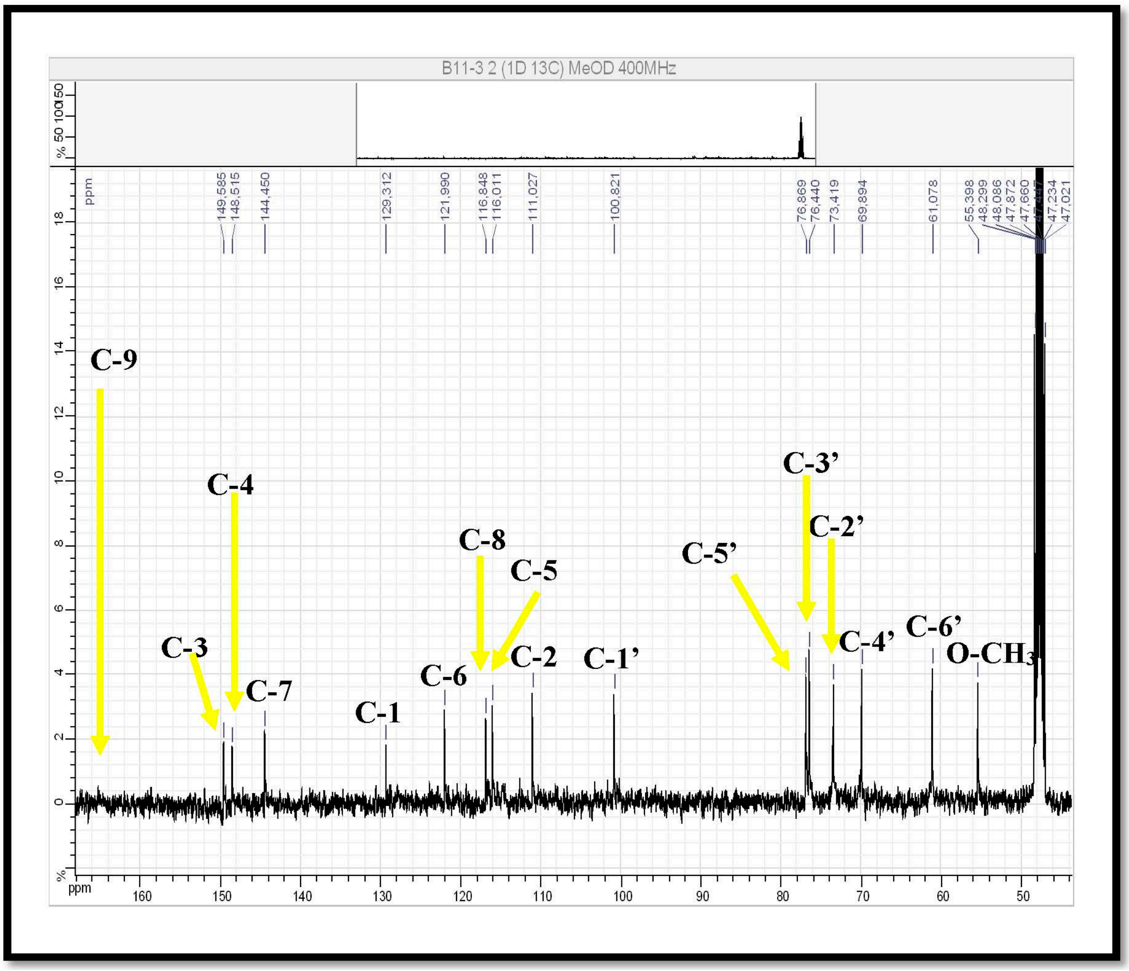

Figure S61. ^{13}C NMR spectrum (100 MHz, CD3OD, δppm) of lavandoside.

Molecule 10: 4-Hydroxy*benzoic acid* 4-*O*-β-D-glucopyranoside

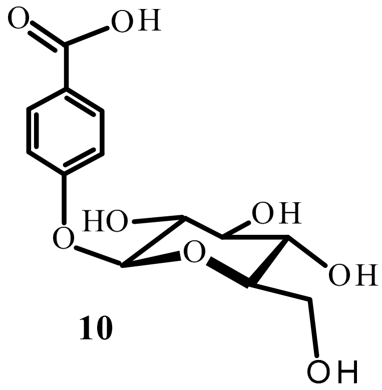

10

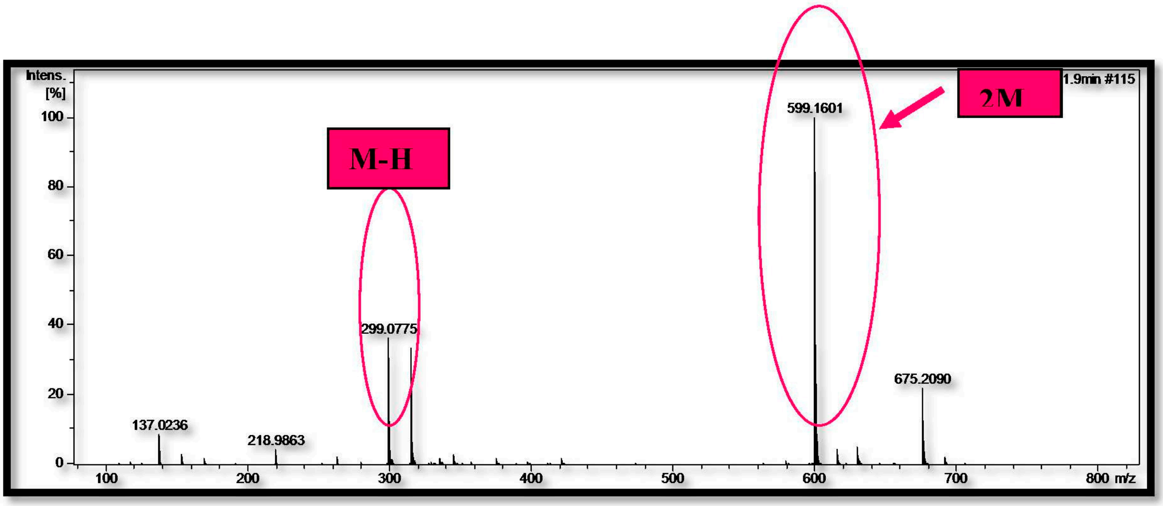

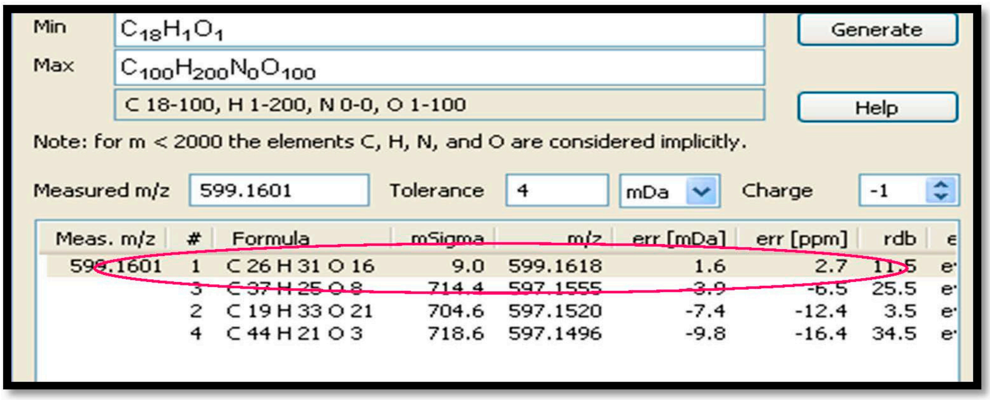

Meas. m/z	#	Formula	mSigma	m/z	err [mDa]	err [ppm]	rdb	e
599.1601	1	C 26 H 31 O 16	9.0	599.1618	1.6	2.7	11.5	e
	3	C 37 H 25 O 8	714.4	597.1555	-3.9	-6.5	25.5	e
	2	C 19 H 33 O 21	704.6	597.1520	-7.4	-12.4	3.5	e
	4	C 44 H 21 O 3	718.6	597.1496	-9.8	-16.4	34.5	e

Figure S62. ESI-HRMS(−) of 4-hydroxybenzoic acid 4-*O*-β-D-glucopyranoside.

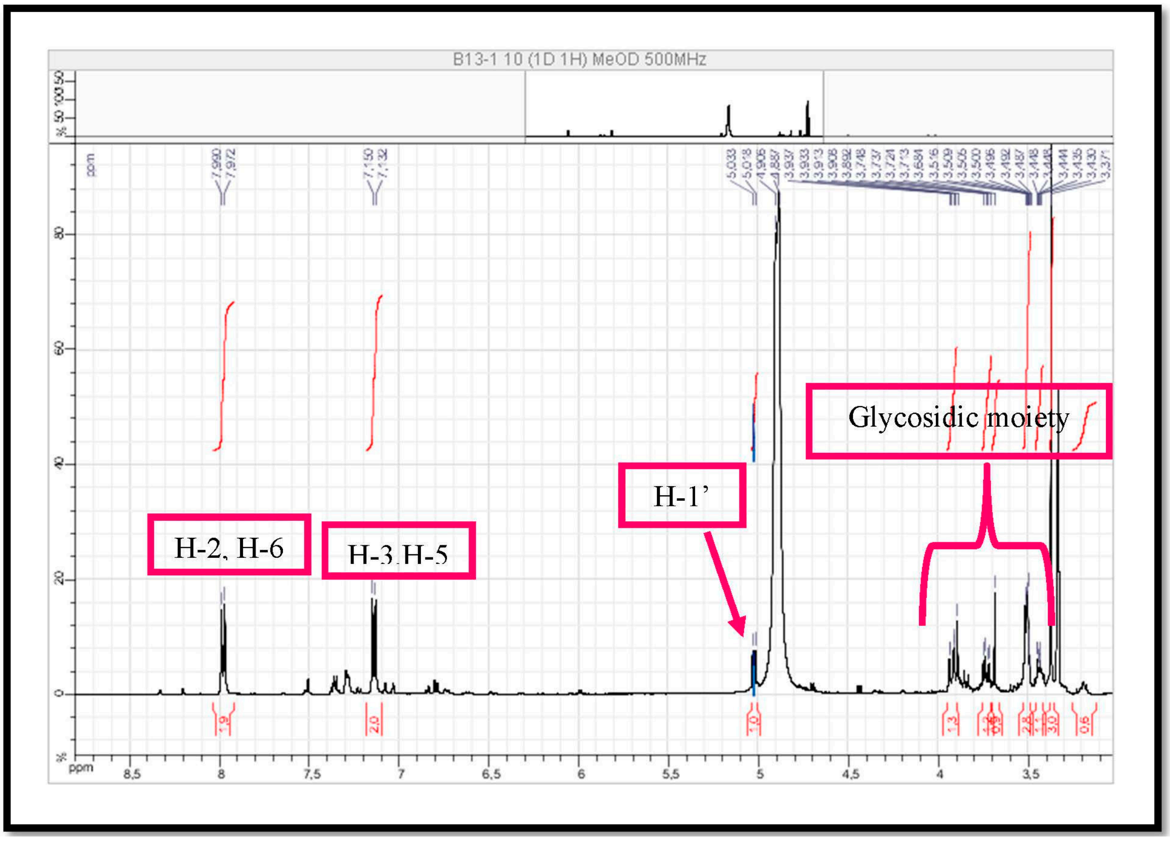

Figure S63. ^{1}H NMR spectrum (500 MHz, CD_3OD, δppm) of 4-hydroxybenzoic acid 4-*O*-β-D-glucopyranoside.

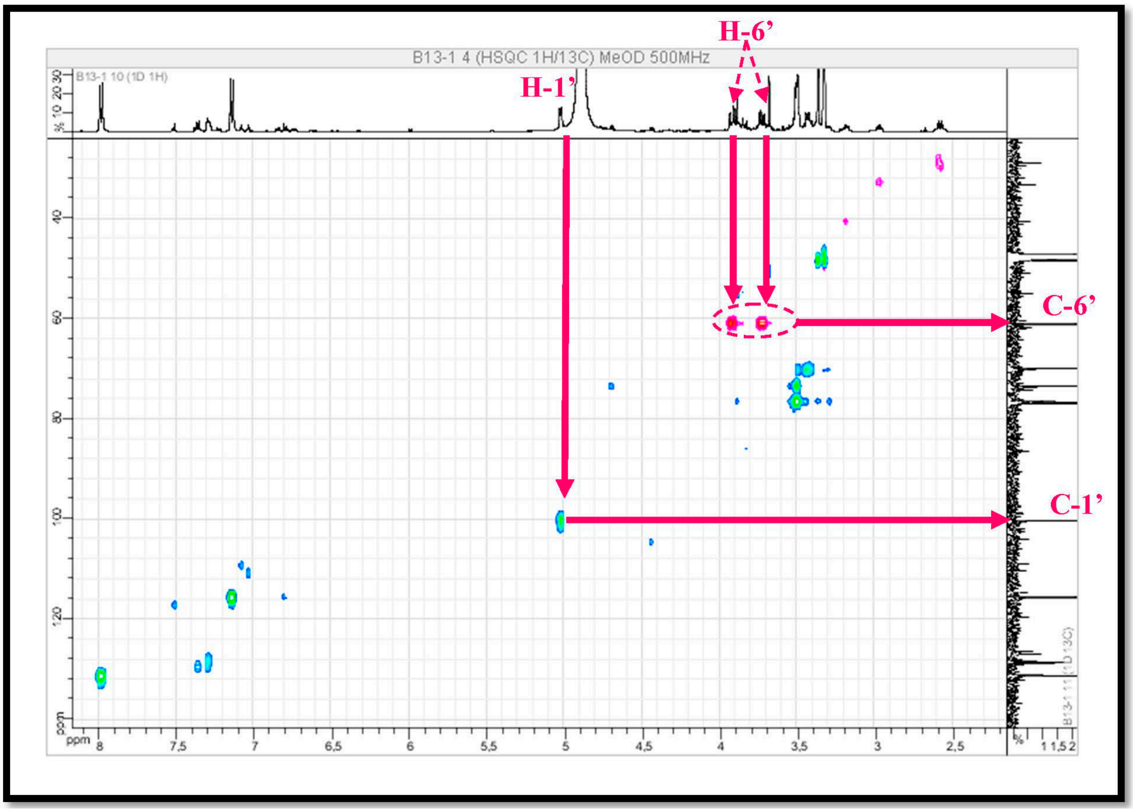

Figure S64. HSQC NMR spectrum (500 MHz, CD3OD, δppm) of 4-hydroxybenzoic acid 4-*O*-β-D-glucopyranoside.

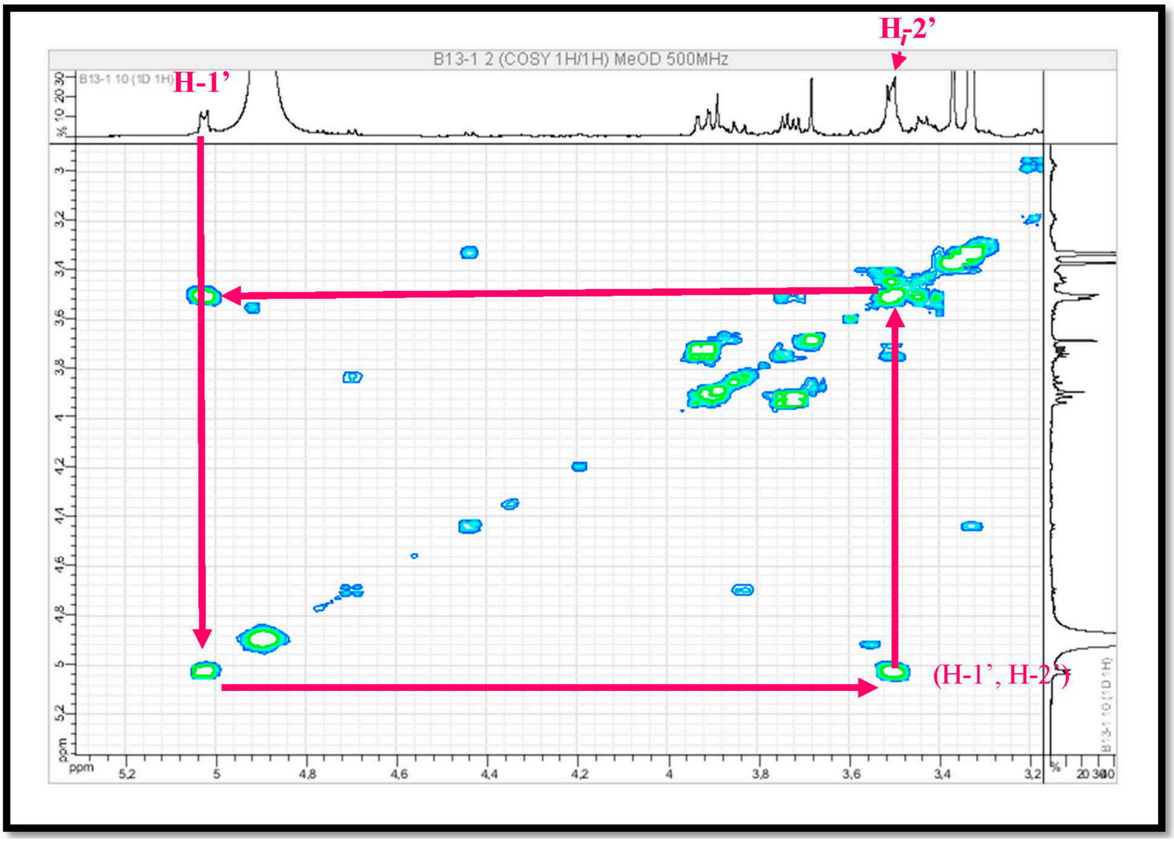

Figure S65. COSY NMR spectrum (spreading out 1) (500 MHz, CD_3OD, δppm) of 4-hydroxybenzoic acid 4-*O*-β-D-glucopyranoside.

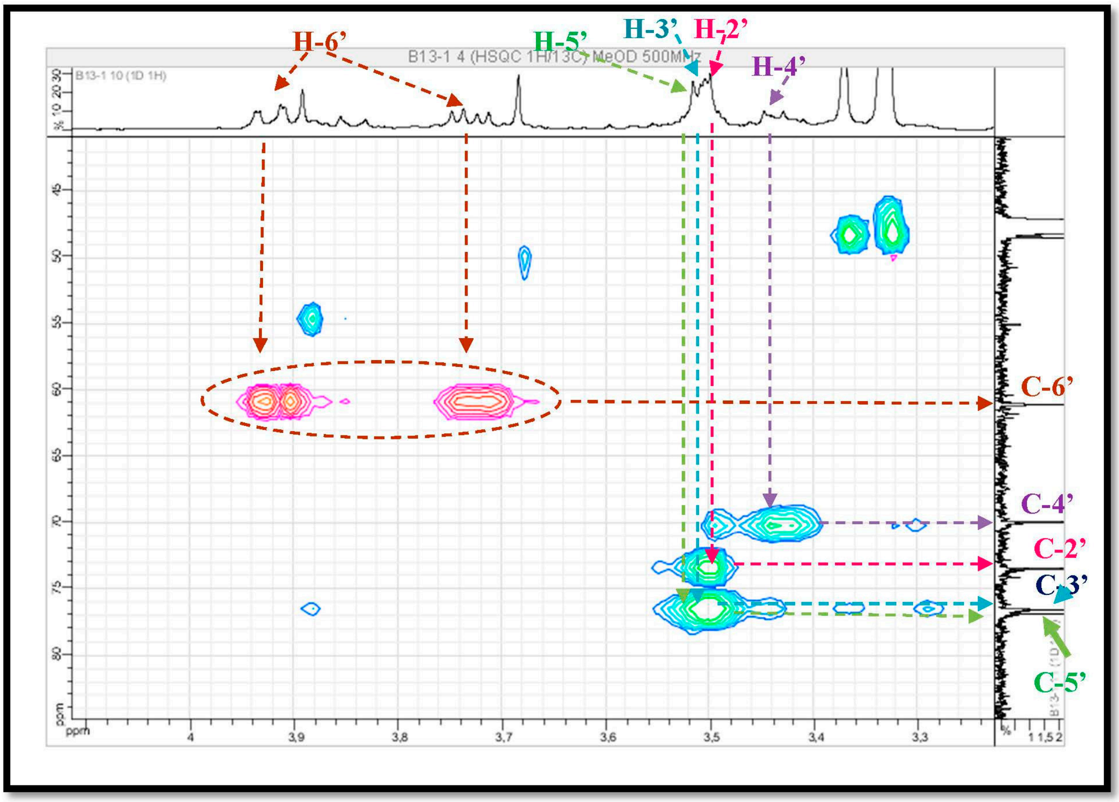

Figure S66. HSQC NMR spectrum (spreading out 1) (500 MHz, CD_3OD, δppm) of 4-hydroxybenzoic acid 4-O-β-D-glucopyranoside.

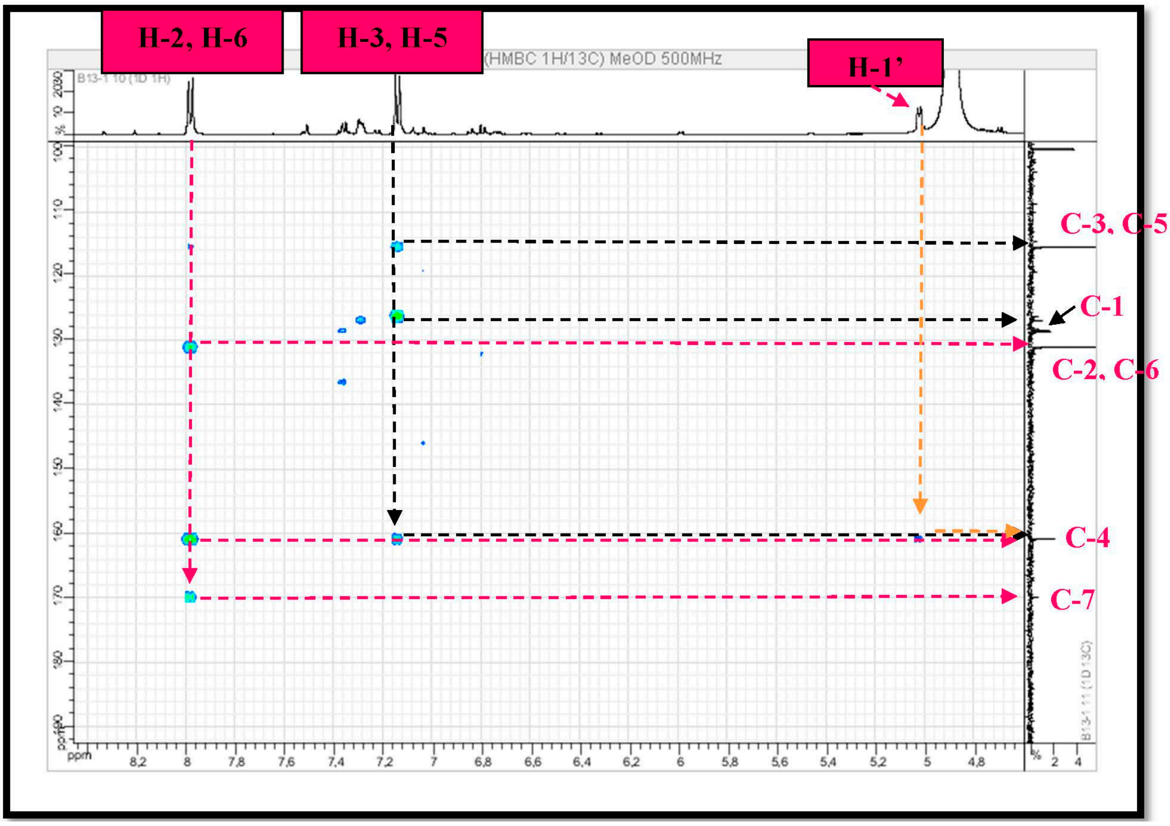

Figure S67. HMBCNMR spectrum (spreading out 1) (500 MHz, CD_3OD, δppm) of 4-hydroxybenzoic acid 4-*O*-β-D-glucopyranoside.

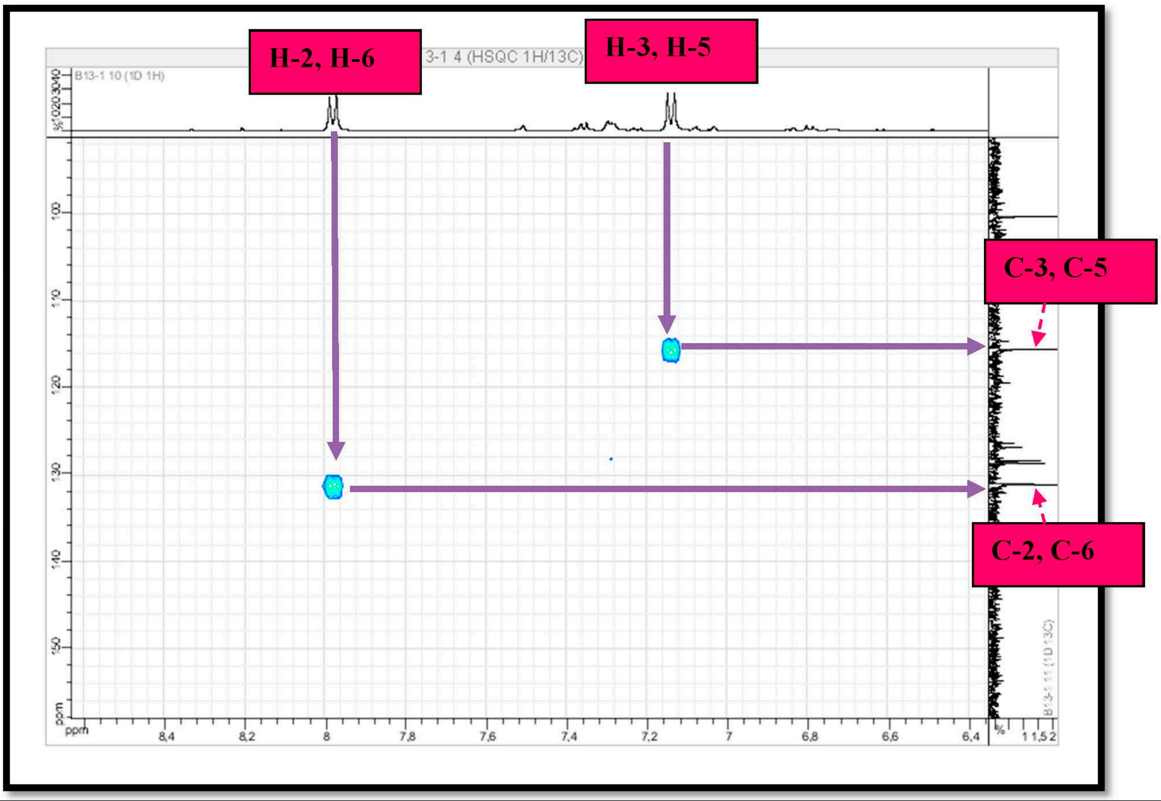

Figure S68. HSQC NMR spectrum (spreading out 2) (500 MHz, CD_3OD, δppm) of 4-hydroxybenzoic acid 4-*O*-β-D-glucopyranoside.

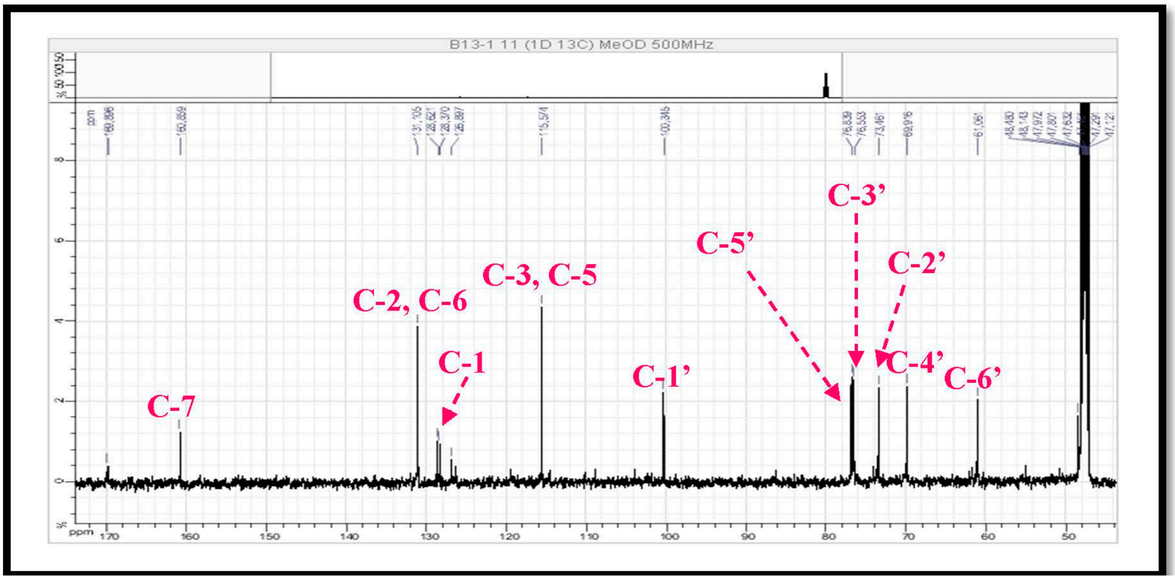

Figure S69. HSQC NMR spectrum (125 MHz, CD_3OD, δppm) of 4-hydroxybenzoic acid 4-*O*-β-D-glucopyranoside.

Molecule 11: Nicotiflorin

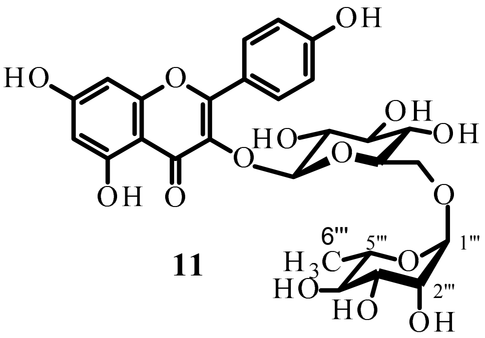

11

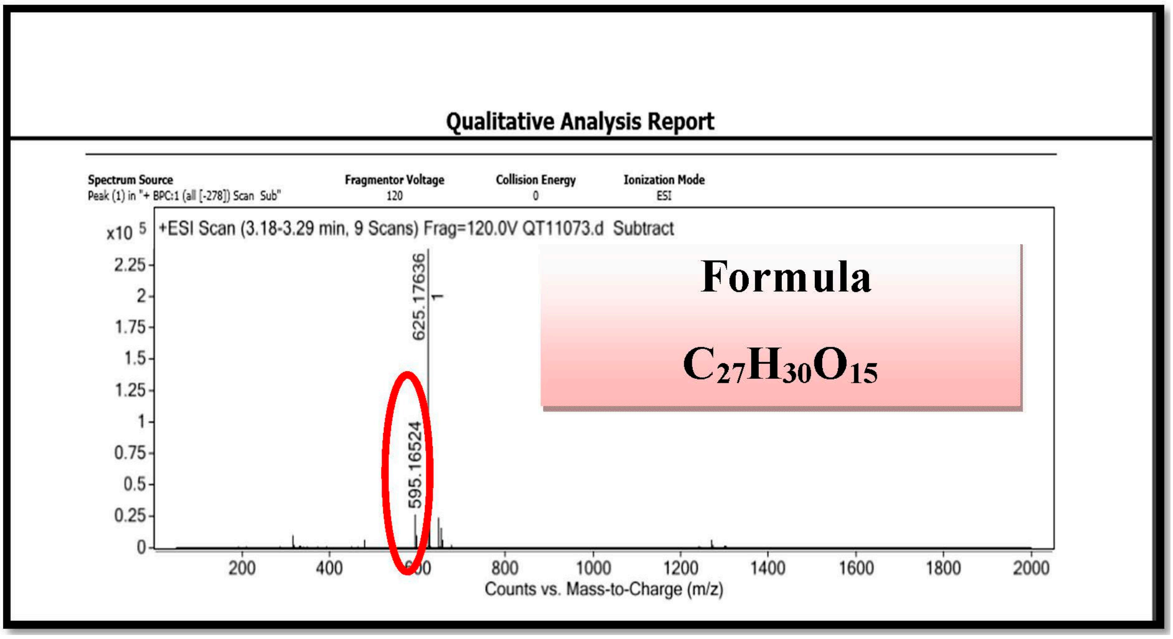

Figure S70. ESI-HRMS(+) of nicotiflorin.

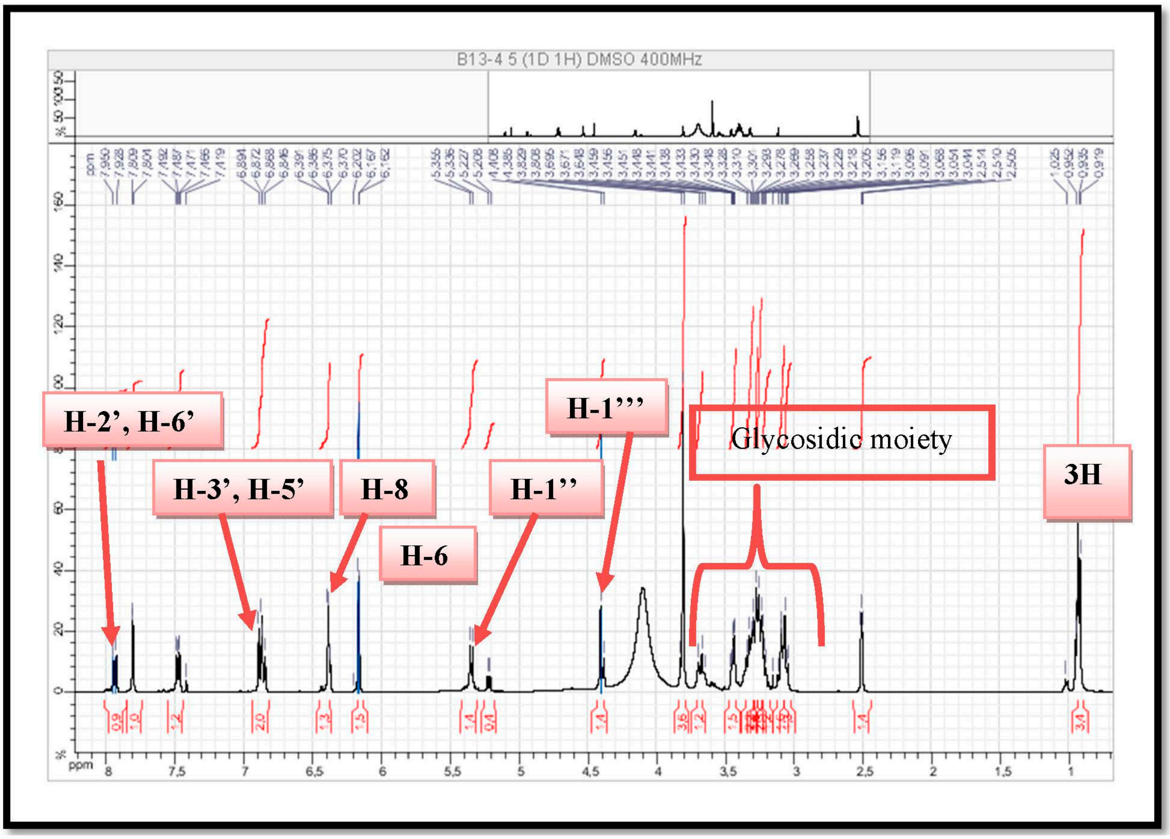

Figure S71. ^{1}H NMR spectrum (400 MHz, DMSO-d_6, δppm) of nicotiflorin.

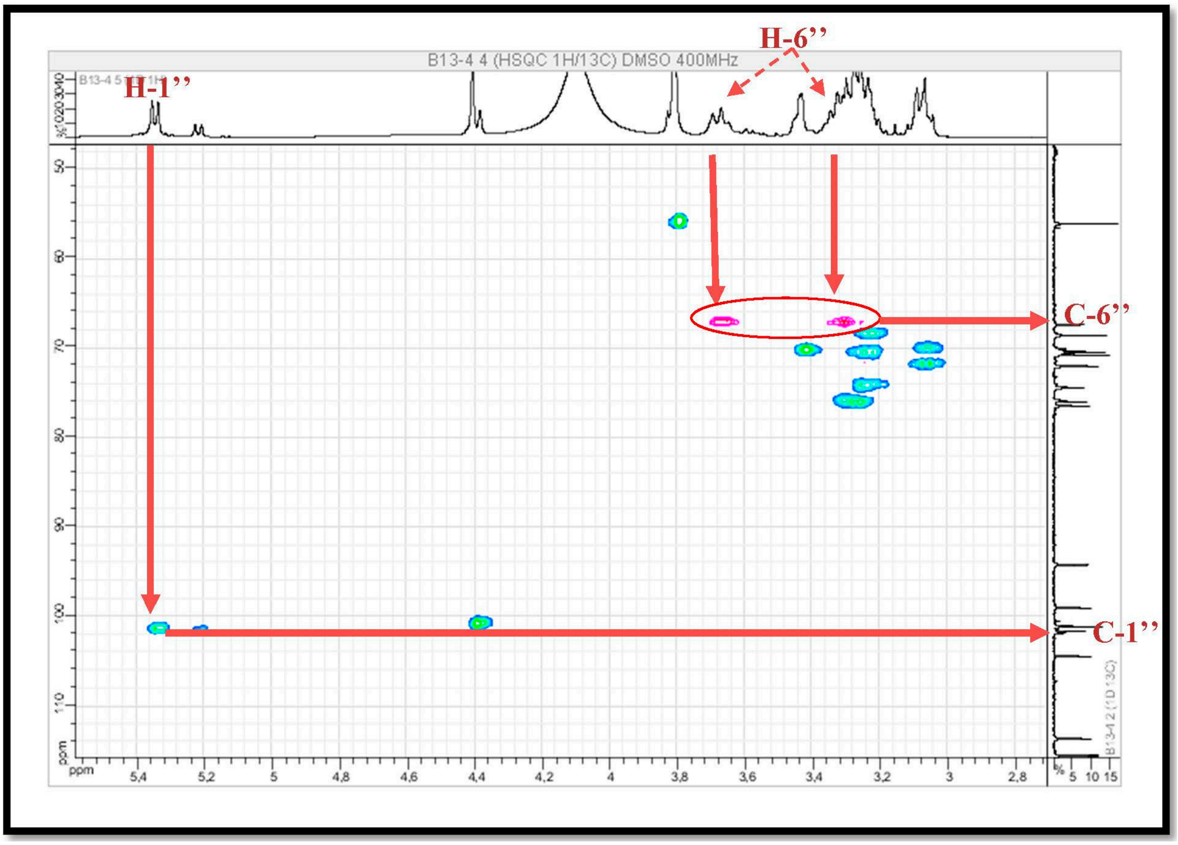

Figure S72. HSQC NMR spectrum (spreading out 1) (400 MHz, DMSO-d_6, δppm) of nicotiflorin.

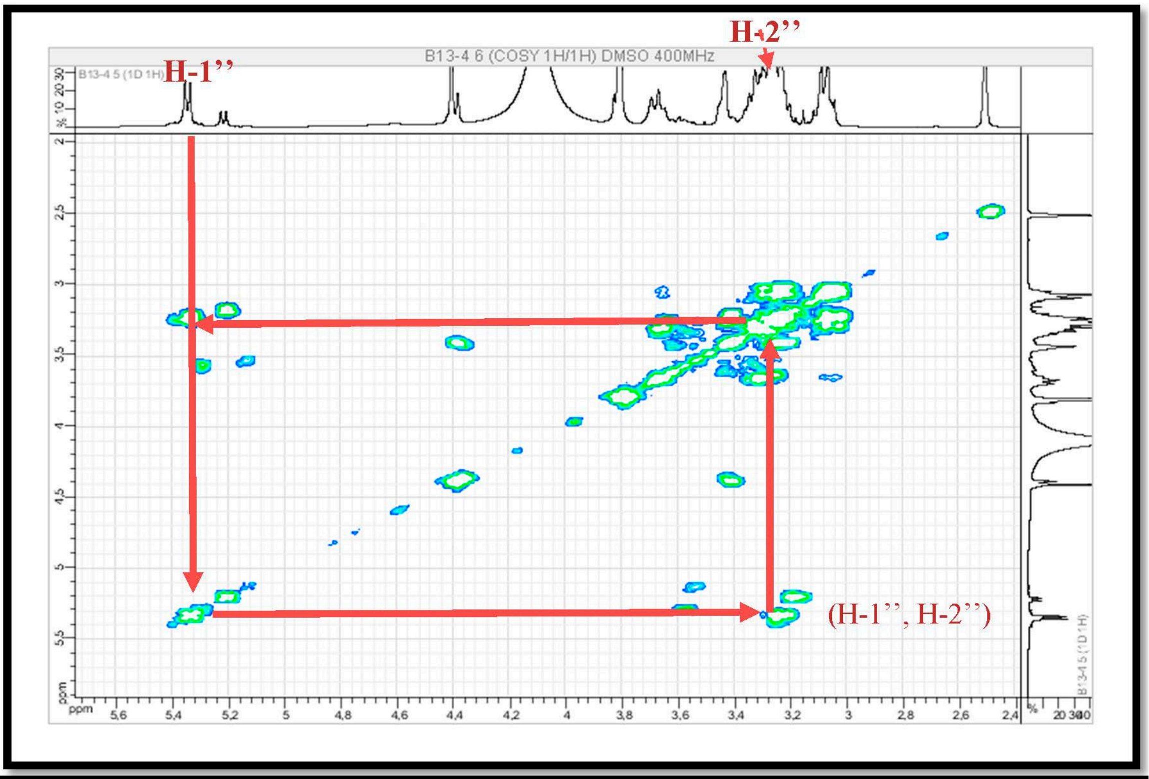

Figure S73. COSY NMR spectrum (spreading out 1) (400 MHz, DMSO-d_6, δppm) of nicotiflorin.

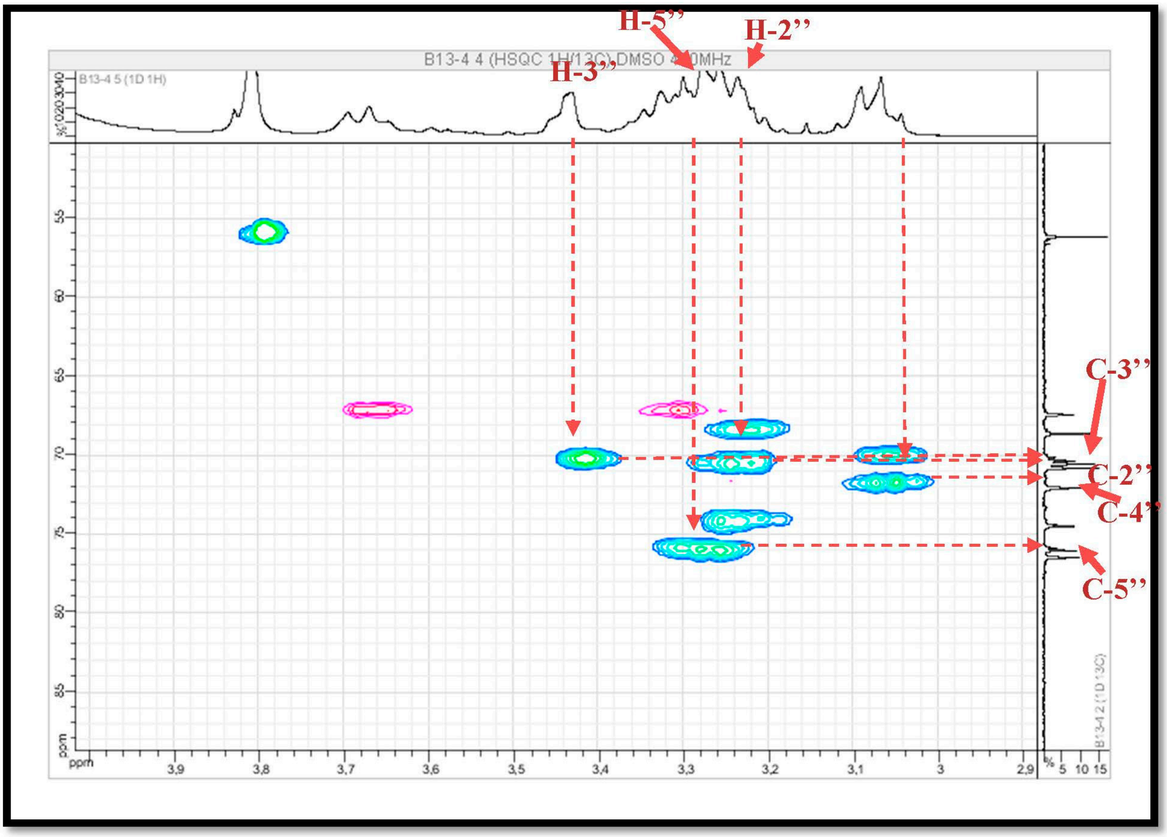

Figure S74. HSQC NMR spectrum (spreading out 2) (400 MHz, DMSO-d_6, δppm) of nicotiflorin.

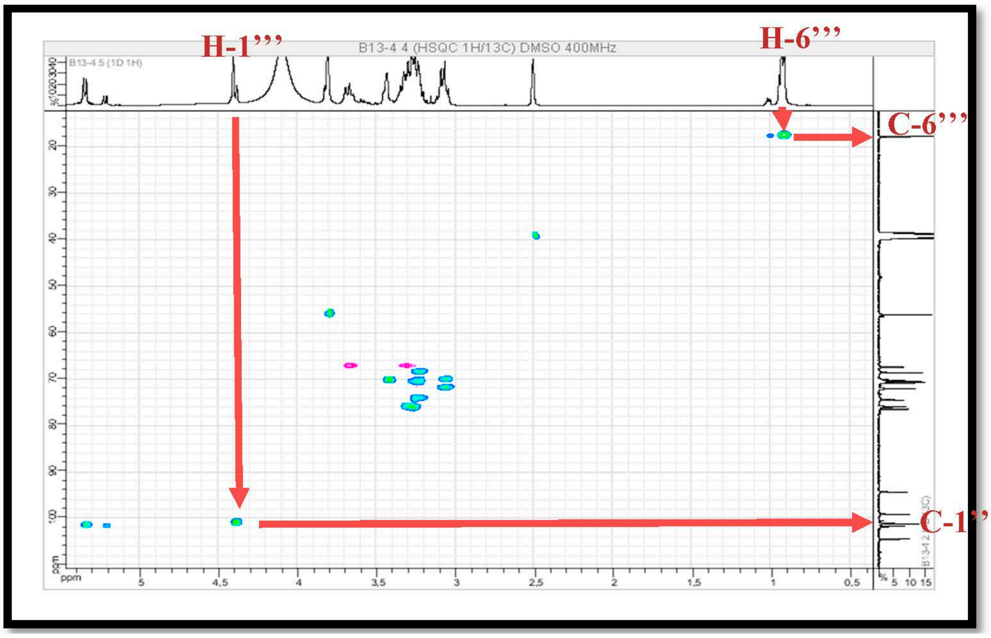

Figure S75. HSQC NMR spectrum (spreading out 3) (400 MHz, DMSO-d_6, δppm) of nicotiflorin.

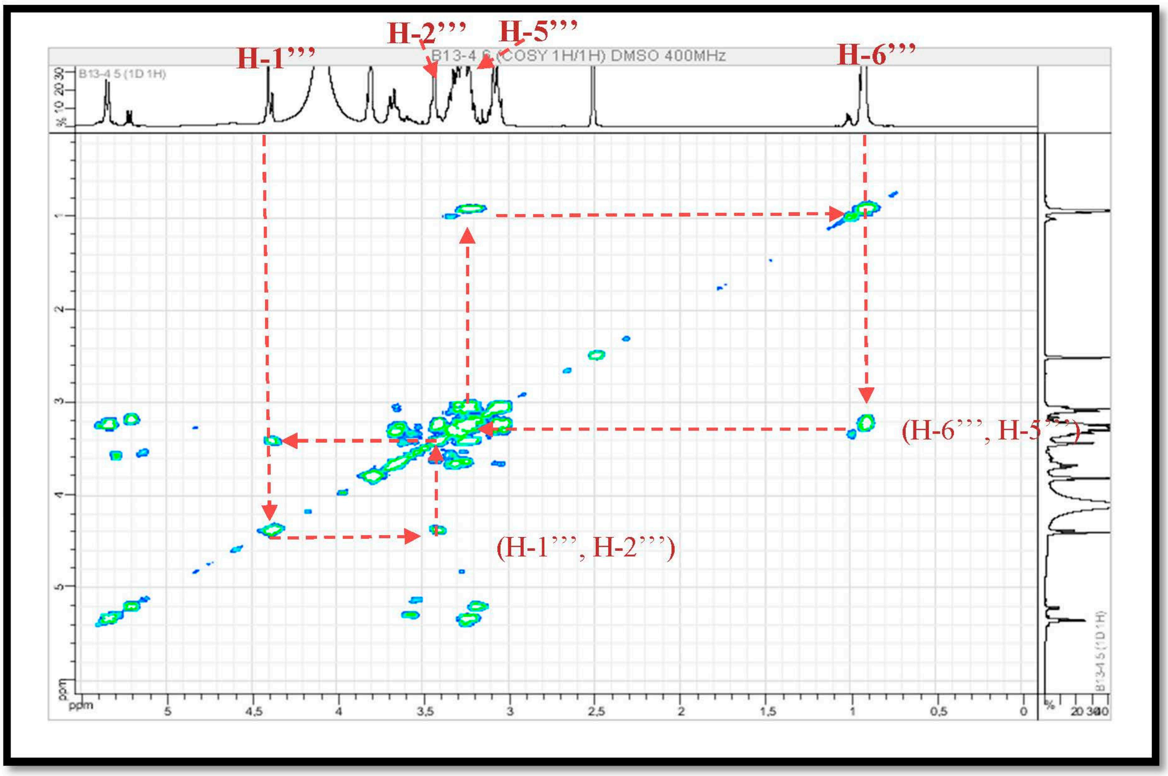

Figure S76. COSY NMR spectrum (spreading out 2) (400 MHz, DMSO-d_6, δppm) of nicotiflorin.

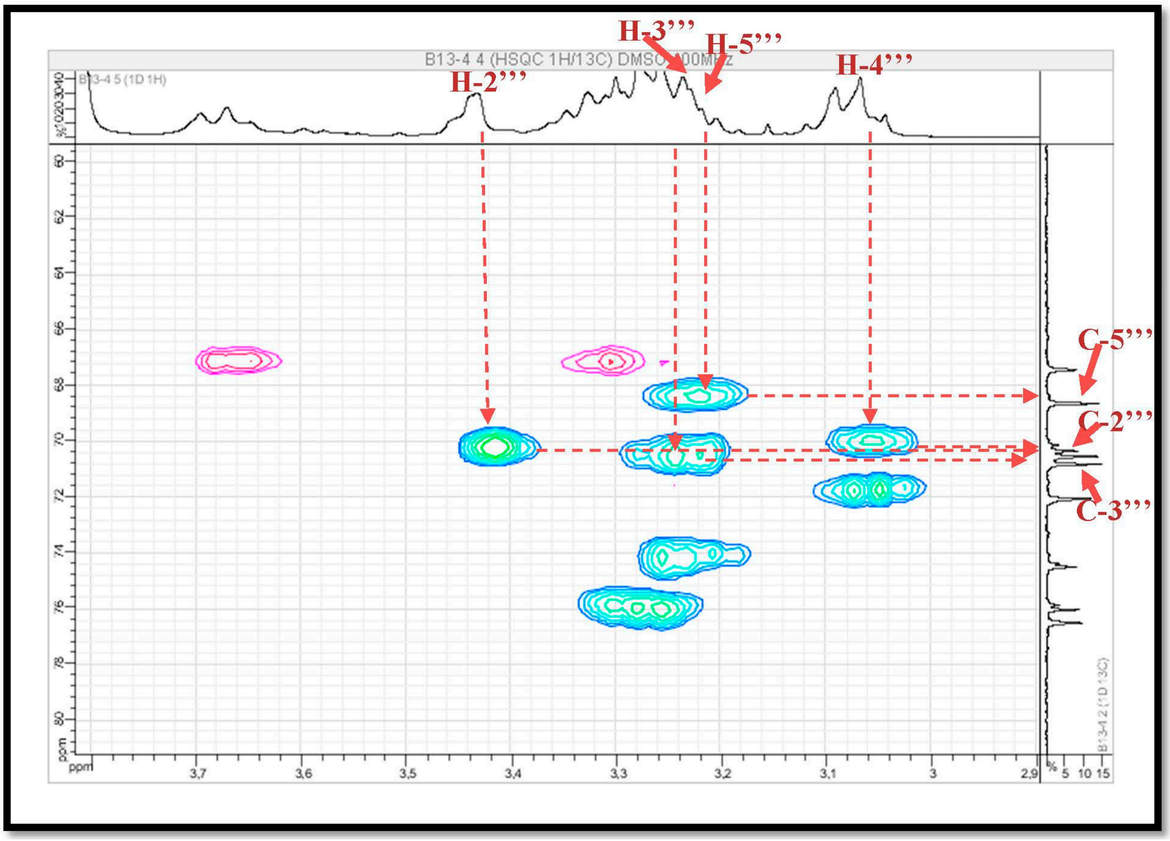

Figure S77. HSQC NMR spectrum (spreading out 4) (400 MHz, DMSO-d_6, δppm) of nicotiflorin.

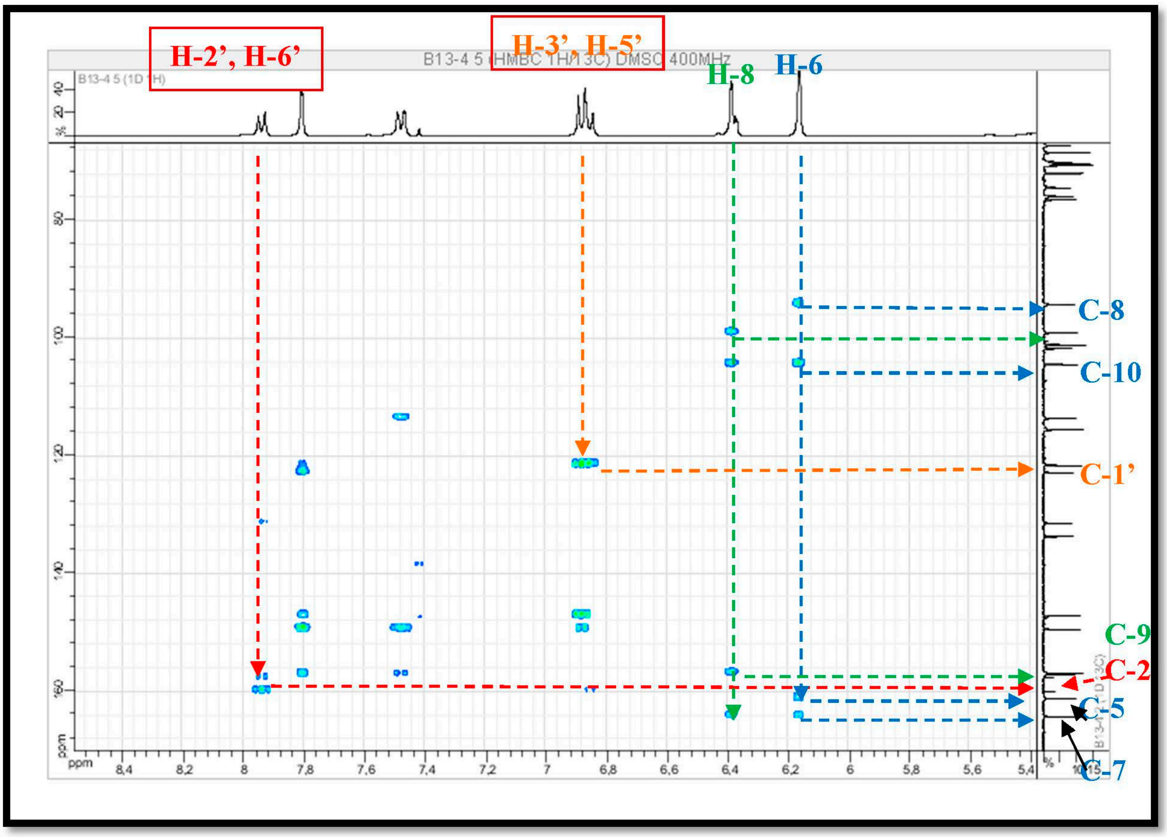

Figure S78. HMBC NMR spectrum (400 MHz, DMSO-d_6, δppm) of nicotiflorin.

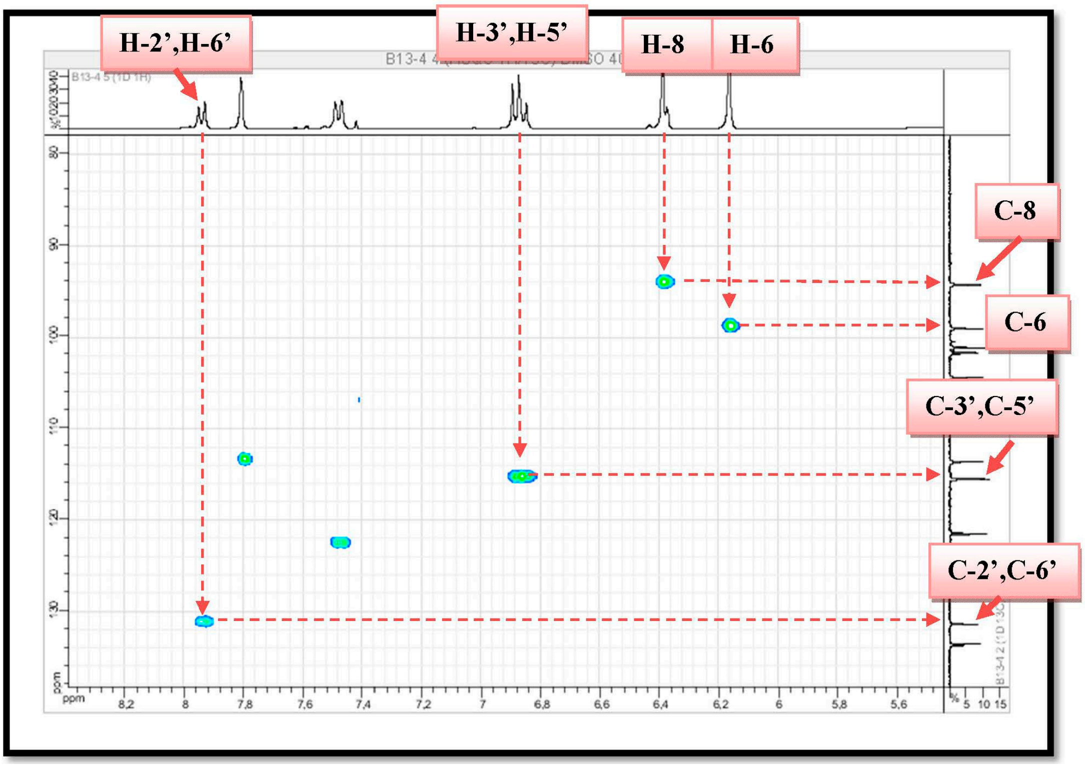

Figure S79. HSQC NMR spectrum (spreading out 5) (400 MHz, DMSO-d_6, δppm) of nicotiflorin.

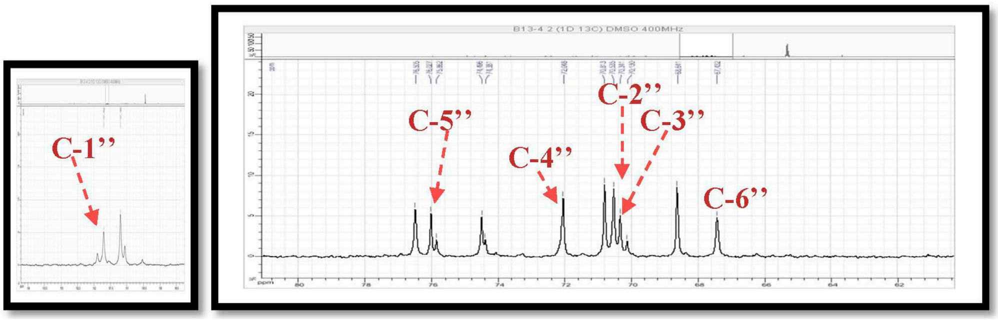

Figure S80. ^{13}C NMR spectrum (spreading out 1) (100 MHz, DMSO-d_6, δppm) of nicotiflorin.

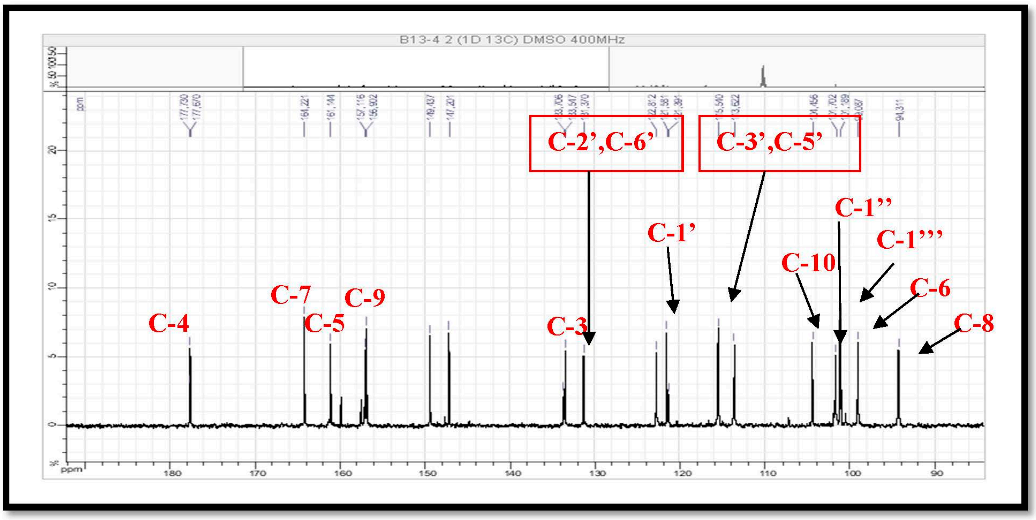

Figure S81. ^{13}C NMR spectrum (100 MHz, DMSO-d_6, δppm) of nicotiflorin.

Molecule 12: Rutin

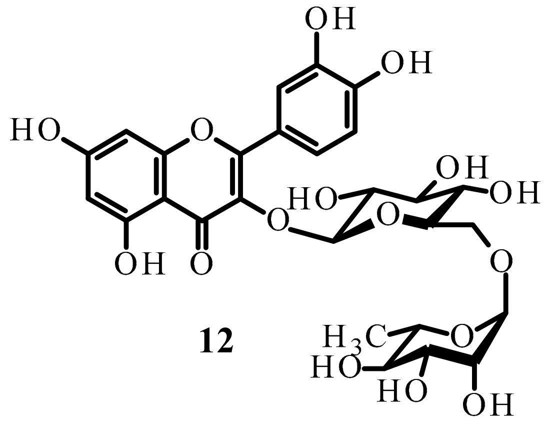

12

Qualitative Analysis Report

Spectrum Source	Fragmentor Voltage	Collision Energy	Ionization Mode
Peak (1) in "+ BPC:1 (all [-193]) Scan"	120	0	ESI

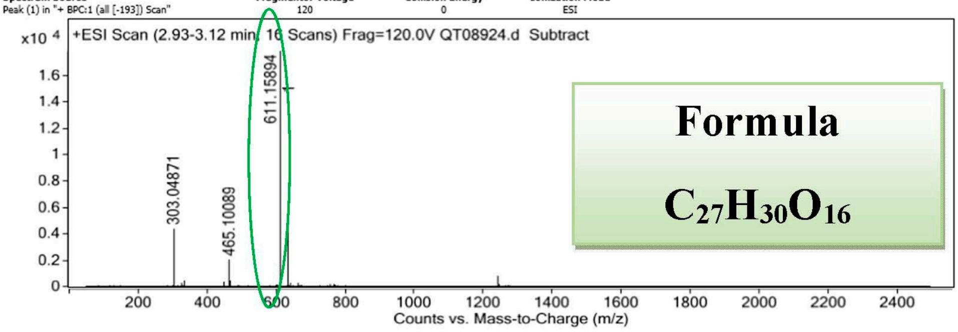

Peak List

m/z	z	Abund	Formula	Ion
303.04871		4399.5	C15 H11 O7	(M+H)+
465.10089		2087.2	C21 H21 O12	(M+H)+
611.15894	1	17895.5	C27 H31 O16	(M+H)+
612.16176	1	4753	C27 H31 O16	(M+H)+
613.16366	1	1127.5	C27 H31 O16	(M+H)+
633.1404	1	5578.7	C27 H30 Na O16	(M+Na)+
634.14371	1	1491.7	C27 H30 Na O16	(M+Na)+

Formula Calculator Results

Formula	Best	Mass	Tgt Mass	Diff (ppm)	Mz	Ion Species	Score
C22 H30 N2 O18		610.15118	610.14936	-2.98	633.1404	C22 H30 N2 Na O18	95.5
C27 H30 O16	TRUE	610.15118	610.15338	3.62	633.1404	C27 H30 Na O16	91.97
C31 H30 O11 S		610.15118	610.15088	-0.49	633.1404	C31 H30 Na O11 S	88.51
C19 H34 N2 O18 S		610.15119	610.15273	2.54	633.1404	C19 H34 N2 Na O18 S	87.51
C32 H26 N4 O7 S		610.15119	610.15222	1.69	633.1404	C32 H26 N4 Na O7 S	84.18
C23 H34 N2 O13 S2		610.15119	610.15023	-1.57	633.1404	C23 H34 N2 Na O13 S2	82.51
C27 H30 O16	TRUE	610.15166	610.15338	2.83	611.15894	C27 H31 O16	93.54
C22 H30 N2 O18		610.15166	610.14936	-3.76	611.15894	C22 H31 N2 O18	92.68
C19 H34 N2 O18 S		610.15166	610.15273	1.76	611.15894	C19 H35 N2 O18 S	88.65
C31 H30 O11 S		610.15166	610.15088	-1.27	611.15894	C31 H31 O11 S	87.09
C32 H26 N4 O7 S		610.15166	610.15222	0.91	611.15894	C32 H27 N4 O7 S	84.82
C23 H34 N2 O13 S2		610.15166	610.15023	-2.35	611.15894	C23 H35 N2 O13 S2	80.33
C21 H20 O12	TRUE	464.09361	464.09548	4.02	465.10089	C21 H21 O12	93.12
C25 H20 O7 S		464.09361	464.09297	-1.38	465.10089	C25 H21 O7 S	89.29
C16 H20 N2 O14		464.09361	464.09145	-4.66	465.10089	C16 H21 N2 O14	89.1
C26 H16 N4 O3 S		464.09362	464.09431	1.5	465.10089	C26 H17 N4 O3 S	86.05
C13 H24 N2 O14 S		464.09362	464.09482	2.6	465.10089	C13 H25 N2 O14 S	83.01
C15 H10 O7	TRUE	302.04143	302.04265	4.03	303.04871	C15 H11 O7	95.35
C19 H10 O2 S		302.04144	302.04015	-4.26	303.04871	C19 H11 O2 S	83.83
C7 H14 N2 O9 S		302.04144	302.042	1.86	303.04871	C7 H15 N2 O9 S	80.49

Figure S82. ESI-HRMS(+) of rutin.

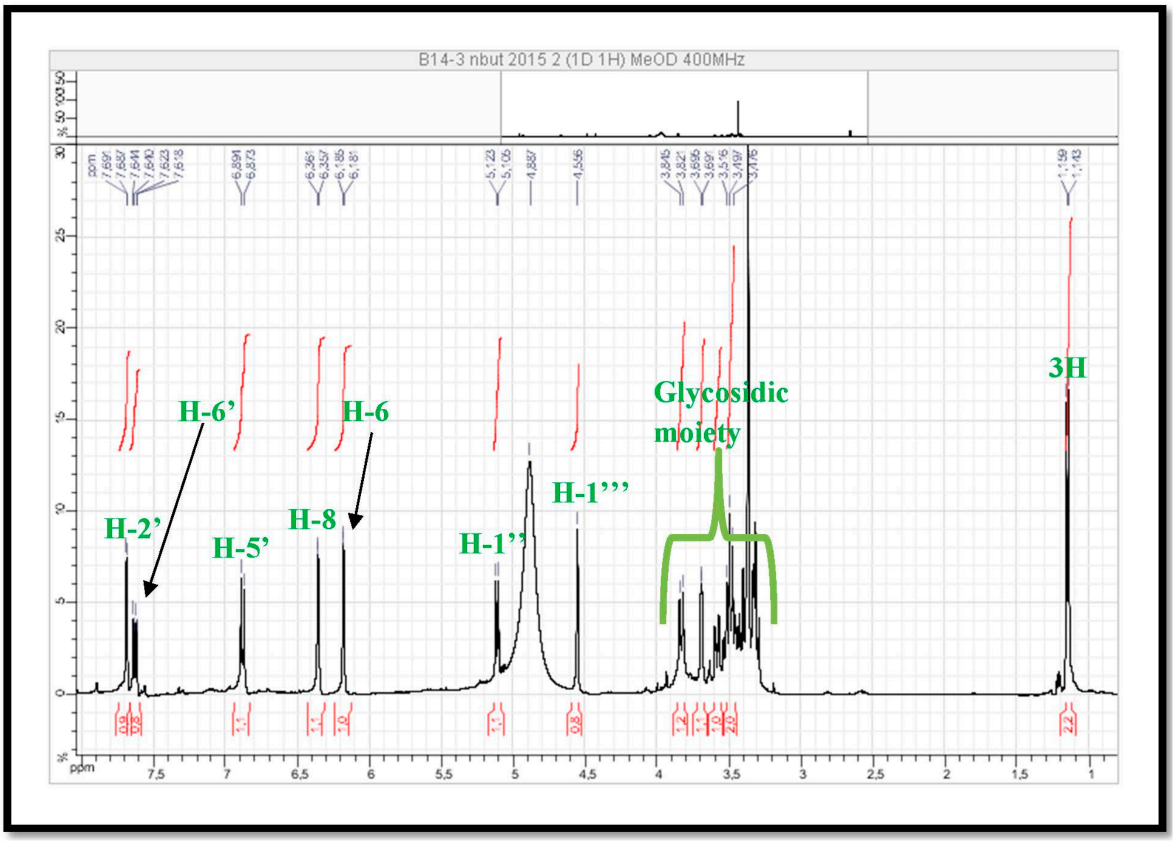

Figure S83. ^{1}H NMR spectrum (400 MHz, CD_3OD, δppm) of rutin.

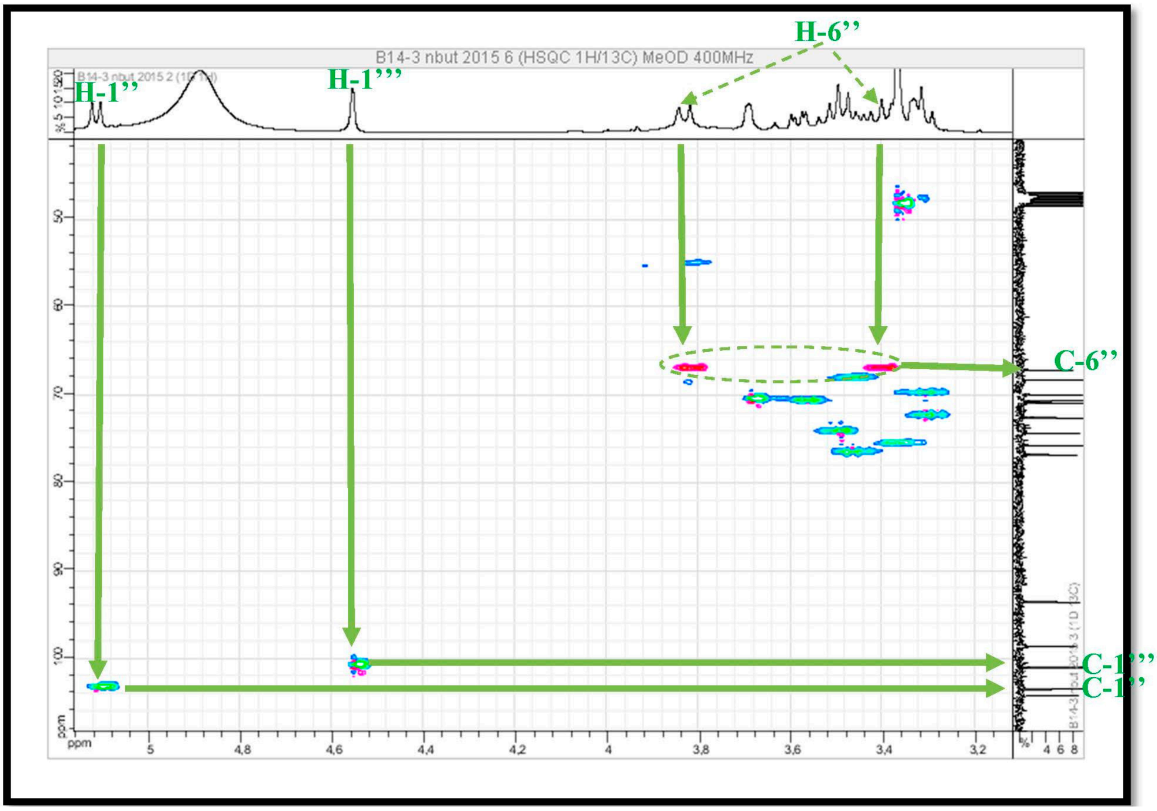

Figure S84. HSQC NMR spectrum (spreading out 1) (400 MHz, CD_3OD, δppm) of rutin.

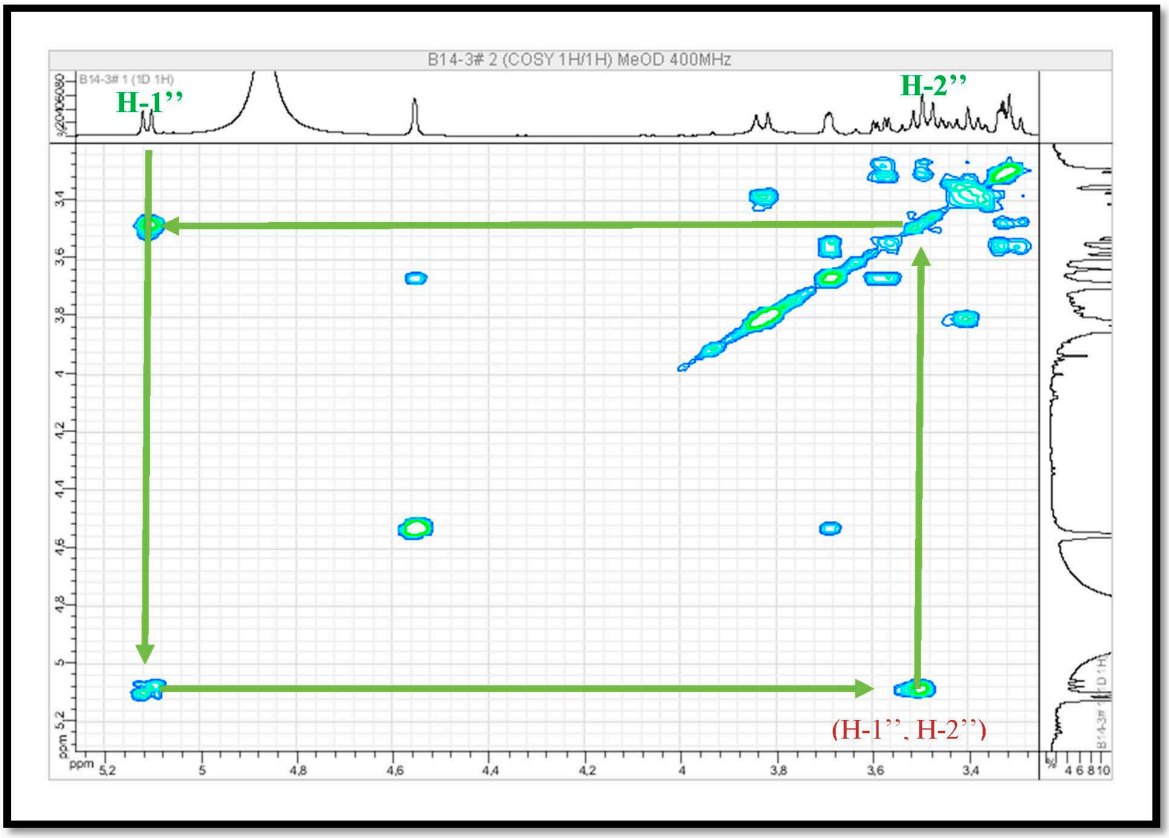

Figure S85. COSY NMR spectrum (spreading out 1) (400 MHz, CD_3OD, δppm) of rutin.

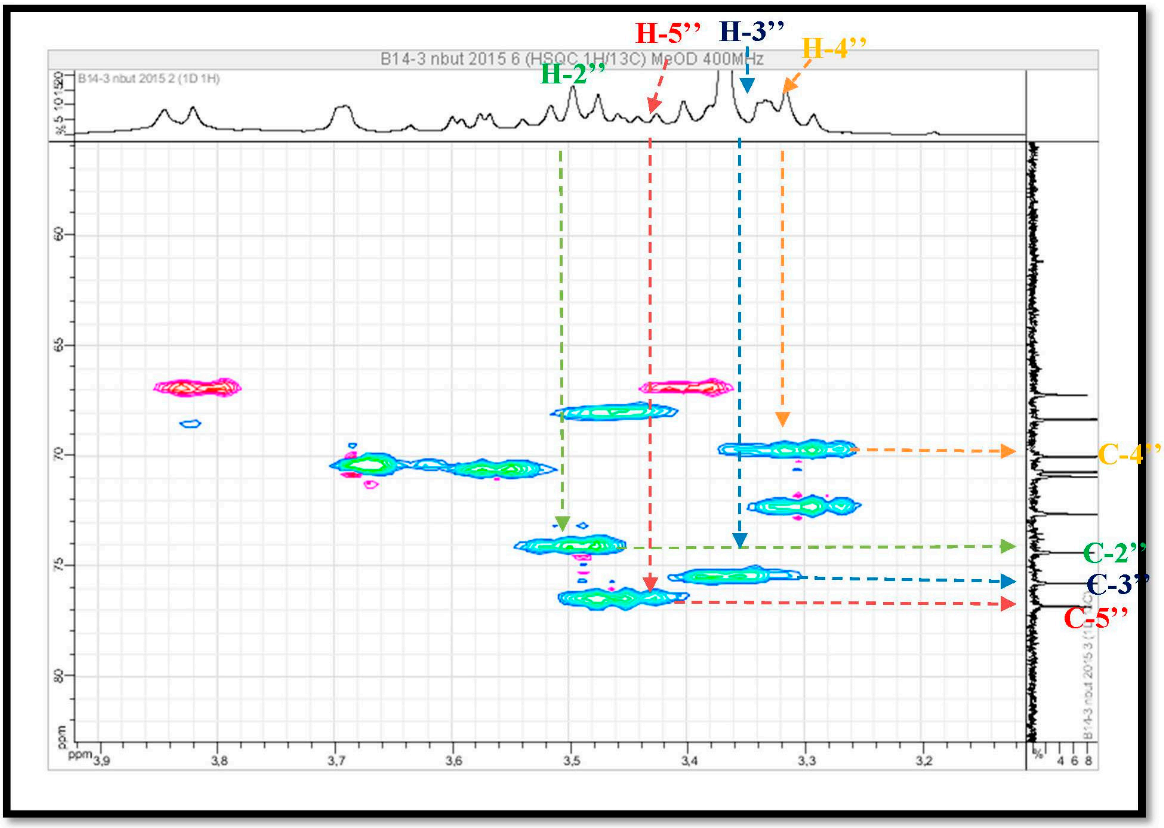

Figure S86. HSQC NMR spectrum (spreading out 2) (400 MHz, CD_3OD, δppm) of rutin.

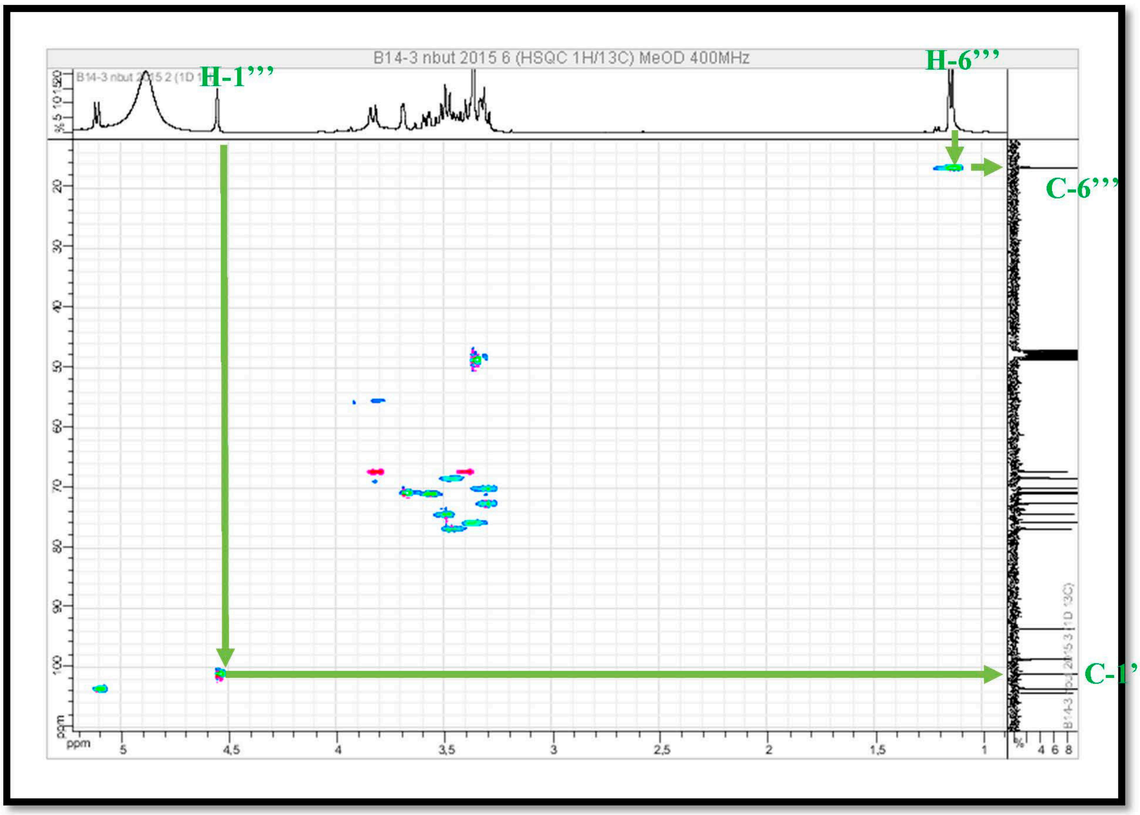

Figure S87. HSQC NMR spectrum (spreading out 3) (400 MHz, CD_3OD, δppm) of rutin.

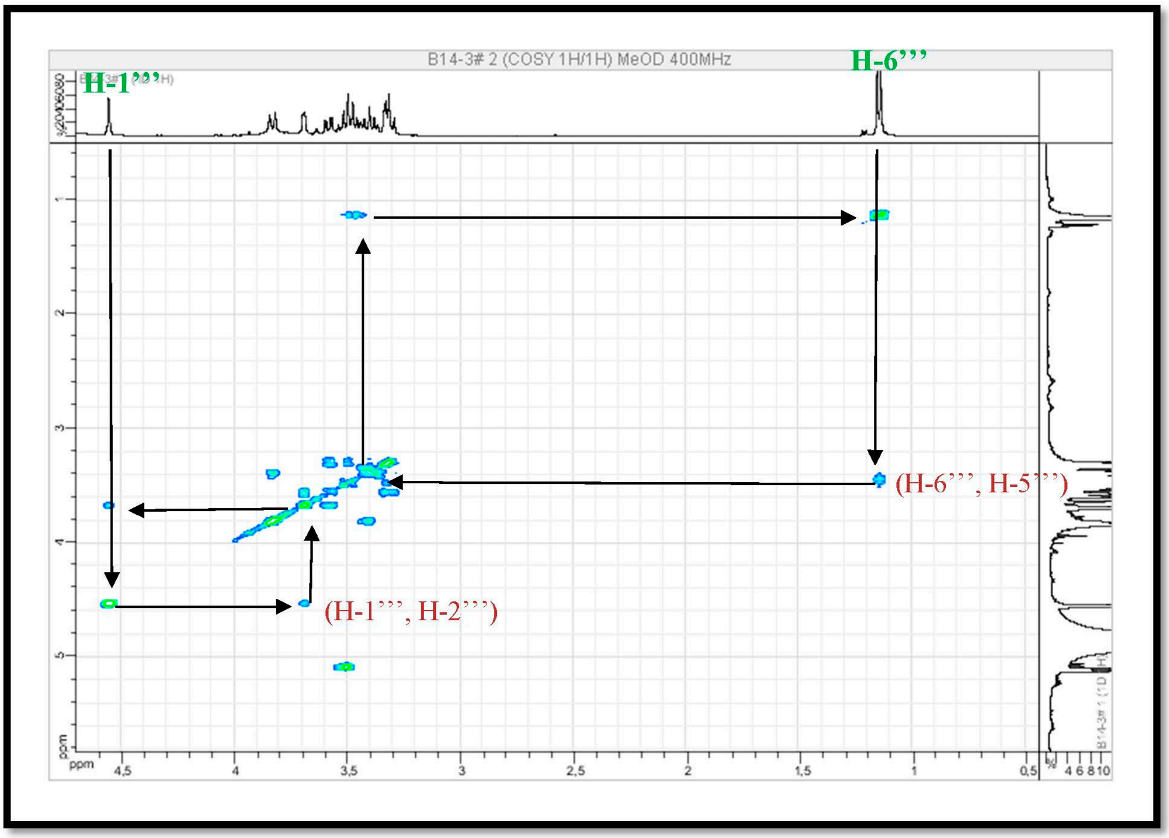

Figure S88. COSY NMR spectrum (spreading out 2) (400 MHz, CD_3OD, δppm) of rutin.

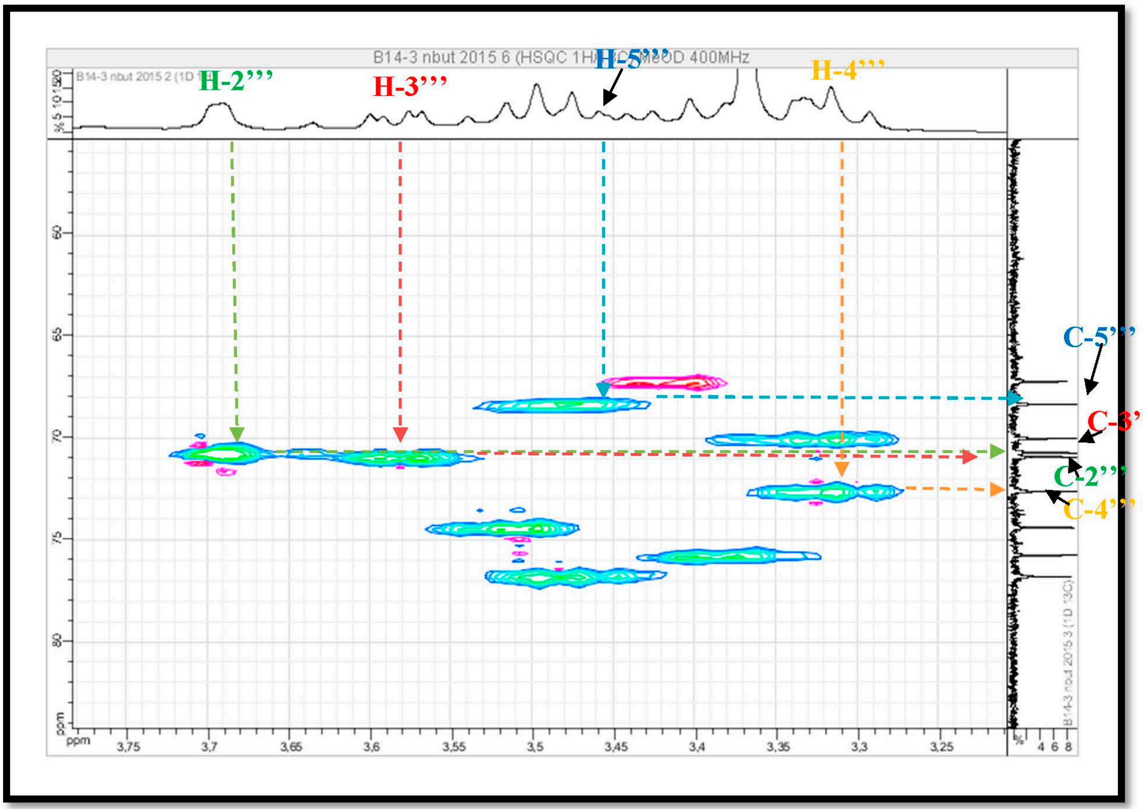

Figure S89. HMBC NMR spectrum (spreading out 1) (400 MHz, CD_3OD, δppm) of rutin.

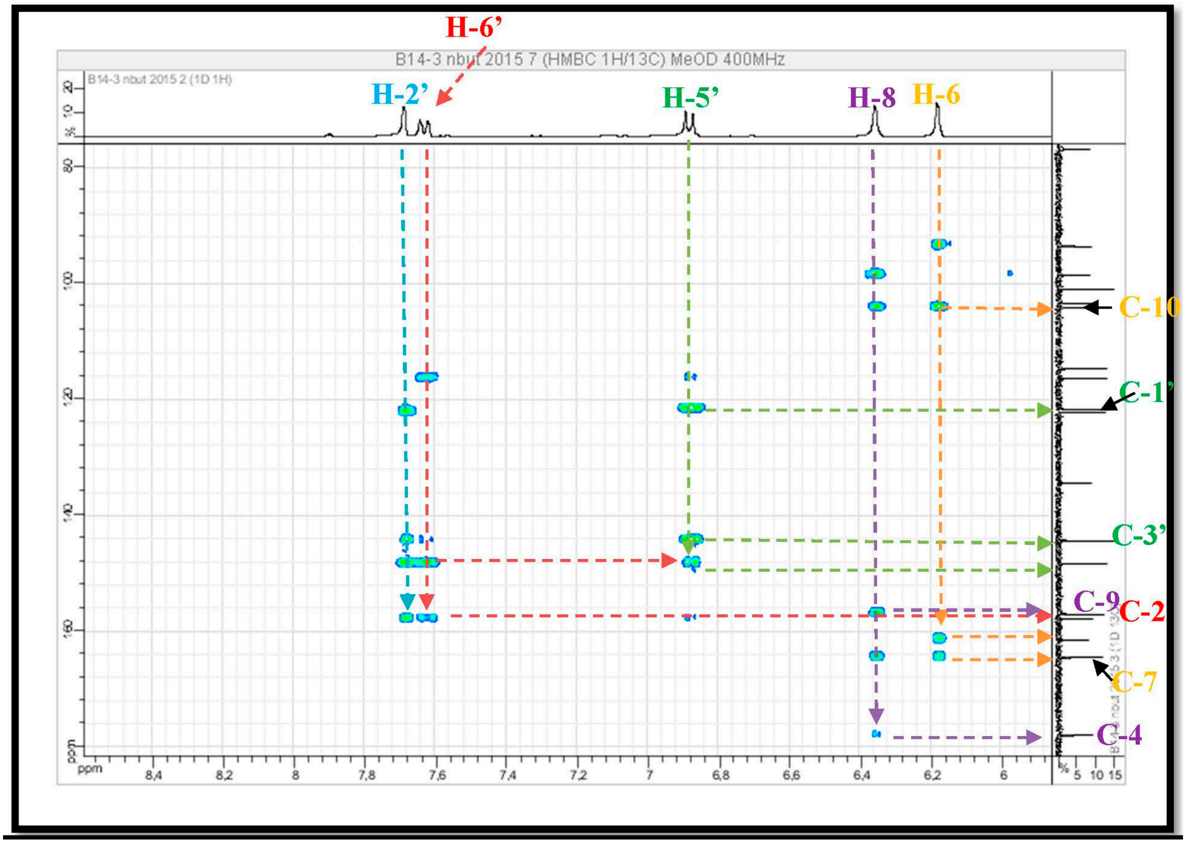

Figure S90. HMBC NMR spectrum (spreading out 2) (400 MHz, CD_3OD, δppm) of rutin.

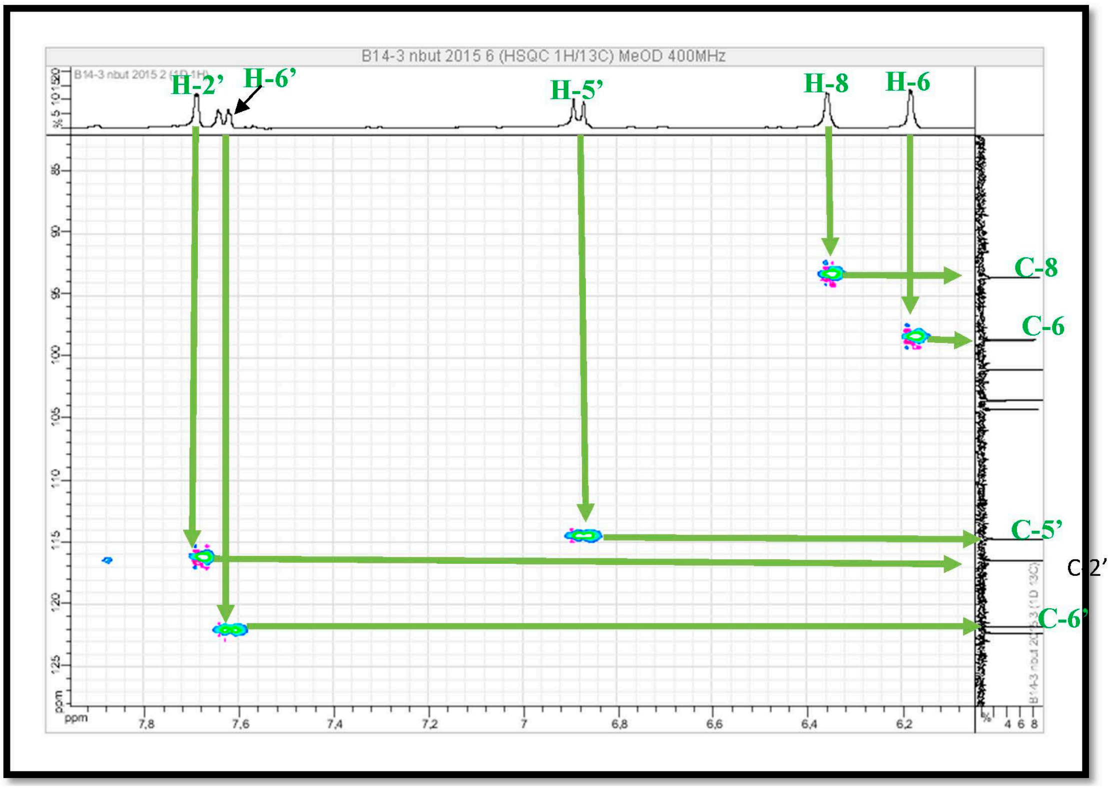

Figure S91. HMBC NMR spectrum (spreading out 4) (400 MHz, CD_3OD, δppm) of rutin.

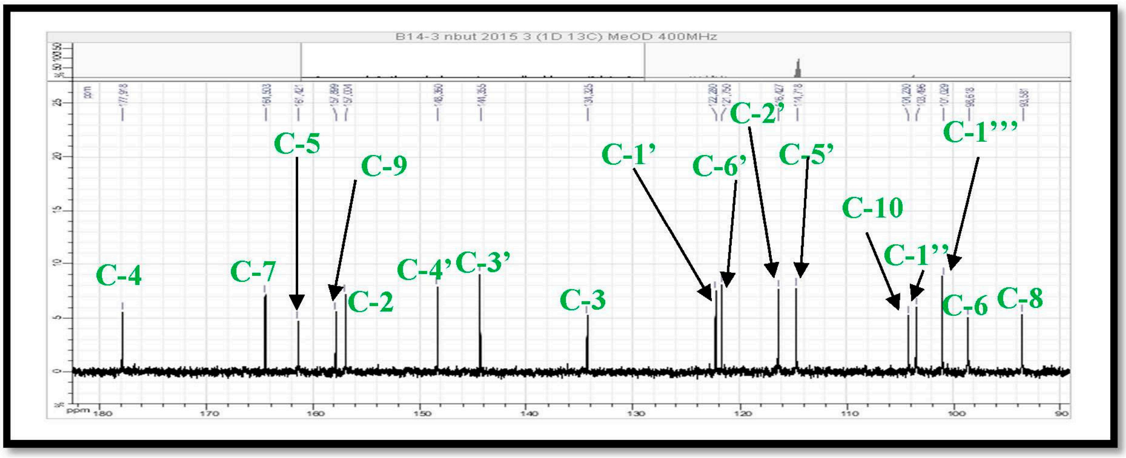

Figure S92. ^{13}CNMR spectrum (100 MHz, CD_3OD, δppm) of rutin.

Molecule 13: Vicenin-2

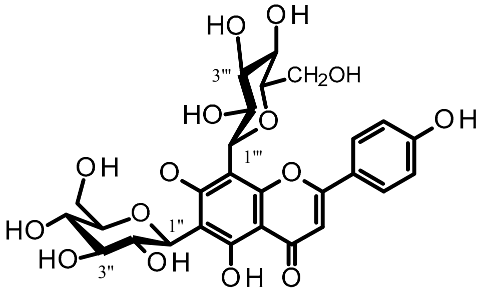

13

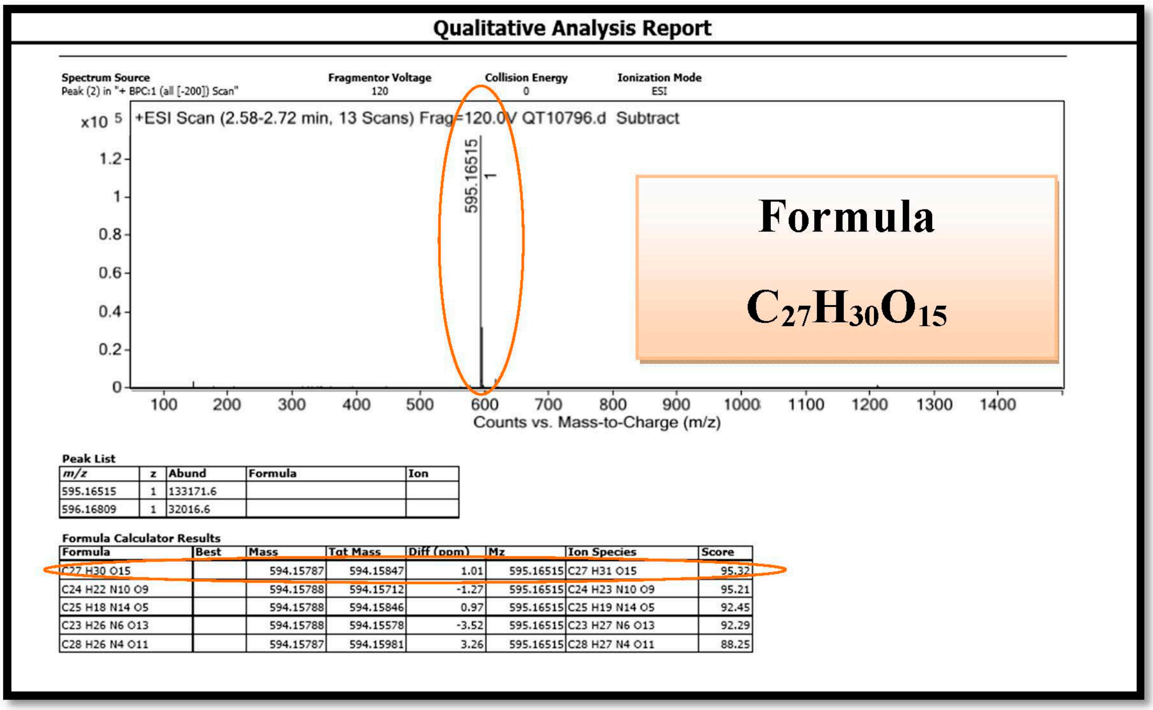

Peak List

m/z	z	Abund	Formula	Ion
595.16515	1	133171.6		
596.16809	1	32016.6		

Formula Calculator Results

Formula	Best	Mass	Tgt Mass	Diff (ppm)	Mz	Ion Species	Score
C27 H30 O15		594.15787	594.15847	1.01	595.16515	C27 H31 O15	95.32
C24 H22 N10 O9		594.15788	594.15712	-1.27	595.16515	C24 H23 N10 O9	95.21
C25 H18 N14 O5		594.15788	594.15846	0.97	595.16515	C25 H19 N14 O5	92.45
C23 H26 N6 O13		594.15788	594.15578	-3.52	595.16515	C23 H27 N6 O13	92.29
C28 H26 N4 O11		594.15787	594.15981	3.26	595.16515	C28 H27 N4 O11	88.25

Figure S93. ESI-HRMS(+) of vicenin-2.

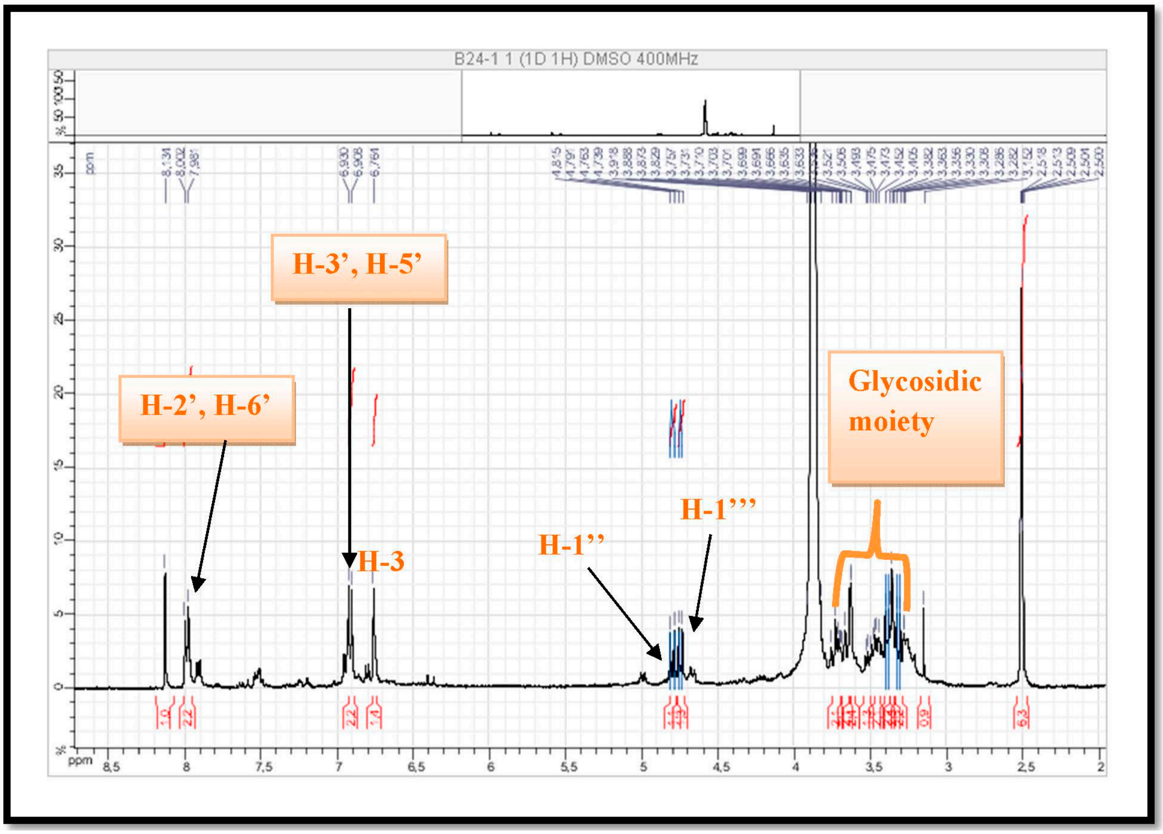

Figure S94. ^{1}H NMR spectrum (400 MHz, DMSO-d_6, δppm) of vicenin-2.

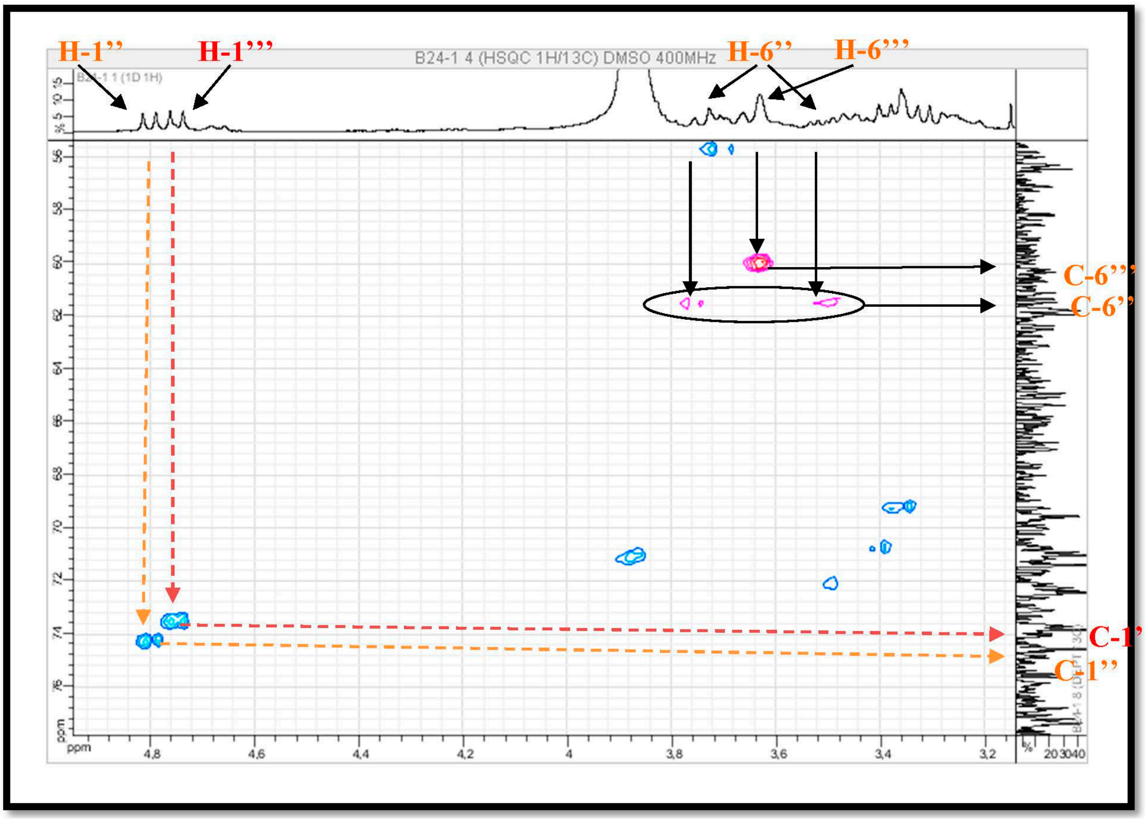

Figure S95. HSQC NMR spectrum (spreading out 1) (400 MHz, DMSO-d_6, δppm) of vicenin-2.

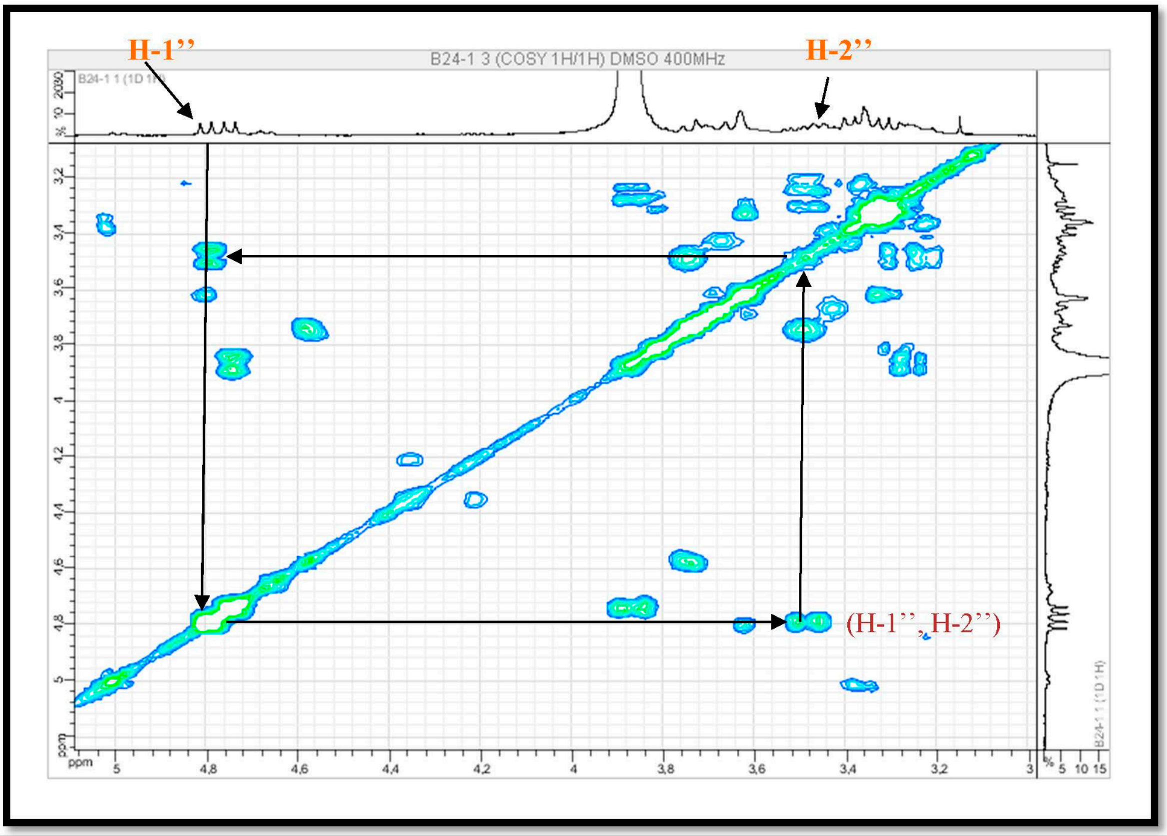

Figure S96. COSY NMR spectrum (spreading out 1) (400 MHz, DMSO-d_6, δppm) of vicenin-2.

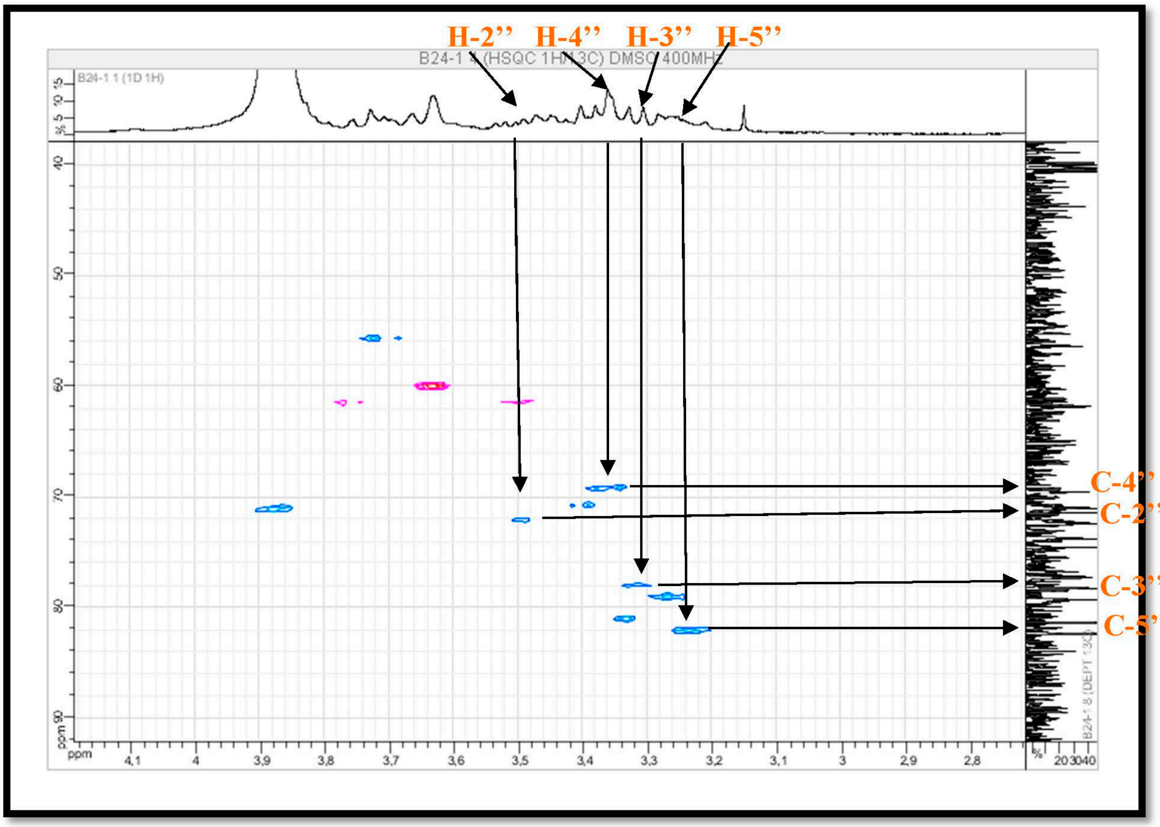

Figure S97. HSQC NMR spectrum (spreading out 2) (400 MHz, DMSO-d_6, δppm) of vicenin-2.

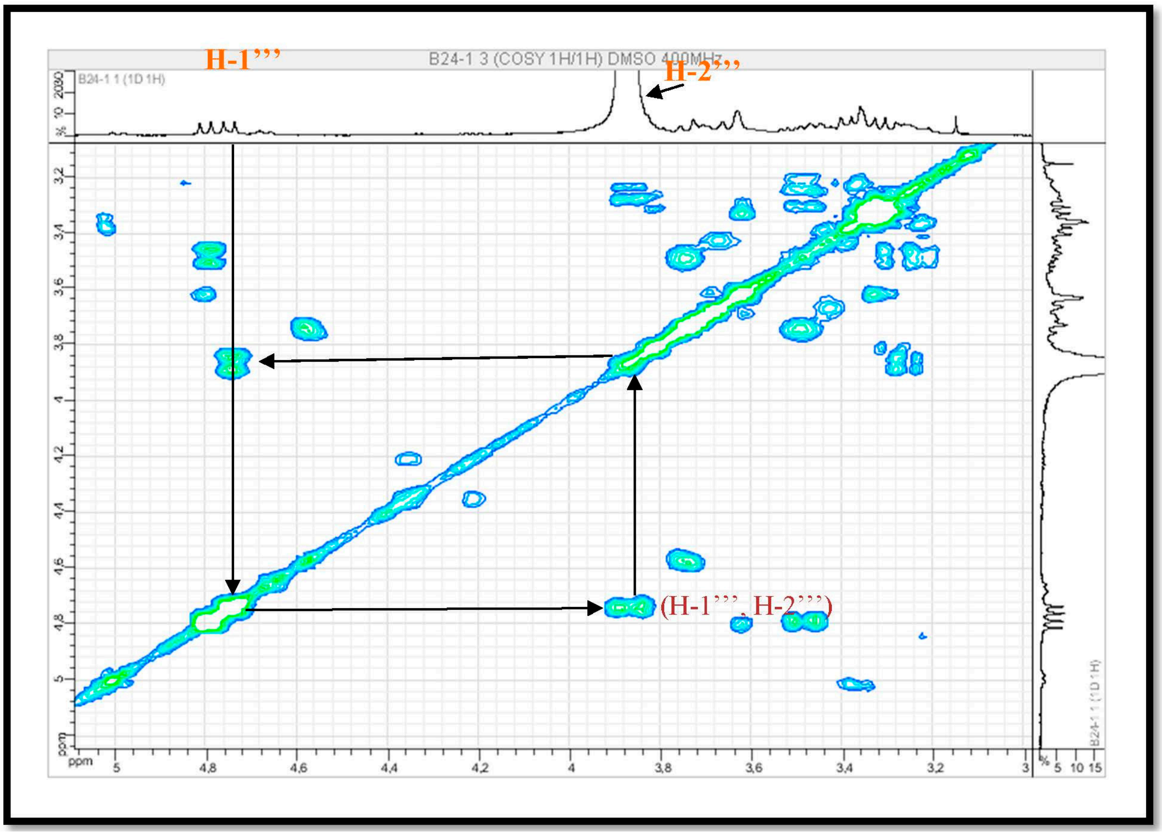

Figure S98. COSY NMR spectrum (spreading out 2) (400 MHz, DMSO-d_6, δppm) of vicenin-2.

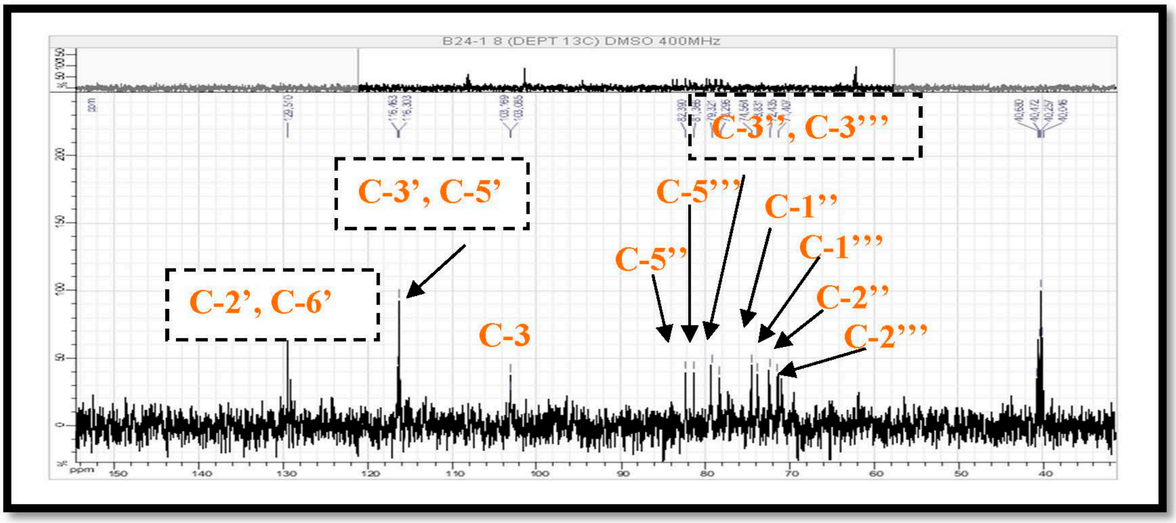

Figure S99. ^{13}C NMR DEPT spectrum (100 MHz, DMSO-d_6, δppm) of vicenin-2.

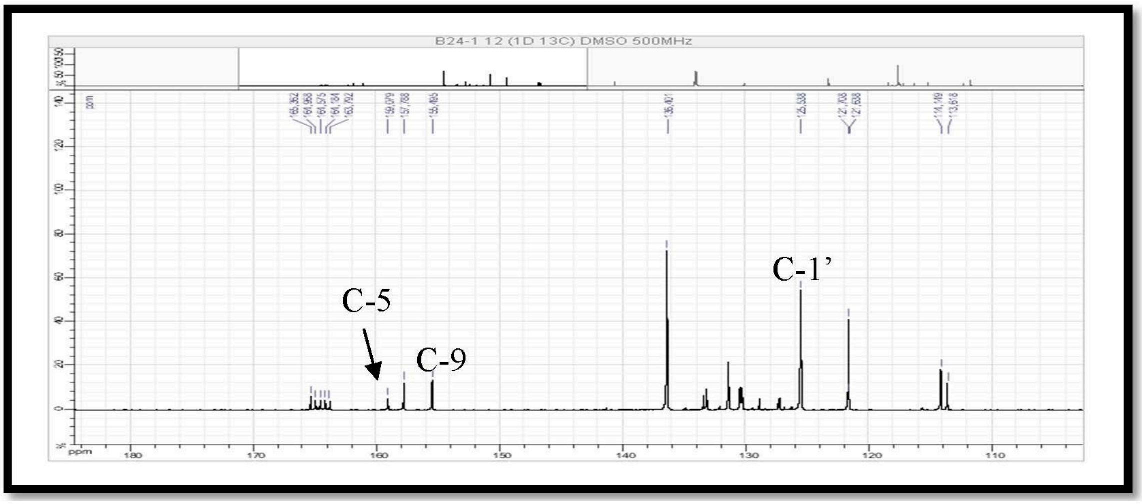

Figure S100. ^{13}C NMR spectrum (100 MHz, DMSO-d_6, δppm) of vicenin-2.

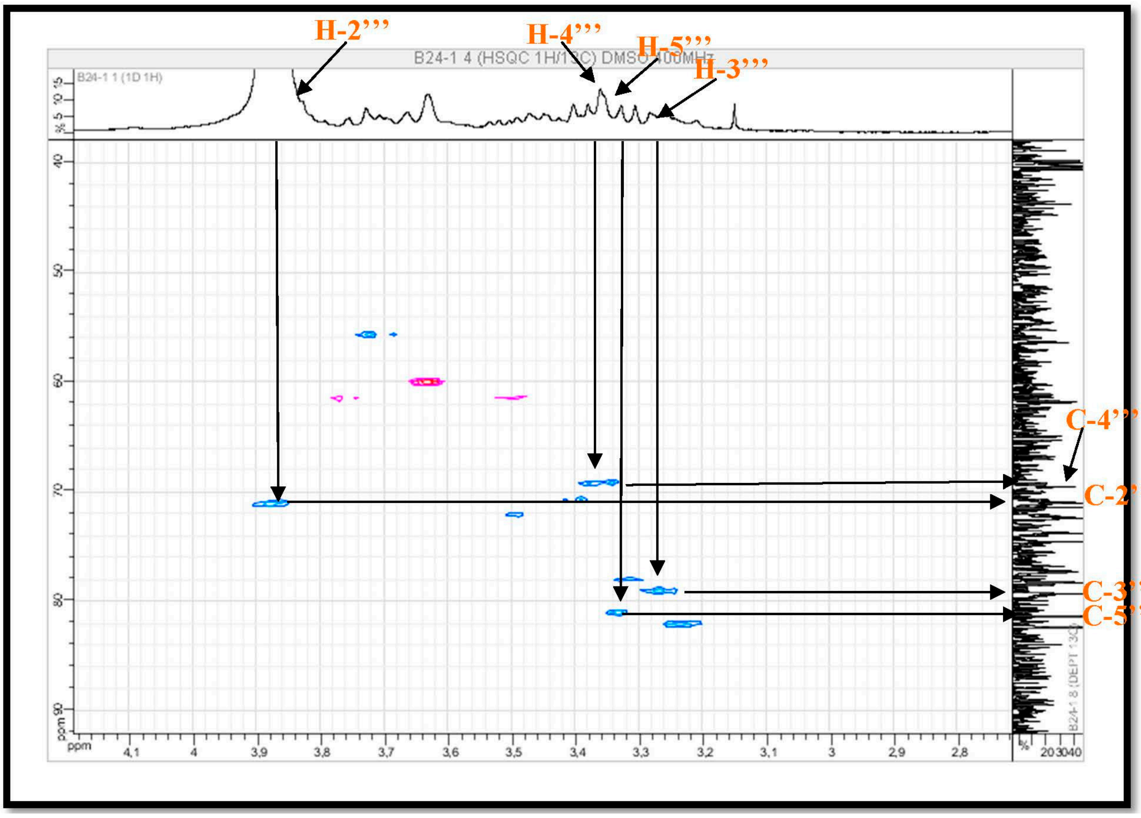

Figure S101. HSQC NMR spectrum (spreading out 3) (400 MHz, DMSO-d_6, δppm) of vicenin-2.

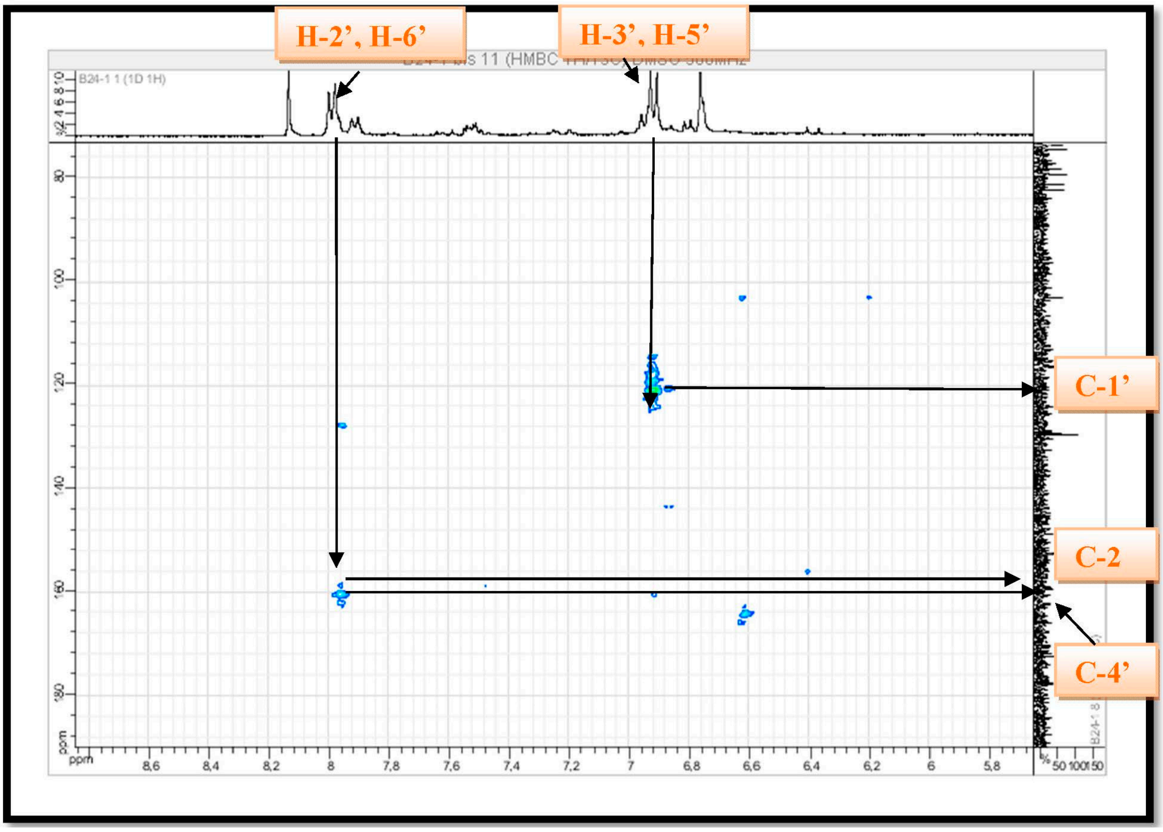

Figure S102. HMBC NMR spectrum (spreading out 1) (400 MHz, DMSO-d_6, δppm) of vicenin-2.

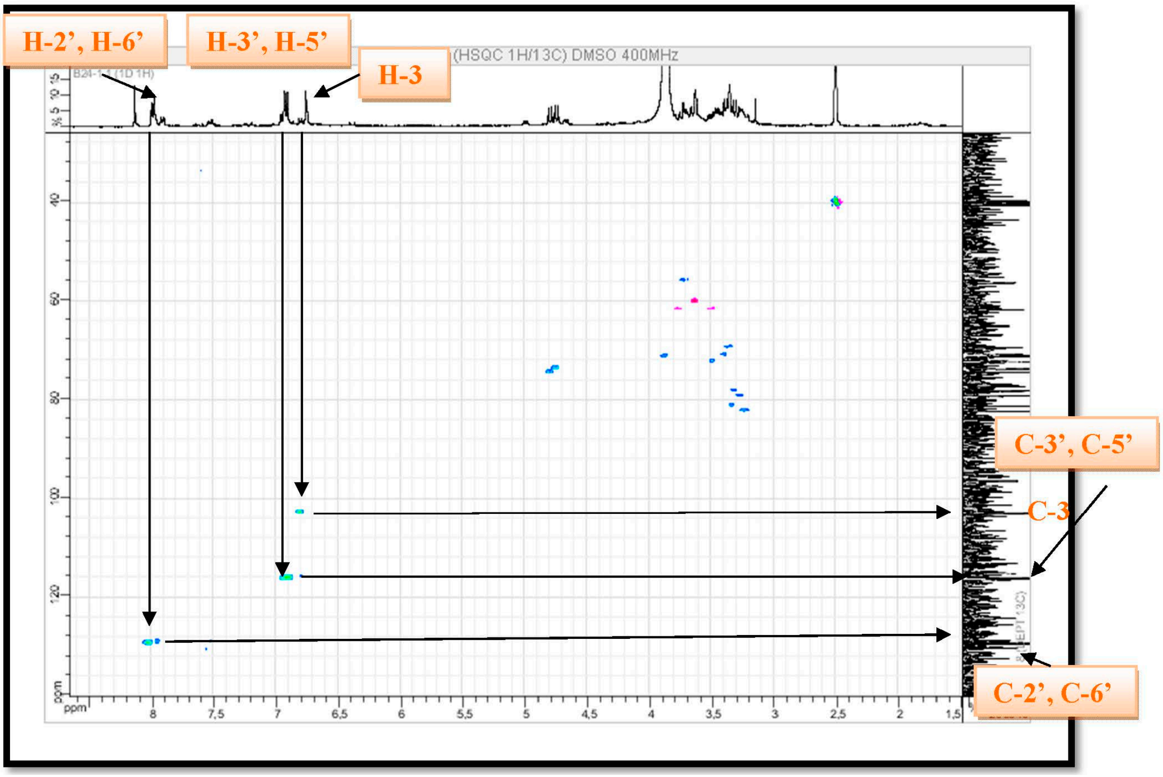

Figure S103. HSQC NMR spectrum (spreading out 4) (400 MHz, DMSO-d_6, δppm) of vicenin-2.

Molecule 14: Narcissin

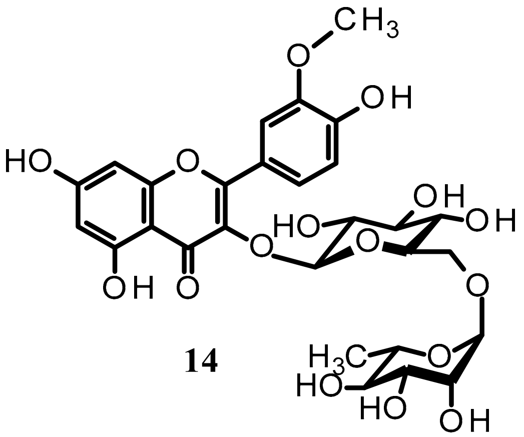

14

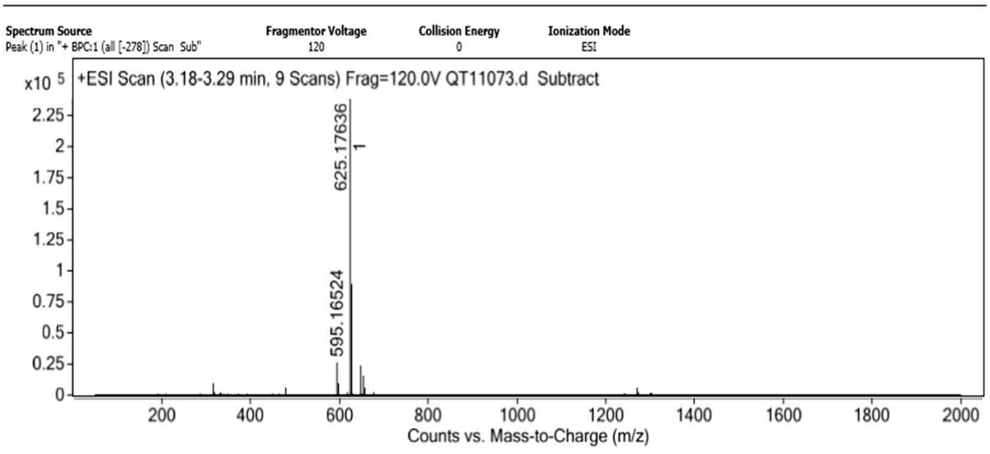

Peak List

m/z	z	Abund	Formula	Ion
595.16524		27012.5		
625.17636	1	237708	C28 H33 O16	(M+H)+
626.17986	1	89409.9	C28 H33 O16	(M+H)+
627.18278	1	21102	C28 H33 O16	(M+H)+
647.1574		24016.9		
655.18599		15304.5		

Formula Calculator Results

Formula	Best	Mass	Tgt Mass	Diff (ppm)	Mz	Ion Species	Score
C28 H32 O16	TRUE	624.16909	624.16903	-0.09	625.17636	C28 H33 O16	99.04

Figure S104. ESI-HRMS(+) of narcissin.

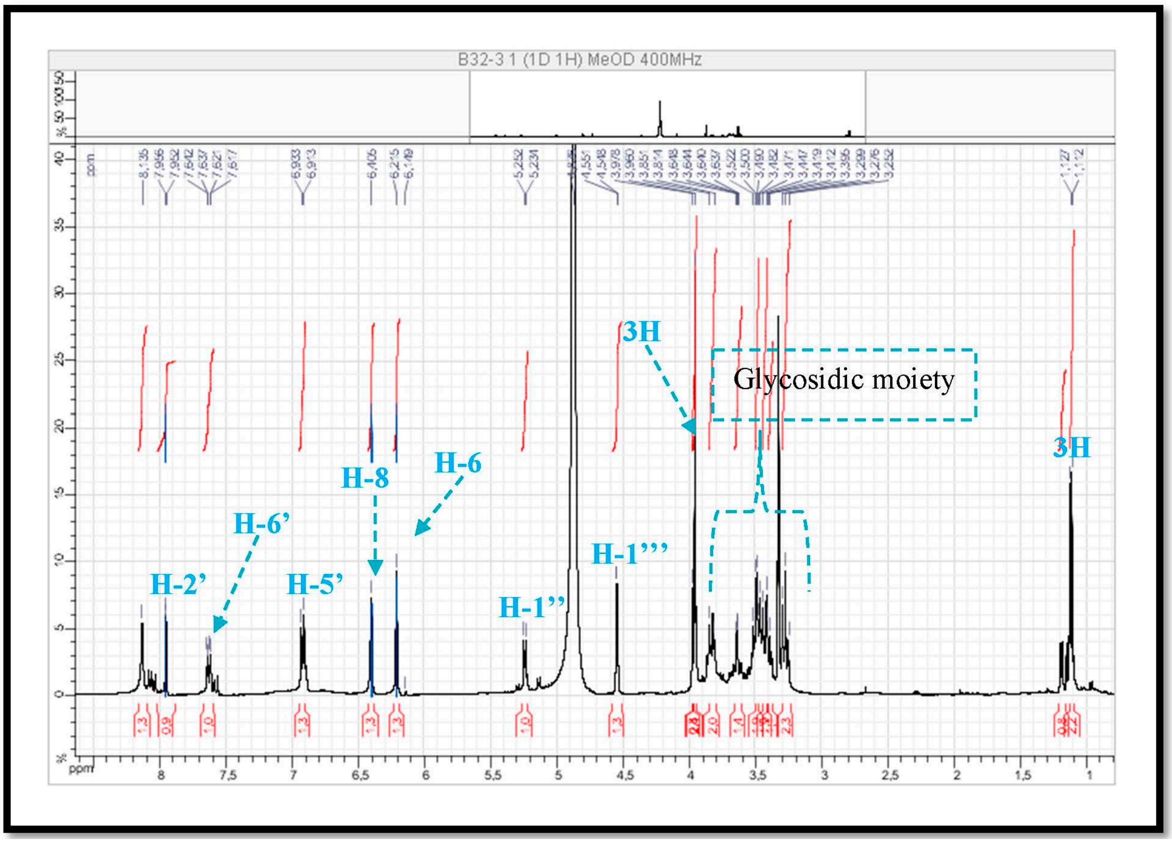

Figure S105. ^{1}H NMR spectrum (400 MHz, CD_3OD, δppm) of narcissin.

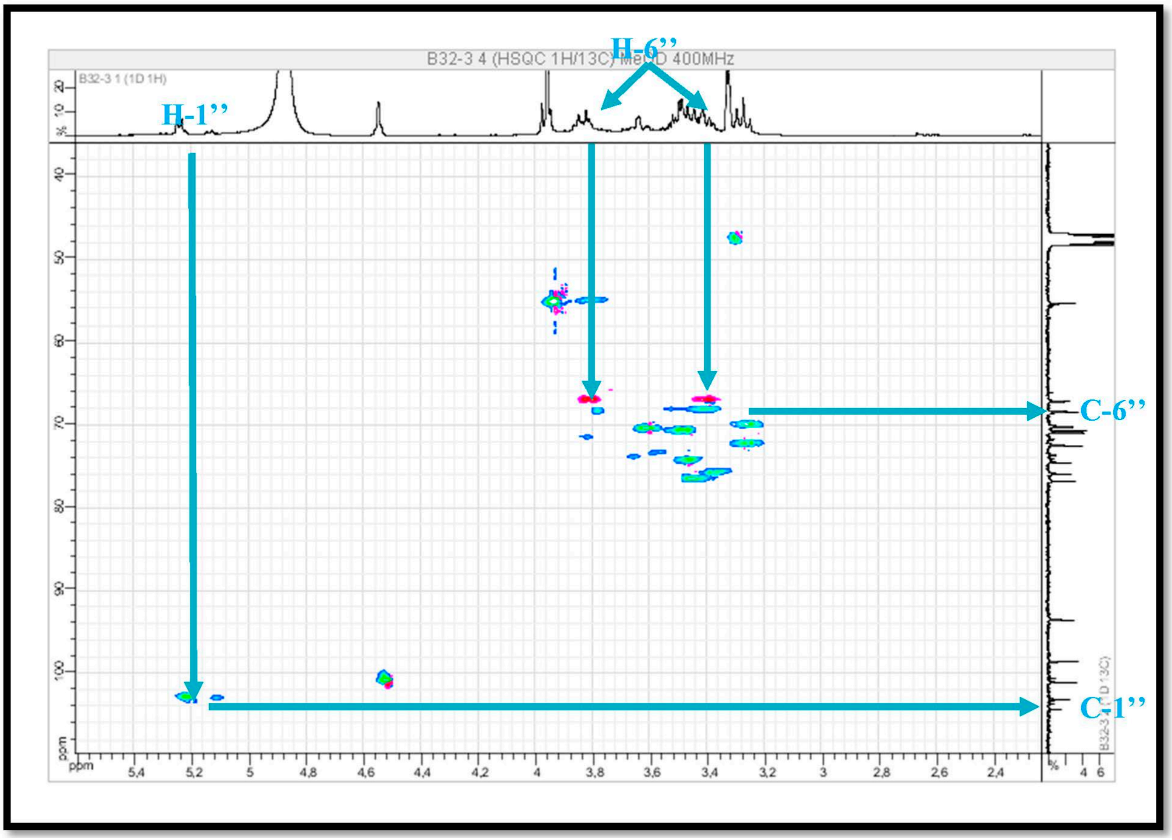

Figure S106. HSQC NMR spectrum (spreading out 1) (400 MHz, CD_3OD, δppm) of narcissin.

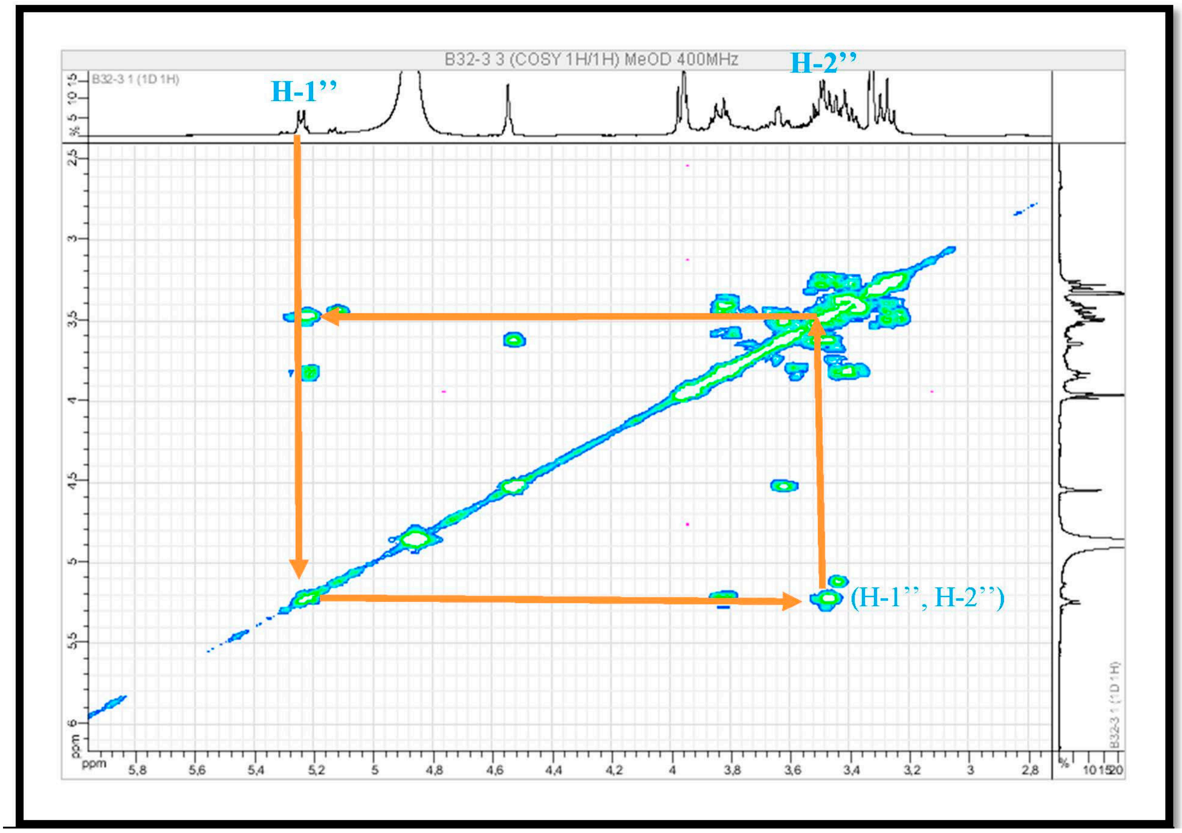

Figure S107. COSY NMR spectrum (spreading out 1) (400 MHz, CD_3OD, δppm) of narcissin.

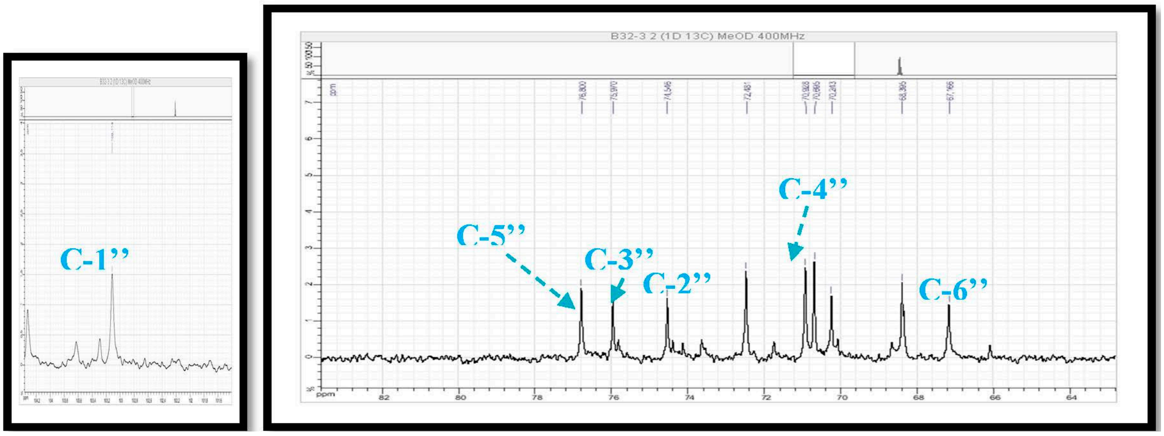

Figure S108. ^{13}C NMR spectrum (spreading out 1) (100 MHz, CD_3OD, δppm) of narcissin.

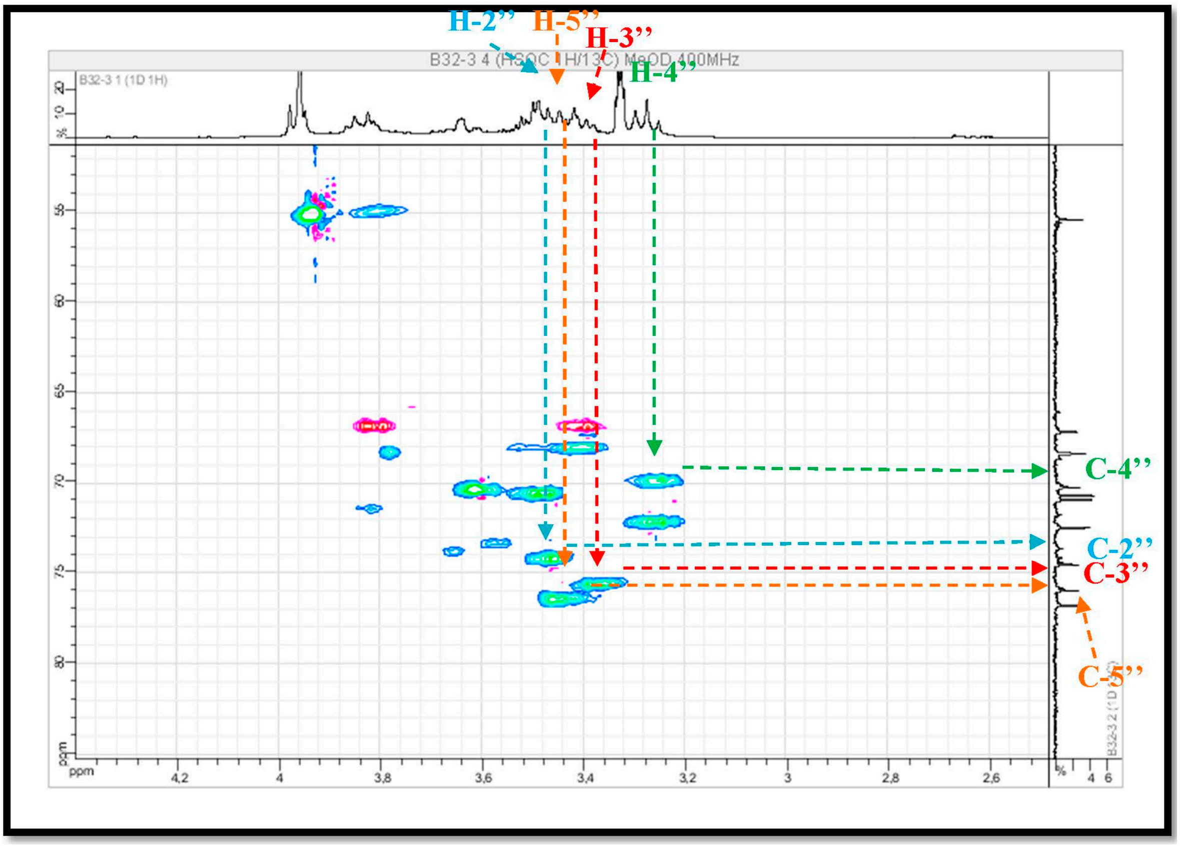

Figure S109. HSQC NMR spectrum (spreading out 2) (400 MHz, CD_3OD, δppm) of narcissin.

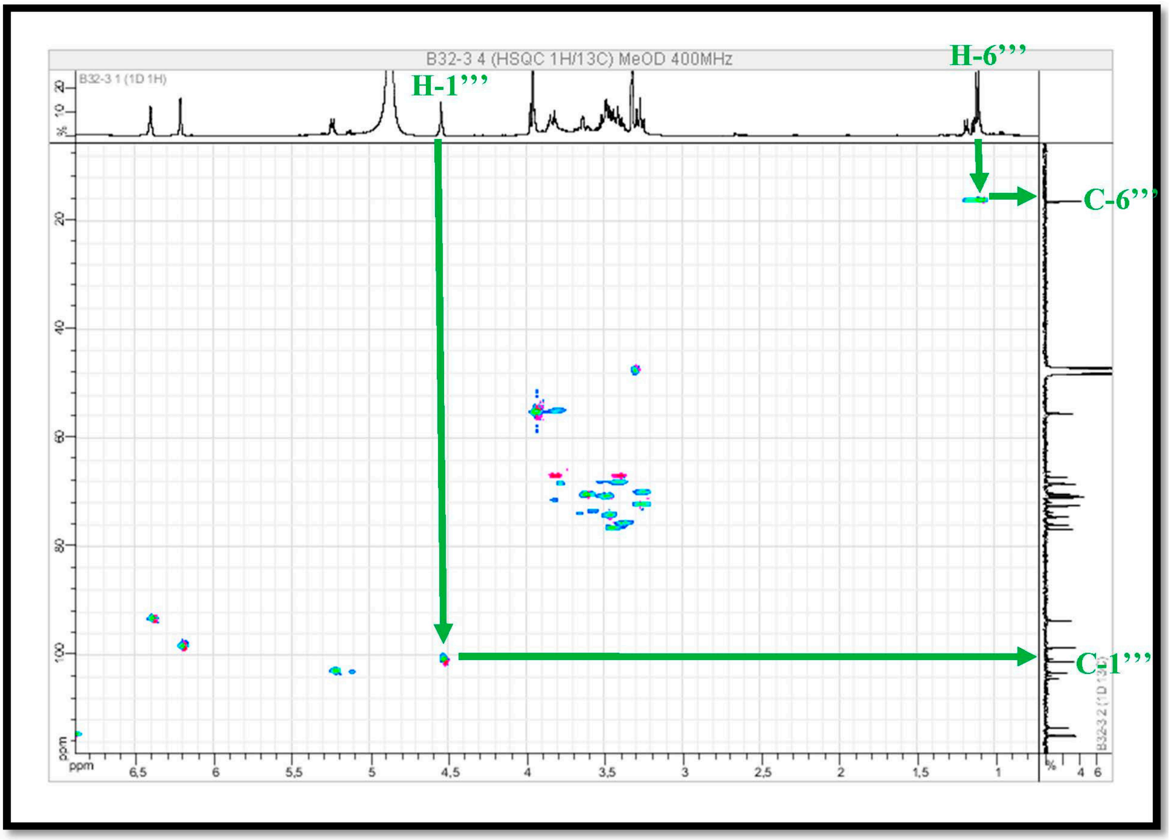

Figure S110. HSQC NMR spectrum (spreading out 3) (400 MHz, CD_3OD, δppm) of narcissin.

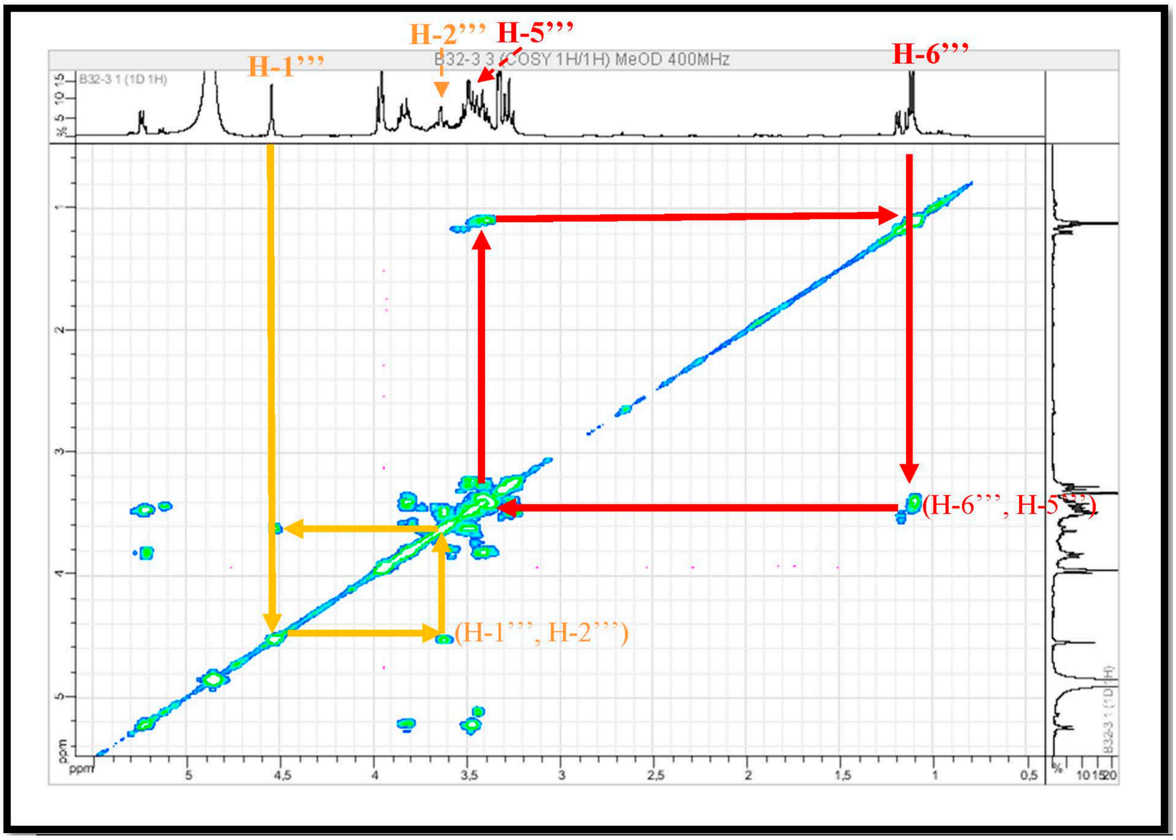

Figure S111. COSY NMR spectrum (spreading out 2) (400 MHz, CD_3OD, δppm) of narcissin.

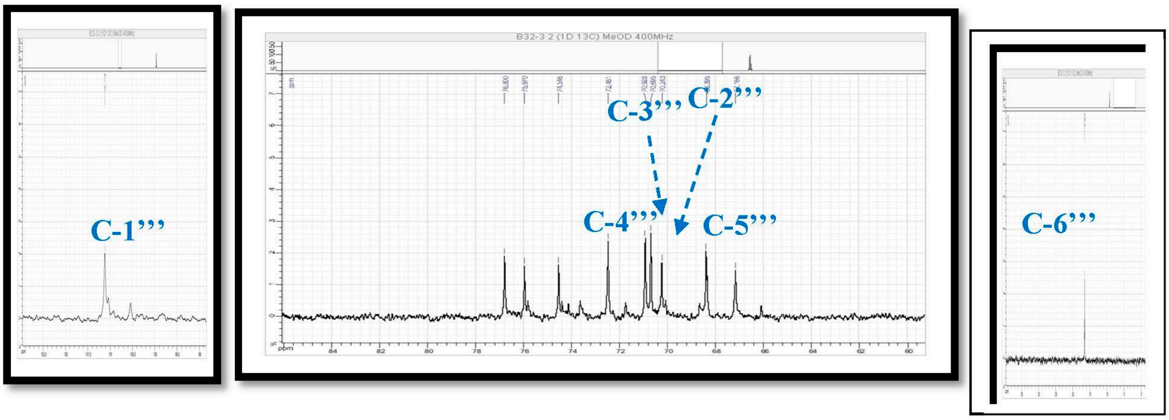

Figure S112. ^{13}C NMR spectrum (spreading out 2) (100 MHz, CD_3OD, δppm) of narcissin.

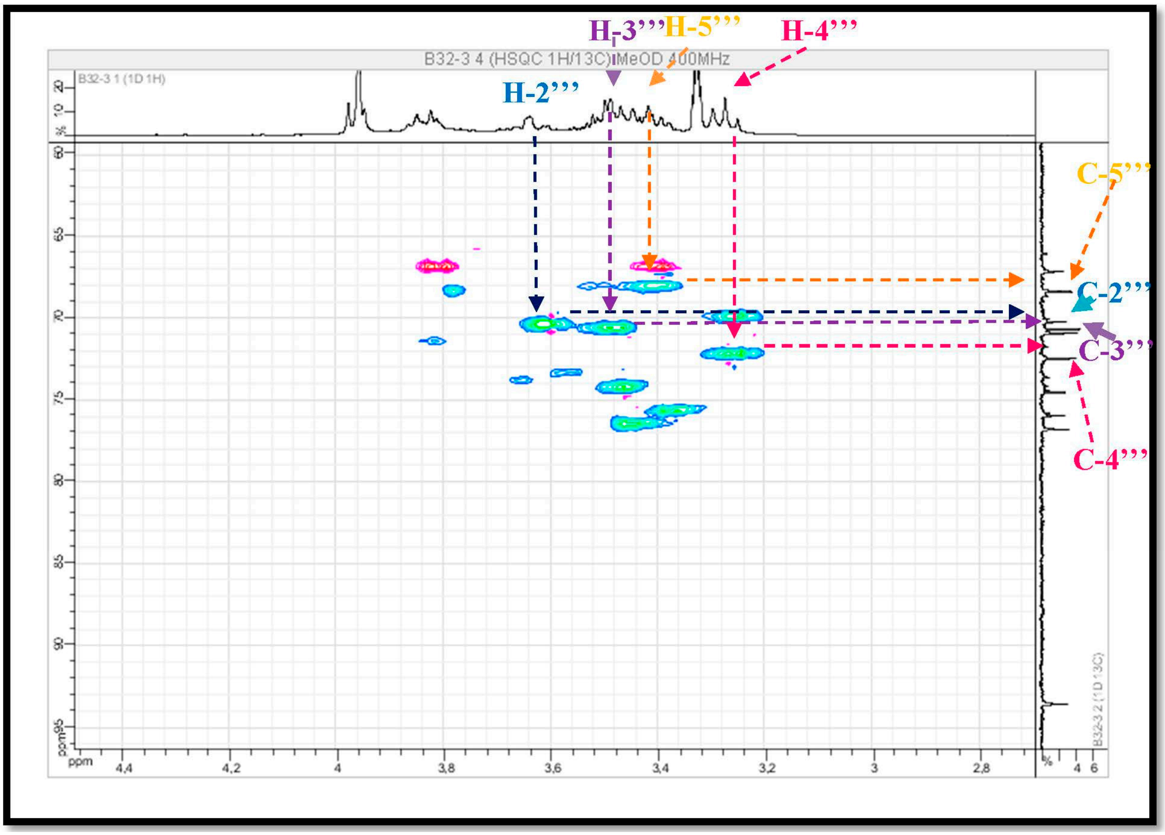

Figure S113. HSQC NMR spectrum (spreading out 4, 400 MHz, CD_3OD, δppm) of narcissin.

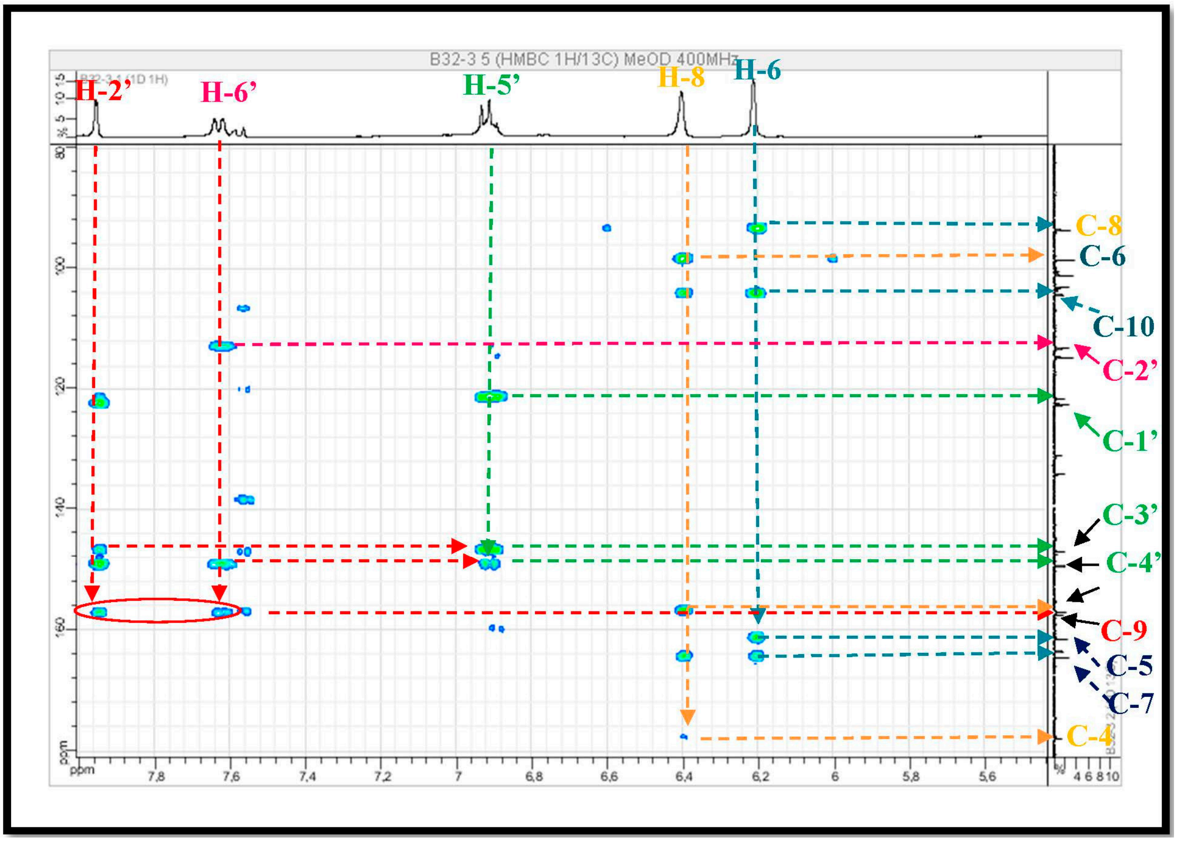

Figure S114. HMBC NMR spectrum (spreading out 1) (400 MHz, CD3OD, δppm) of narcissin.

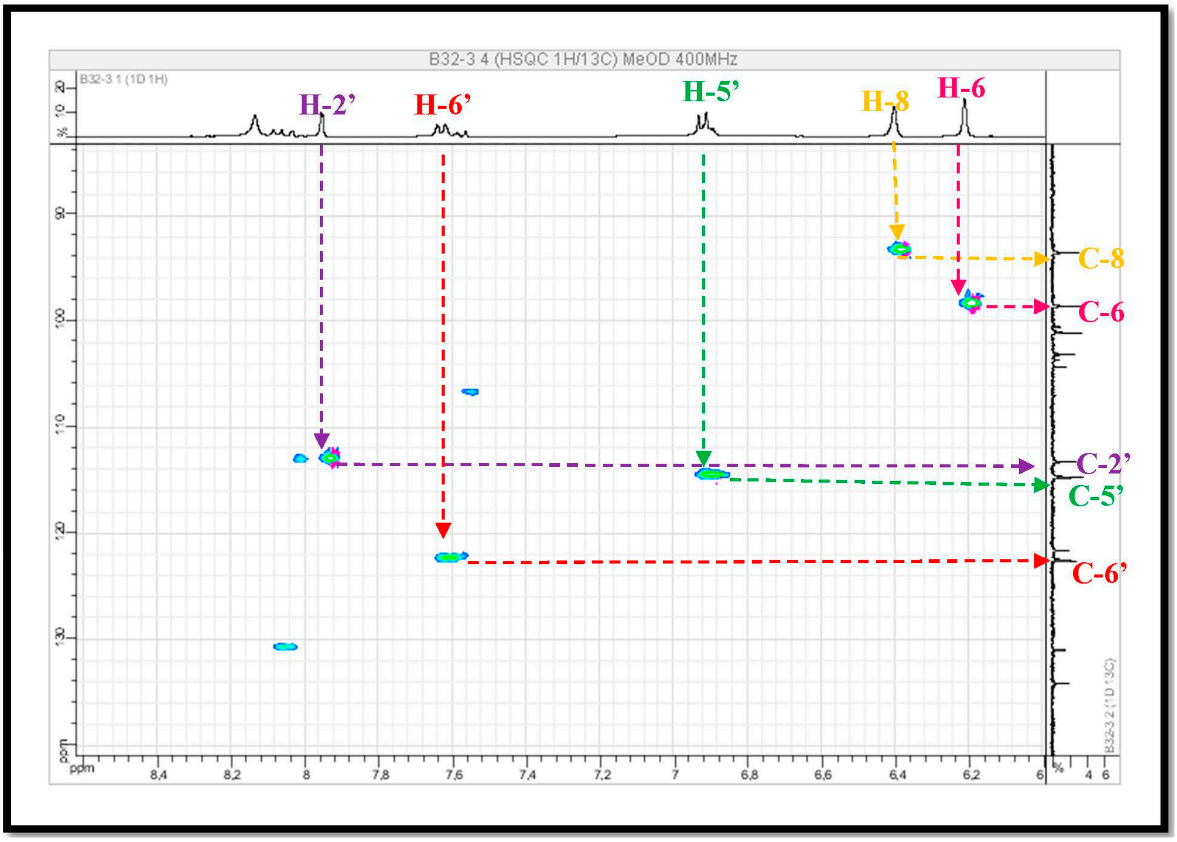

Figure S115. HSQC NMR spectrum (spreading out 5) (400 MHz, CD3OD, δppm) of narcissin.

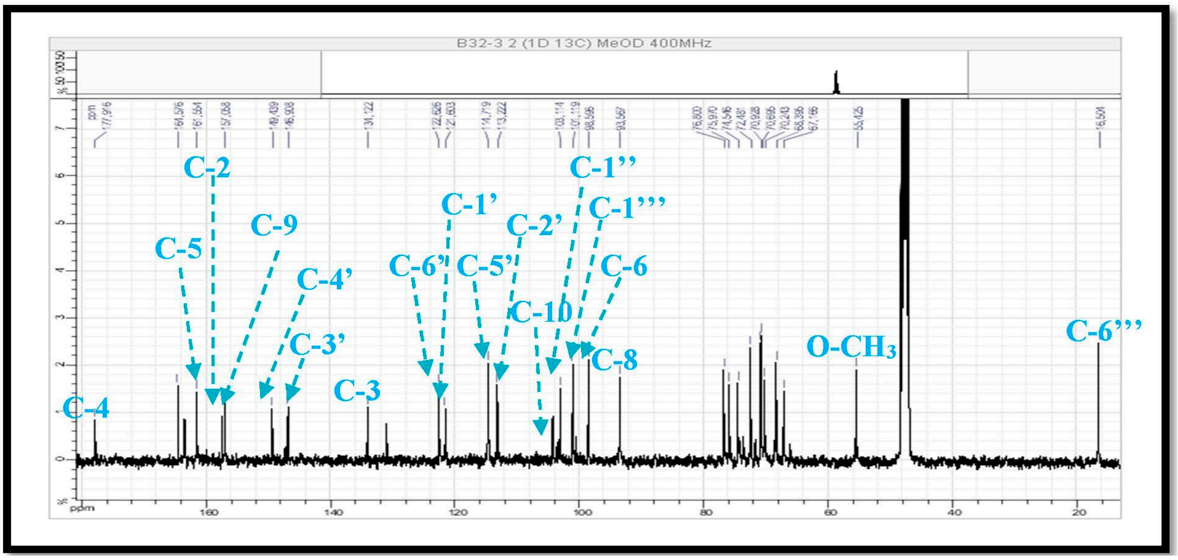

Figure S116. ^{13}C NMR spectrum (spreading out 3) (100 MHz, CD_3OD, δppm) of narcissin.

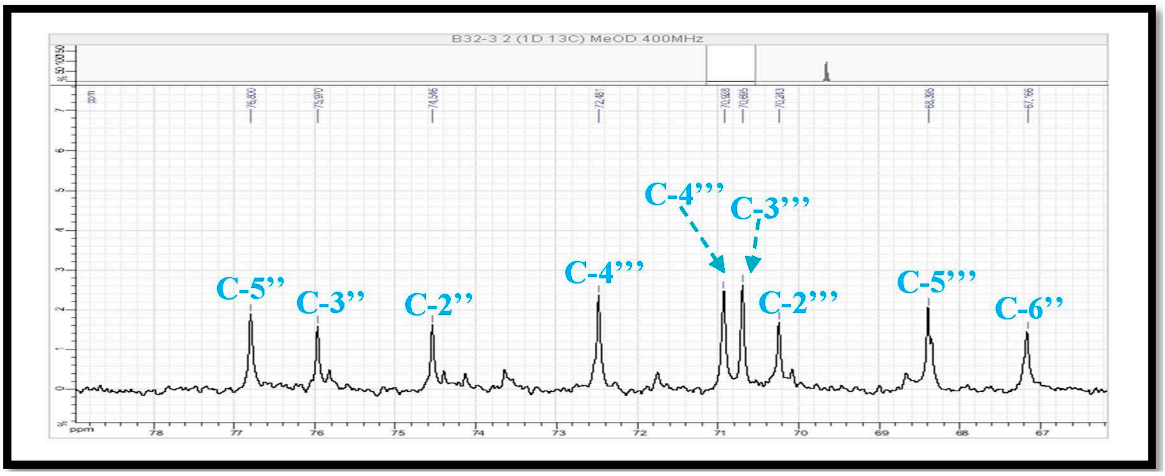

Figure S117. ^{13}C NMR spectrum (spreading out 4) (100 MHz, CD_3OD, δppm) of narcissin.
